# Microfluidic light-driven synthesis of tetracyclic molecular architectures

**DOI:** 10.3762/bjoc.14.219

**Published:** 2018-09-17

**Authors:** Javier Mateos, Nicholas Meneghini, Marcella Bonchio, Nadia Marino, Tommaso Carofiglio, Xavier Companyó, Luca Dell’Amico

**Affiliations:** 1 Dipartimento di Scienze Chimiche and ITM-CNR UoS of Padova, Università di Padova, Via Marzolo 1, 35131 Padova, Italy

**Keywords:** [4 + 2] photoenol, cycloaddition, flow chemistry, microfluidic photoreactor, photoredox catalysis, synthetic photochemistry

## Abstract

Herein we report an effective synthetic method for the direct assembly of highly functionalized tetracyclic pharmacophoric cores. Coumarins and chromones undergo diastereoselective [4 + 2] cycloaddition reactions with light-generated photoenol intermediates. The reactions occur by aid of a microfluidic photoreactor (MFP) in high yield (up to >98%) and virtually complete diastereocontrol (>20:1 dr). The method is easily scaled-up to a parallel setup, furnishing 948 mg of product over a 14 h reaction time. Finally, a series of manipulations of the tetracyclic scaffold obtained gave access to valuable precursors of biologically active molecules.

## Introduction

**Figure 1:**
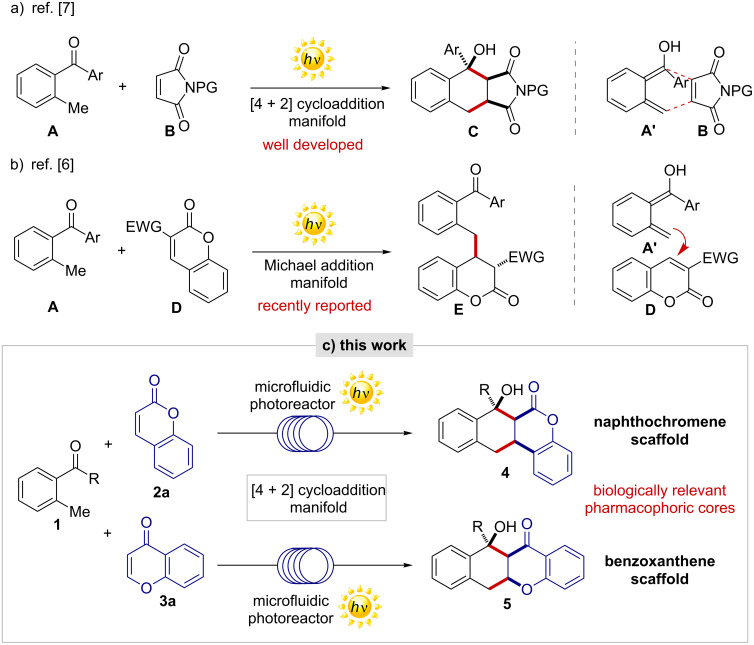
a) Light-driven reaction between 2-MBP **A** and maleimide **B** for the synthesis of **C** through a [4 + 2] cycloaddition manifold. b) Light-driven reaction between 2-MBP **A** and 3-substituted coumarin **D** for the synthesis of **E** through a Michael addition manifold. c) Light-driven reaction between 2-MBPs **1** and coumarin (**2a**) or chromone (**3a**) for the synthesis of privileged tetracyclic scaffolds **4** or **5**.

In recent years synthetic photochemistry has become highly sophisticated [1]. The opportunity of using renewable energy sources to transform and functionalize organic molecules is receiving considerable interest from the scientific community [2]. Thus, innovative light-driven metal-free synthetic methods have been successfully developed [3]. More recently, the microfluidic photoreactor (MFP) technology has revealed to be a key technology applicable for diverse photochemical processes [4]. Microfluidic photoreactions allow an increased light penetration and surface-to-volume ratio together with a more uniform and effective irradiation of the reaction system [5], thus resulting in highly improved synthetic performances compared to the classical batch conditions. Recently, light-driven reactions of 2-methylbenzophenone (2-MBP) were reported to proceed smoothly under a MFP setup, furnishing highly diversified molecular scaffolds with enhanced yields and selectivities [6]. The chemistry is based on the ability of 2-MBP derivatives **A** of generating, upon light-irradiation, the highly reactive photoenol intermediate **A'** [7] and trapping of the latter by a competent electron-deficient reaction partner (Figure 1). The synthetic approach is not only restricted to electron-poor dienophiles such as maleimides **B** (see Figure 1a), but has also been implemented different reaction partners, allowing the light-promoted construction of biologically active natural products [8]. With this aim, electron-deficient chromophores, such as 3-coumarincarboxylates **D**, have been used as competent reaction partners of 2-MBPs **A**, furnishing 3-benzylated chromanones **E** through a Michael addition pathway (see Figure 1b) [6].

Prompted by the interest of developing novel light-driven microfluidic methods for the construction of biologically relevant molecular scaffolds, we investigated the reaction between MBP **1** and 3-unsubstituted coumarin (**2a**) and chromone (**3a**, Figure 1c). It was anticipated that the successful development of these photoreactions would generate valuable privileged scaffolds, namely, naphthochromenones **4** and benzoxanthenes **5**, through a diastereoselective light-driven [4 + 2] cycloaddition reaction. Interestingly, the tetracyclic scaffolds **4** and **5** are embodied in different biologically active molecules, with diverse pharmacological activities [9]. To the best of our knowledge, diastereoselective methods for the direct construction of naphthochromenone **4** are still missing. On the other hand, the reported construction of the benzoxanthene scaffold **5** relies on the use of harsh reaction conditions (e.g., 250 °C), leading to a mixture of regio- and diastereoisomers in moderate yields [10]. Hence, the development of an efficient synthetic method to access these privileged motifs still represents an open task in synthetic chemistry.

The method presented herein is suited for a broad range of coumarins **2** and chromones **3**, used as precursors for the direct generation of the tetracyclic scaffold **4** and **5**, with high synthetic performances (up to >98% yield) and complete diastereocontrol (>20:1). Additionally, manipulations of the naphthochromenone scaffold **4** give access to highly diversified molecular architectures, which are valuable intermediates in the synthesis of different biologically active molecules [11]. Noteworthy, the photoreactions presented herein do not proceed under conventional batch conditions, thus highlighting the importance of the MFP method enabling novel light-driven synthetic transformations.

## Results and Discussion

The reaction between 2-methylbenzophenone (**1a**) and coumarin (**2a**) was initially screened in a MFP of 1000 μL volume, using 1.5 equiv of **1a** and a residence time of 26.6 min (Table 1 and Table S3 in the Supporting Information File 1). Under these reaction conditions, product **4a** formed in 57% yield as a single detectable diastereoisomer with a production of 0.077 mmol·h^−1^ (entry 1 in Table 1). For comparison, in entry 2 of Table 1 are reported the reaction conditions previously described for the synthesis of **4a** [6]. Interestingly, a higher MFP volume resulted in a higher productivity: 0.077 mmol·h^−1^ vs 0.063 mmol·h^−1^ (entry 1 vs entry 2, Table 1). Reversing the reagents ratio, i.e., using a slight excess of coumarin (**2a**), turned out to be highly beneficial, giving the cyclized product **4a** in 77% yield (Table 1, entry 3). Notably, the optimal reaction conditions for the light-driven [4 + 2] cycloaddition were achieved within a 1000 μL MFP with a residence time set at 35 min forming product **4a** in quantitative yield, complete diastereocontrol and a productivity of 0.104 mmol·h^−1^ (Table 1, entry 4). On the contrary, when the same reaction was performed under batch conditions, the expected [4 + 2] cycloaddition product **4a** was only formed in trace amounts along with extensive product decomposition (Table 1, entry 5). The enhanced reactivity under the MFP compared to the batch setup (Table 1, entry 4 vs entry 5) is attributed to the more efficient illumination and the shorter irradiation time within the MFP [6], thus successfully preventing the light-promoted product decomposition [12]. In fact, the irradiation for 8 h of an authentic sample of **4a** resulted in the formation of a series of undefined decomposition products. Control experiments showed that in the absence of light irradiation, the cyclization product was not detected (Table 1, entry 6), confirming the photochemical nature of the present reaction.

**Table 1:** Light-driven reaction between 2-methylbenzophenone (**1a**) and coumarin (**2a**); selected optimization results.

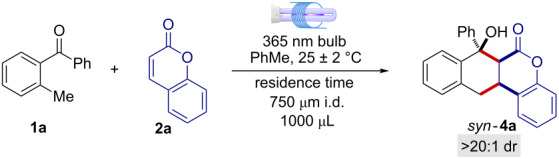					

entry^a^	residence time (min)	reactor volume (μL)	reagent ratio (**1a**:**2a**)	yield	production (mmol·h^−1^)

1	26.6	1000	1.5:1	57	0.077
2^b^	26.6	400	1.5:1	70^c^	0.063
3	26.6	1000	1:3	77	0.104
4	35	1000	1:5	>98%^c^	0.104
5^d,e^	480	1000	1:5	–	–
6^f^	35	1000	1:5	–	–

^a^Unless otherwise noted, reaction conditions were as follows: a degassed solution of **1a** and **2a** in toluene (0.06 M) was irradiated for the indicated time at 25 ± 2 °C (see Supporting Information File 1 for details). All yields refer to NMR yields using trimethoxybenzene as the internal standard. The dr was inferred by ^1^H NMR analysis on the crude reaction mixture and in all the cases resulted >20:1. ^b^Reaction conditions as described in [6]. ^c^Isolated yield. ^d^Reaction performed in batch. ^e^Extensive decomposition of both starting reagents **1a** and **2a** was observed by ^1^H NMR analysis of the crude reaction mixture. ^f^Reaction performed under MFP setup in the absence of light. i.d. = internal diameter.

**Figure 2:**
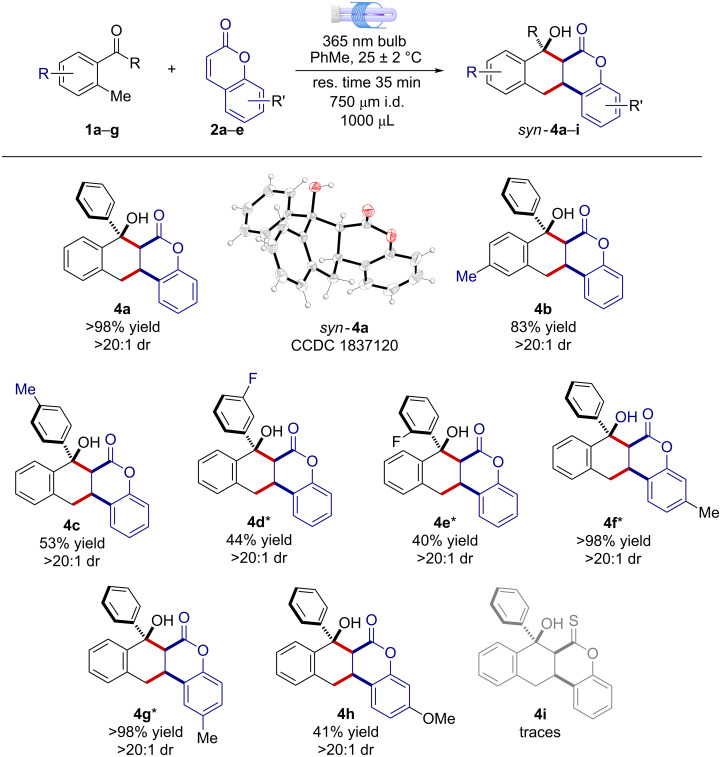
Generality and limits of the light-driven [4 + 2] cyclization reaction between 2-MBP **1a**–**g** and coumarins **2a**–**c** under MFP setup. *Residence time was 60 min.

With the optimal reaction conditions in hand we next explored the generality and limitations of the photochemical transformation (Figure 2). First, different substitutions on the 2-MBP scaffold were evaluated. Electron-donating substituents on both aromatic rings gave excellent results, furnishing the corresponding naphthochromenones **4b** and **4c** as single detectable diastereoisomers (>20:1 dr), with yields spanning from 53% to 83% and short residence times (35 min). On the contrary, electron-withdrawing substituents resulted in inferior synthetic performances. Compounds **4d** and **4e** were isolated in 44% and 40%, respectively within 60 min. The optimized reaction conditions were also amenable to diverse coumarin scaffolds. Six and 7-substituted coumarins furnished the corresponding cyclic products **4f**–**h** in moderate to excellent yields (41 to >98%) in a pure diastereoisomeric form. As a limitation of the present microfluidic photochemical method, thioxocoumarin **2e** showed poor reactivity under the titled reaction conditions, producing only traces of the expected sulfur-containing adduct **4i**. As a matter of fact, compound **2e** showed a high tendency to undergo a light-promoted [2 + 2] dimerization reaction, thus preventing the envisaged [4 + 2] cycloaddition pathway [13].

**Figure 3:**
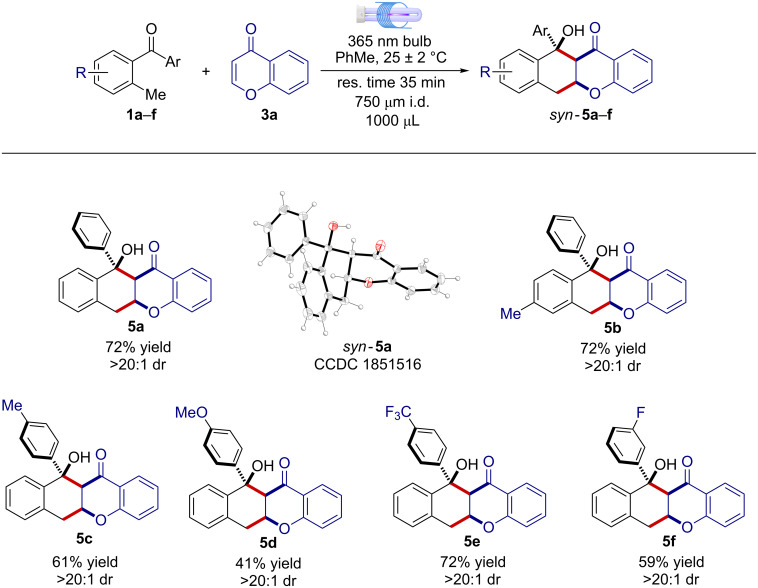
Generality and limits of the light-driven [4 + 2] cyclization reaction between 2-MBP **1a**–**f** and chromone (**3a**) under MFP setup.

Next, the chromone scaffold **3a**, which is a precursor of diverse classes of biologically active molecules [14], was evaluated under the developed MFP setup. Notably, the [4 + 2] cycloaddition product **5a** formed in 72% yield and >20:1 dr, without the need of further condition adjustments. The relative *syn* configuration within **5a** was inferred by 2D-NOESY experiments and confirmed by X-ray analysis of a suitable single crystal (Figure 3). Noteworthy, different 2-MBPs bearing electron-donating or electron-withdrawing groups underwent the light-driven [4 + 2] cycloaddition, affording the corresponding tetracyclic products **5b**–**f** with high dr and in good yields spanning from 41% to 72% (Figure 3).

**Scheme 1:**
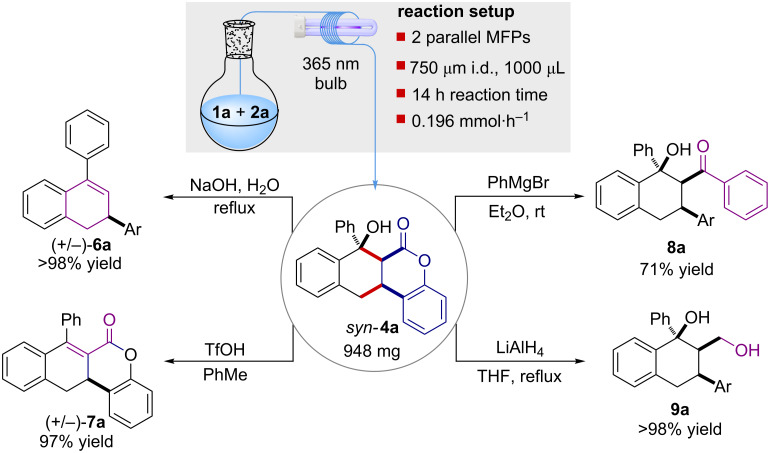
MFP parallel setup for higher scale production of **4a** (top) and different molecular scaffolds **6a–9a** accessible after simple manipulation (Ar = *o*-OH-C_6_H_4_).

In order to demonstrate the easy scalability of the present method (Scheme 1) two MFPs were used in a parallel setup producing 948 mg of **4a** after 14 h with an overall productivity rate of 0.196 mmol·h^−1^ [15]. Subsequently, a series of manipulations were conducted on product **4a**. Its treatment with a solution of sodium hydroxide in water promoted a quantitative lactone-opening/decarboxylation cascade sequence, yielding 2,4-dihydronaphthalene **6a** in quantitative yield without the need of chromatographic purification. Interestingly, scaffold **6a** is a valuable intermediate for the synthesis of biologically active natural compounds [16] and industrially relevant drugs [17] reminiscent of the bioactive tetralinolic pharmacophore core [18].

Acidic treatment of **4a** generated, after a simple extraction, the corresponding α,β-unsaturated compound **7a** in 97% yield. A further manipulation involved the treatment of **4a** with PhMgBr, converting the lactone moiety into the corresponding aromatic ketone. Product **8a** formed in 71% yield without diastereoisomeric loss. Finally, LiAlH_4_ reduction of **4a** furnished the bicyclic 1,3-diol **9a** in quantitative yield, again without the need of chromatographic purification. Noteworthy, compounds **6a**–**9a** embody different functionalities suitable for additional synthetic transformations.

## Conclusion

In conclusion we have developed an effective light-driven microfluidic method for the synthesis of valuable tetracyclic molecular architectures using commercially available precursors and a common 365 nm bulb. The reaction does not proceed under conventional batch conditions, highlighting the essential role of the developed MFP. A wide series of naphthochromenones and benzoxanthenes were synthesized in high yields and excellent diastereoselectivity. Finally, the large-scale production and subsequent manipulations of product **4a** demonstrated the high synthetic potential of the present MFP method, which is well-suited for the construction of diverse biologically active molecules.

## Supporting Information

File 1 Experimental procedures, characterization data for products **4a**–**h**, **5a**–**f** and **6a**–**9a**, NMR spectra, and CIF files for CCDC 1837120 and CCDC 1851516.
