# Supplementary material for: Microfluidic light-driven synthesis of tetracyclic molecular architectures
Source: Beilstein J Org Chem. 2018 Sep 17;14:2418–24. doi: 10.3762/bjoc.14.219 (PMC6176812; doi:10.3762/bjoc.14.219)
Supplement: File 1 — Experimental procedures, characterization data for products 4a–h, 5a–f and 6a–9a, NMR spectra, and CIF files for CCDC 1837120 and CCDC 1851516. [file Beilstein_J_Org_Chem-14-2418-s001.pdf]

Supporting Information

for

Microfluidic light-driven synthesis of tetracyclic molecular architectures

Javier Mateos, Nicholas Meneghini, Marcella Bonchio, Nadia Marino, Tommaso Carofiglio, Xavier Companyó* and Luca Dell'Amico*

Address: Dipartimento di Scienze Chimiche and ITM-CNR UoS of Padova, Università di Padova, Via Marzolo 1, 35131 Padova, Italy

Email: Xavier Companyó - xavier.companyo@unipd.it; Luca Dell'Amico - luca.dellamico@unipd.it

*Corresponding author

Experimental procedures, characterization data for products **4a**–**h**, **5a**–**f** and **6a**–**9a**, NMR spectra, and CIF files for CCDC 1837120 and CCDC 1851516

TABLE OF CONTENTS

A. General information S3

B. Supplementary tables S5

C. Microfluidic photo-reaction setup and batch photo-reaction setup S6

D. Light sources and emission spectra S10

E. General procedures for the reported reactions S11

F. Reaction optimisation.. S12

G. Absorption spectra analysis S14

H. Characterization data.. S18

I. Product manipulations S26

J. X-ray structure determination and refinement S30

K. References S35

L. NMR spectra.. S36

A. GENERAL INFORMATION

The continuous flow reactions were carried out using capillary reactors made with FEP tubing (0.75 mm i.d., 1.58 mm o.d.) and fitting connections purchased from BGB® (www.bgb-info.com). Reagents were pumped using Syrris Atlas pump (https://syrris.com/modules/atlas-syringe-pump-volumetric-dosing/). LEDs were purchased from Roithner LaserTechnik GmbH (model LED365-06Z 5.5 mW http://www.roithner-laser.com/index.html). 9W 365 nm bulb lamps were purchased from Amazon(https://www.amazon.it/gp/product/B073XL1T1J/ref=oh_aui_detailpage_o06_s00?ie=UTF8&psc=1).

NMR spectra were recorded on Bruker 400 Avance III HD equipped with a BBI-z grad probe head 5 mm and Bruker 500 Avance III equipped with a BBI-ATM-z grad probehead 5 mm. The chemical shifts (δ) for ^{1}H and ^{13}C NMR spectra are given in ppm relative to residual signals of the solvents ($CHCl_3$ @ 7.26 ppm ^{1}H NMR, 77.16 ppm ^{13}C NMR). Coupling constants are given in Hz. The following abbreviations are used to indicate the multiplicity: s, singlet; d, doublet; t, triplet; q, quartet; m, multiplet; bs, broad signal. NMR yields were calculated by using trichloroethylene as internal standard.

High resolution mass spectra (HRMS) were obtained using a Waters GCT gas chromatograph coupled with a time-of-flight mass spectrometer (GC/MS-TOF) with electron ionization (EI) or AB SCIEX MALDI-TOF 4800 Plus mass spectrometer (MALDI-TOF).

Chromatographic purification of products was accomplished using flash chromatography on silica gel (SiO_2, 0.04–0.063 mm) purchased from Machery-Nagel, with the indicated solvent system according to the standard techniques. Thin layer chromatography (TLC) analysis was performed on precoated Merck TLC plates (silica gel 60 GF254, 0.25 mm). Visualization of the developed chromatography was performed by checking UV absorbance (254 nm) as well as with aqueous ceric ammonium molybdate and potassium permanganate solutions. Organic solutions were concentrated under reduced pressure on a Büchi rotary evaporator.

The authors are grateful to the research support area at Department of Chemical Sciences (DiSC) of the University of Padova.

Determination of diastereomeric ratio: The diastereomeric ratio of products **4a**–**h**, **5a**–**f**, **6a**, **7a**, **8a** and **9a** was determined by ^{1}H NMR analysis of the crude reaction mixture through integration of diagnostic signals.

Materials: Commercial grade reagents and solvents were purchased at the highest commercial quality from Sigma Aldrich and used as received, unless otherwise stated. 2-methylbenzophenone (**1a**), (2,4-dimethylphenyl)(phenyl)methanone (**1b**), coumarin (**2a**), 6-methylcoumarin (**2b**), 7-methylcoumarin (**2c**), 7-methoxycoumarin (**2d**), as well as chromone (**3a**) were purchased from Sigma Aldrich and used as received. 2-MBP **1c**–**g**[1] as well as 2*H*-chromene-2-thione (**2e**)[2] were prepared according to literature procedures.

B. SUPPLEMENTARY TABLES

Table S1. Screened 2-methylbenzophenones **1**, under the developed MFP method.

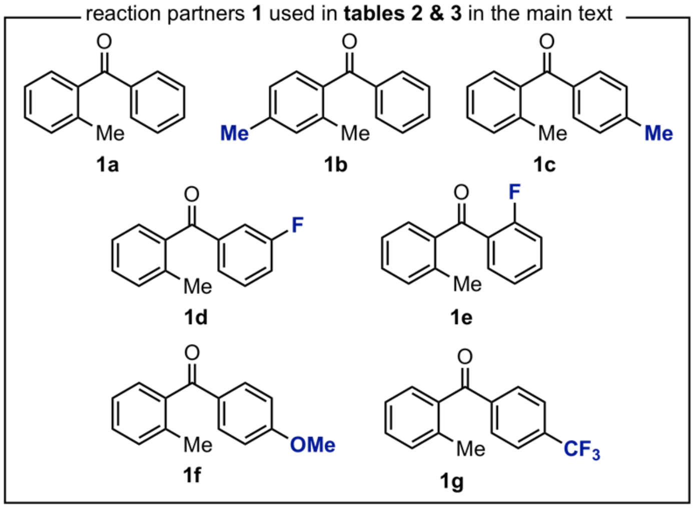

Table S2. Screened reaction partners **2** of 2-methylbenzophenone (**1a**), under the developed methodology.

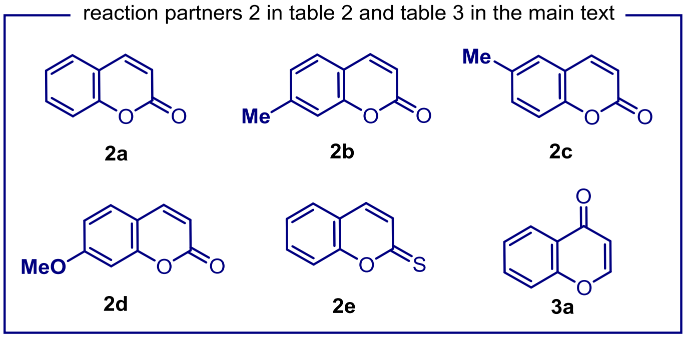

C. MICROFLUIDIC PHOTOREACTION SETUP AND BATCH REACTIONS SETUP

1. MICROFLUIDIC PHOTOREACTOR (MFP) SETUP

Figure S1 (below) shows a schematic representation of the microfluidic reactor employed in the present study for the photoreactions between 2-MBP **1** and coumarins **2a–e** or chromone (**3a**). In this setup, the solution containing **1a**–**g** (0.06 M in toluene) and the acceptor **2a**–**e** or **3a** (1.5 M in toluene) was firstly degassed by bubbling nitrogen for 15 min. Subsequently, the solution under a nitrogen atmosphere was introduced in continuous flow into the microphotoreactor via a double syringe pump (Syrris Atlas, see general information). The microfluidic reactor consists of a transparent TFE capillary (BGB®; internal diameter: 750 µm; inner volume: 1000 µL; microreactor tubing length: 226.5 cm); a 9 W 365 nm bulb lamp (Figure S2) equipped with a transparent glass-blown adaptor (Figure S3). Other microreactors used in this study consisted of 12 × 3 365 nm single LEDs (Figure S4). In both cases, aluminium foil was used to avoid undesired irradiation of the tubing. To maintain a stable reaction temperature, two fans were placed in close proximity to the reactor and the temperature was controlled by a thermometer (25 ± 2 °C).

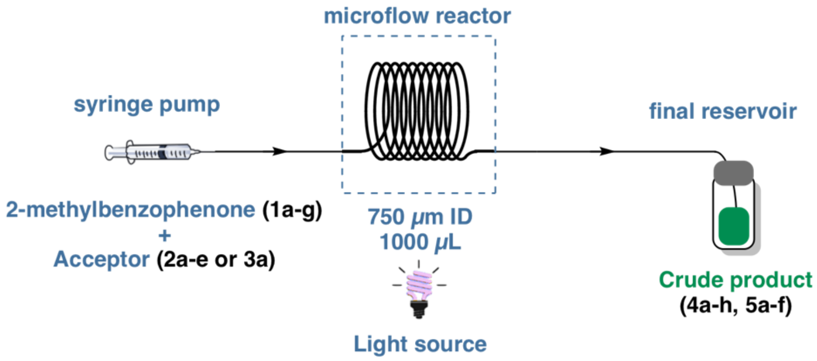

Figure S1. Schematic representation of the MFP setup. i.d.: internal diameter. Very similarly shown in reference 3.

A 0.1 mL aliquot of the product solution was introduced into an NMR tube, diluted with $CDCl_3$ and analysed by ^1H NMR in order to calculate the reaction conversion at different flow rates for each reaction. To determine the NMR yields, a 0.1 mL aliquot of the same solution was introduced into an NMR tube, using trimethoxybenzene as internal standard.

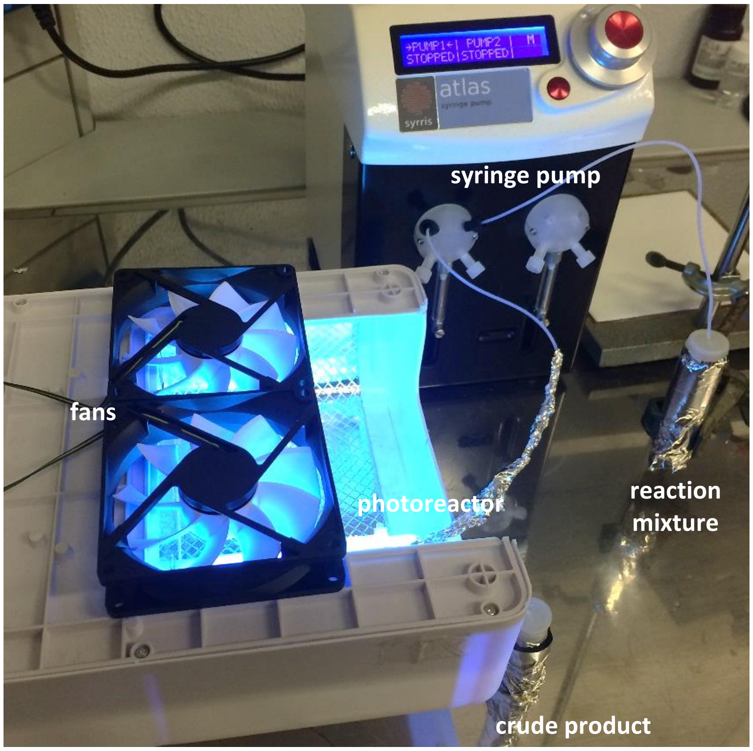

Figure S2. 9 W 365 nm bulb microfluidic photoreactor setup.

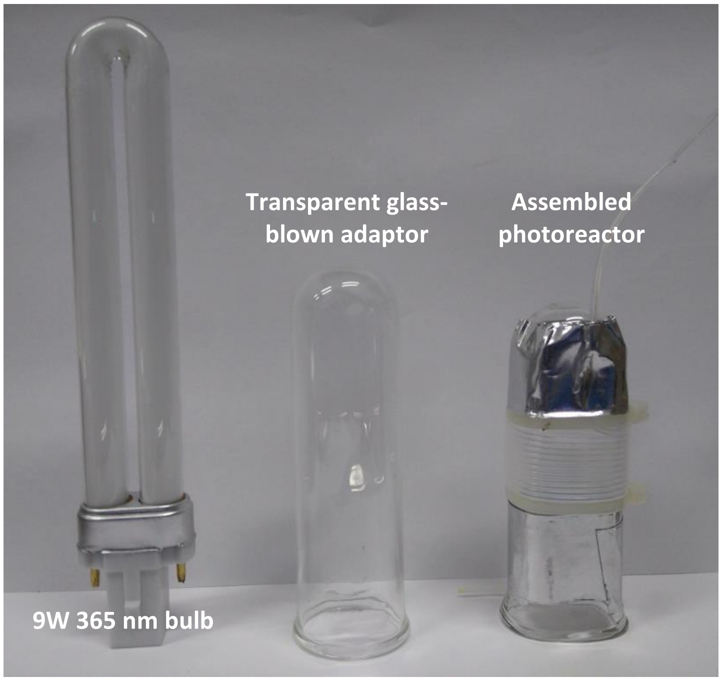

Figure S3. Left: 9 W 365 nm bulb used in this work. Middle: Transparent glass-blown adaptor used in this work. Right: Assembled microfluidic 400 µL photoreactor.

The photoreactor shown in Figure S3 consisted of a transparent glass-blown adaptor and a FEP tubing reactor wrapped around the support.

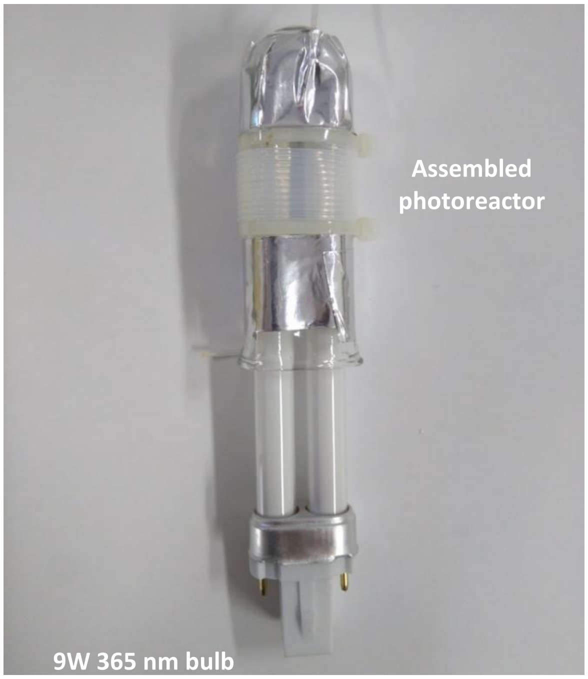

Figure S4. Final photoreactor setup.

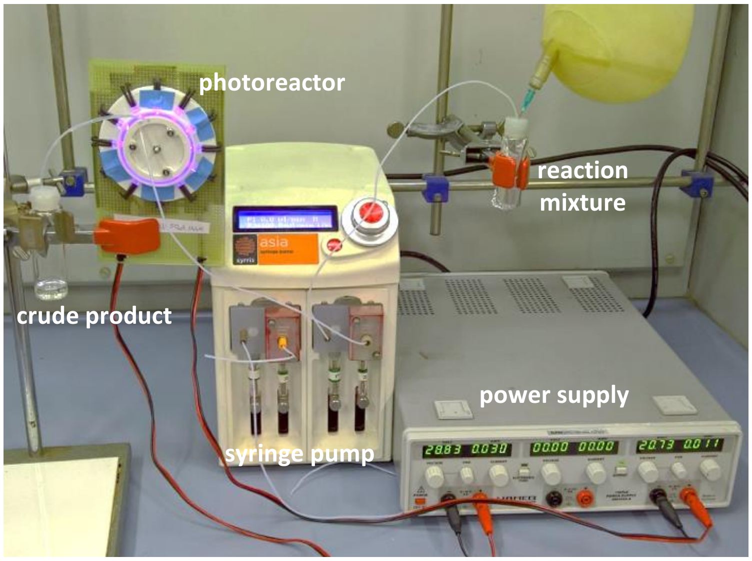

Figure S5. LED ring microfluidic photoreactor setup. Very similarly shown in reference 3.

The photoreactor shown in Figure S5 comprised a 3D-printed PLA support holding a ring of 12 LEDs pointing toward a FEP tubing reactor wrapped around a cylindrical support. The distance between the LEDs and the FEP tubing is 2 mm.

2. Batch photoreaction setup

Figure S6 (below) shows the general setup for the batch reactions. Two reaction vials containing the same degassed solution under N_2 atmosphere were placed in front of the 9 W 365 nm bulb (approximatively 1.5 cm distance). The reactions were stirred vigorously until full conversion was detected by ^1H NMR analysis of the crude reaction mixture. To maintain a stable reaction temperature two fans were placed in close proximity to the reaction vials (25 ± 2 °C) and the temperature was controlled by a thermometer.

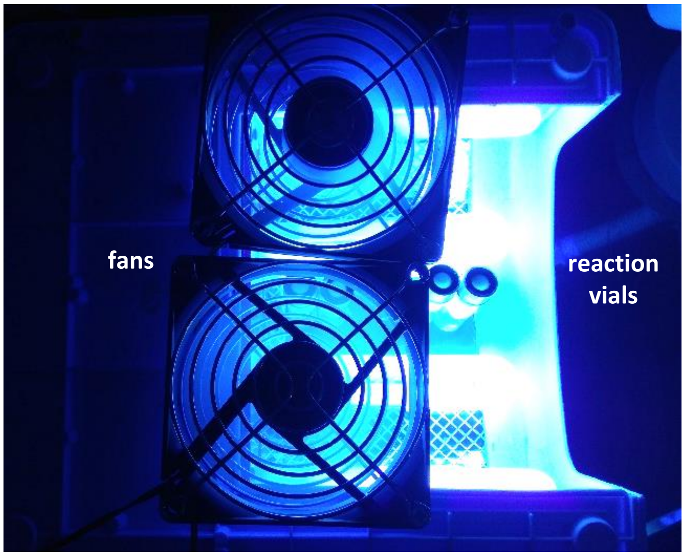

Figure S6. Setup of the batch photoreactions.

A 0.1 mL aliquot of the product solution was introduced into an NMR tube, diluted with $CDCl_3$ and analysed by ^1H NMR in order to calculate the reaction conversion at different times for each reaction. To determine the NMR yields, a 0.1 mL aliquot of the same solution was introduced into an NMR tube, using trimethoxybenzene as internal standard.

D. LIGHT SOURCES AND EMISSION SPECTRA

The following spectra were recorded by an AvaSpec ULS3648 high-resolution fiber-optic spectrometer which was placed at a fixed distance of 0.5 cm from the light source.

(More detailed information can be found at: https://www.avantes.com/products/spectrometers/starline/item/209-avaspec-uls3648-high-resolution-spectrometer).

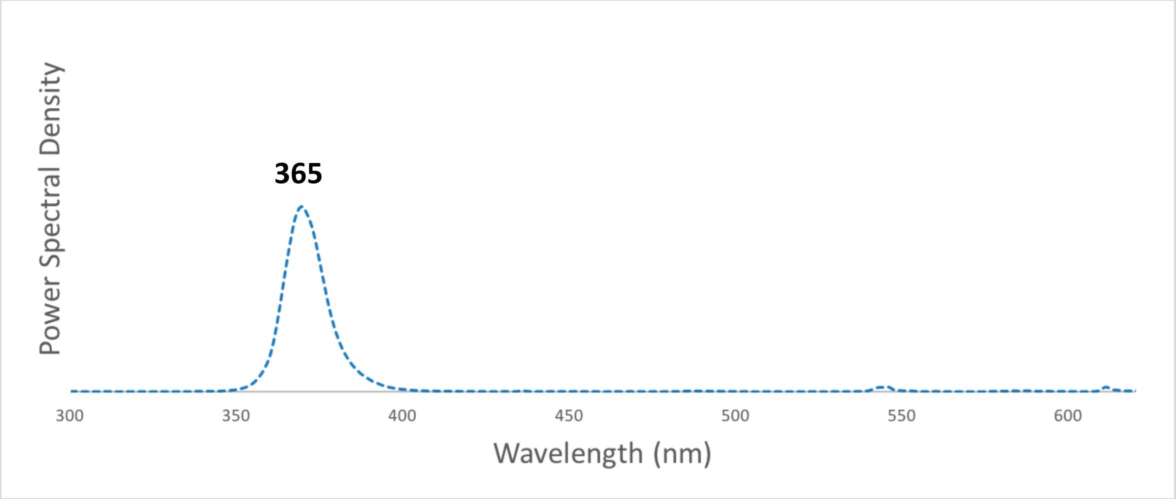

Figure S7. Emission spectra of the LEDs used in this study. Already reported in reference 3.

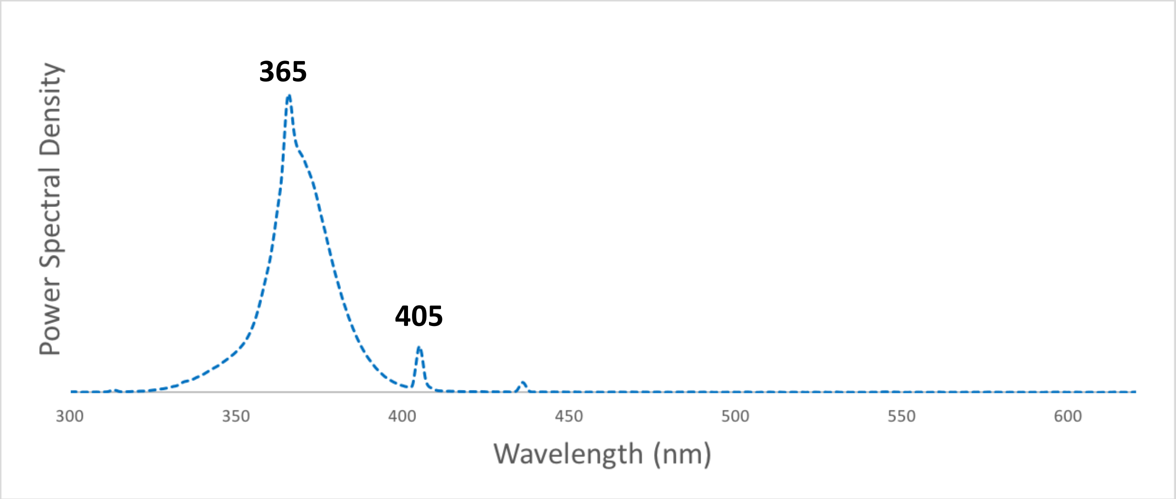

Figure S8. Emission spectra of the 9 W 365nm bulb light used in this study. Already reported in reference 3.

E. GENERAL PROCEDURES LIGHT-DRIVEN [4 + 2] CYCLOADDITION REACTIONS

1. GENERAL PROCEDURE FOR THE CONTINUOUS-FLOW REACTIONS

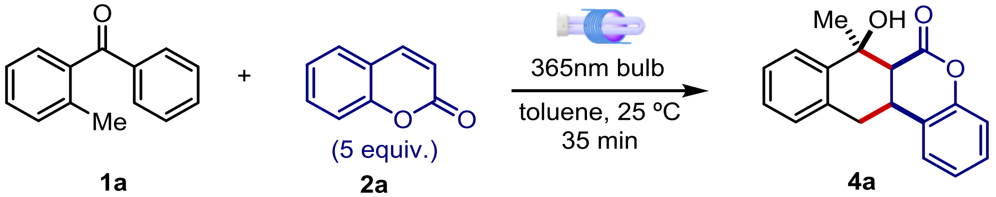

1a 2a 4a

Coumarin (**2a**, 219.2 mg, 5 equiv, 1.5 mmol) was introduced into a 12 mL vial under nitrogen atmosphere and dissolved in 5 mL of degassed toluene. Then, 2-methylbenzophenone (**1a**, 55 µL, 1 equiv, 0.3 mmol) was added in one portion and the solution was further bubbled with nitrogen for 5 min. The resultant solution was pumped into the MFP and irradiated by a 9 W 365 nm bulb with a residence time of 35 min. The product solution was collected into a 7 mL vial. Subsequently, the solvent was removed by rotary evaporation and the crude subjected to flash column chromatography on silica gel (9:1 hexane/EtOAc) yielding pure **4a** (white solid), as a single diastereoisomer in >98% yield (100.1 mg, 0.293 mmol).

F. REACTION OPTIMISATION

For the light-driven microfluidic [4 + 2] cycloaddition reaction between 2-MBP **1a** and coumarin (**2a**) investigated in the present work, different reaction conditions were tested in function of the light source and power, solvent, reagent concentration, flow rate, residence time and reactor volume.

Table S3. Exploratory studies of the microfluidic light-driven reaction between 2-methylbenzophenone (**1a**) and coumarin (**2a**).

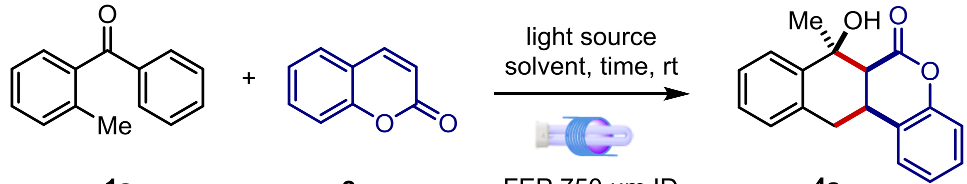

1a **2a** FEP 750 µm ID reactor volume **4a** >20:1 dr

entry	Conc. (M)	light source	Reactor volume (µL)	**1a**:**2a** ratio	residence time (min)	NMR yield[a] (%)[b]
**1**	0.1	365 nm bulb	400	1.5:1	26.6	70[c]
**2**	0.1	365 nm bulb	1000	1.5:1	26.6	57
**3**	0.1	365 nm bulb	250	1.5:1	26.6	48
**4**	0.1	365 nm bulb	400	3:1	26.6	47[b]
**5**	0.1	365 nm bulb	400	1:3	26.6	83[b]
**6**	0.06	365 nm bulb	400	1:3	4	12
**7**	0.06	365 nm bulb	400	1:3	8	32
**8**	0.06	365 nm bulb	400	1:3	12	37
**9**	0.06	12x365 nm LEDs	400	1:3	8	10
**10**	0.06	12x365 nm LEDs	400	1:3	26.6	40
**11**	0.06	365nm bulb adaptor	400	1:3	8	42
**12**	0.06	365nm bulb adaptor	400	1:3	12	55
**13**	0.06	365nm bulb adaptor	400	1:3	26.6	79
**14**	0.06	365nm bulb adaptor	1000	1:3	26.6	77

entry	Conc. (M)	light source	Reactor volume (μL)	**1a**:**2a** ratio	residence time (min)	NMR yield[a] (%)[b]
**15**	0.06	365nm bulb adaptor	400	1:5	26.6	>98[b]
**16**[d]	0.06	365 nm bulb	400	1:5	26.6	22
**17**[e]	0.06	365 nm bulb	400	1:5	26.6	10
**18**[f]	0.06	365 nm bulb	400	1:5	26.6	25
**19**	0.06	365 nm bulb	400	1:5	40	95
**20**	0.1	365 nm bulb adaptor	400	1:5	26.6	56
**21**	0.06	365 nm bulb	1000	1:5	26.6	80
**22**	0.06	365 nm bulb	1000	1:5	35	>98[b]
**23**	**0.06**	**365 nm bulb adaptor**	**1000**	**1:5**	**35**	**>98[b]**
**24**	0.06	batch[g]	1000	1:5	35	-
**25**	0.06	batch[g]	1000	1:5	60	-
**26**	0.06	batch[g]	1000	1:5	240	-
**27**	0.06	batch[g]	1000	1:5	480	<5%[h]

[a]Inferred by ^{1}H NMR analysis of the crude mixture. NMR yield calculated using trimethoxybenzene as internal standard [b]Isolated yield after flash chromatography. [c]Synthesised as already described in reference 3. [d]The reaction was performed using acetonitrile as solvent. [e]The reaction was performed using *o*-Cl_2Ph as solvent. [f]The reaction was performed using THF as solvent. [g]The reactions performed in batch were irradiated by a 9 W 365 nm bulb as reported in Figure S5, section C2 of this file. [h]Extensive decomposition was observed by ^{1}H NMR analysis.

G. ABSORPTION SPECTRA ANALYSIS

All the absorption spectra were recorded using an Agilent Cary 100 UV-Vis spectrophotometer. The spectra were recorded in toluene using the same concentrations as in the reaction conditions. Due to the high concentration of the solutions, short light path cuvettes (1 mm Hellma Quartz SUPRASIL®) were employed to avoid fast signal saturation.

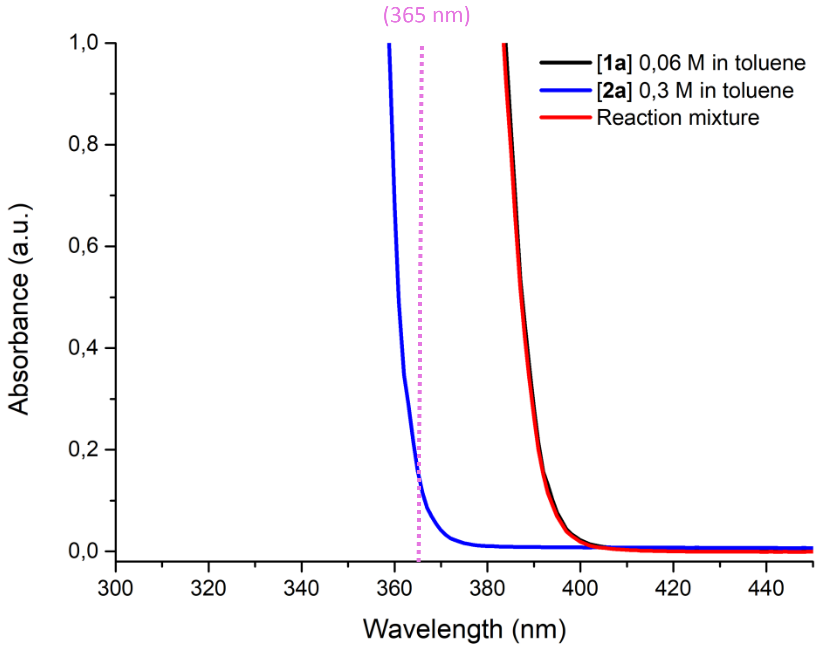

Figure S9. Comparison of the absorption spectra of 2-methylbenzophenone (**1a**, black line, [**1a**] = 0.06 M), coumarin (**2a**, blue line, [**2a**] = 0.3 M). The operative wavelength in the present work is shown as a dotted pink line

As shown in Figure S9 both 2-methylbenzophenone (**1a**) and coumarin (**2a**) are able to absorb the emitted light at 365 nm.

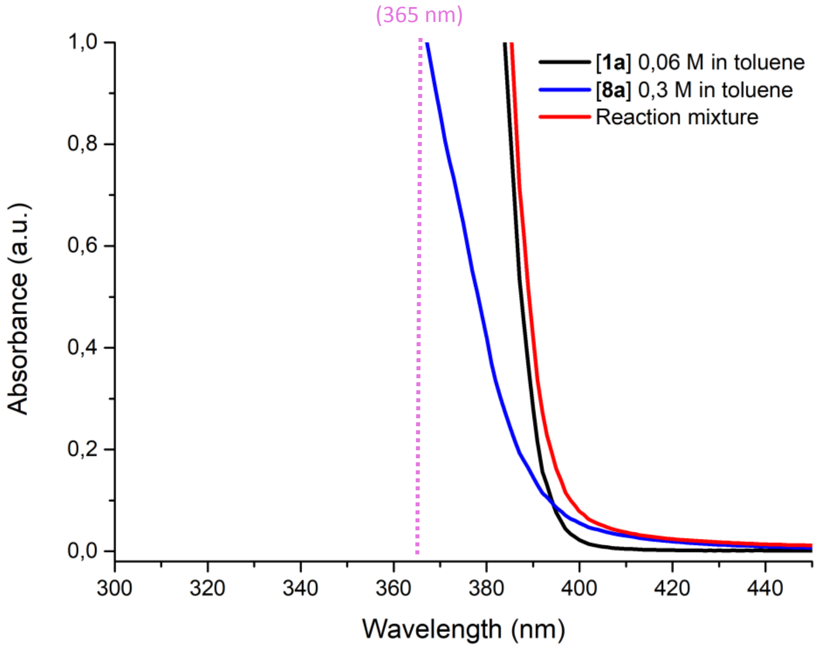

Figure S10. Comparison of the absorption spectra of 2-methylbenzophenone (**1a**, black line, [**1a**] = 0.06 M) and chromone (**3a**, blue line, [**3a**] = 0.3 M). The operative wavelength in the present work is shown as a dotted pink line.

As shown in Figure S10 both 2-methylbenzophenone (**1a**) and chromone (**8a**) are able to absorb the emitted light at 365 nm.

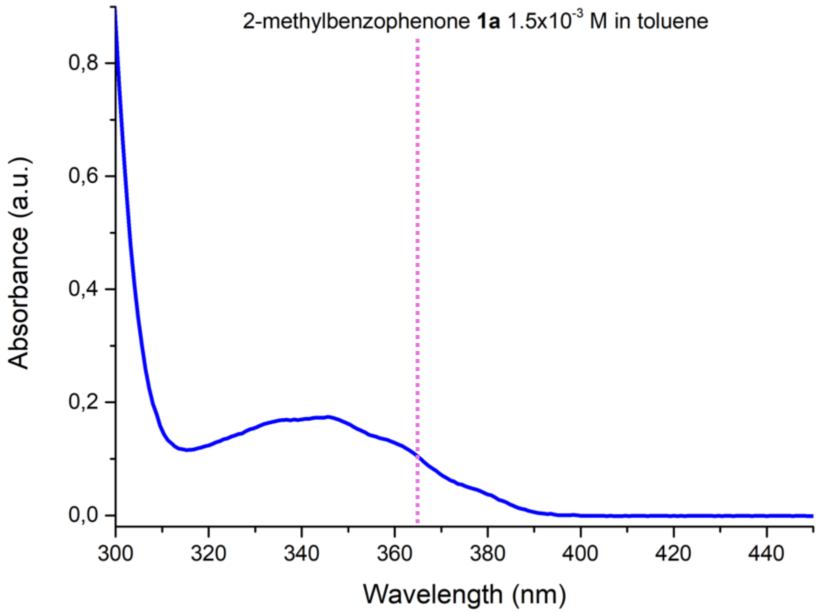

Figure S11. Absorption spectrum of 2-MBP **1a**. The spectrum was recorded for a degassed solution of toluene [**1a**] = 1.5 × 10^{-3}M into a 10 mm quartz cuvette. The operative wavelength in the present work (365 nm) is shown as a dotted pink line. Already reported in reference 3.

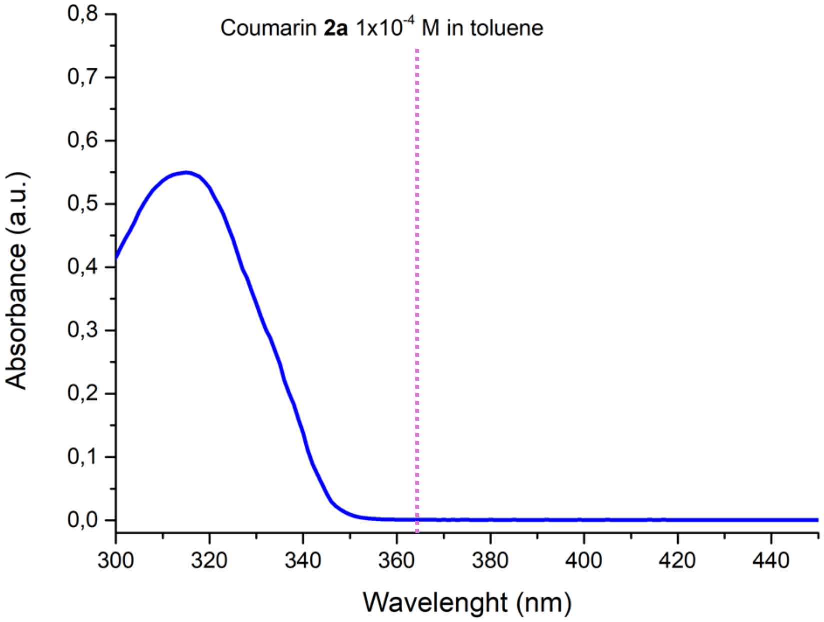

Figure S12. Absorption spectrum of coumarin (**2a**). The spectrum was recorded for a degassed solution of toluene [**2a**] = 1.0 × 10^{-4} M into a 10 mm quartz cuvette. The operative wavelength in the present work (365 nm) is shown as a dotted pink line.

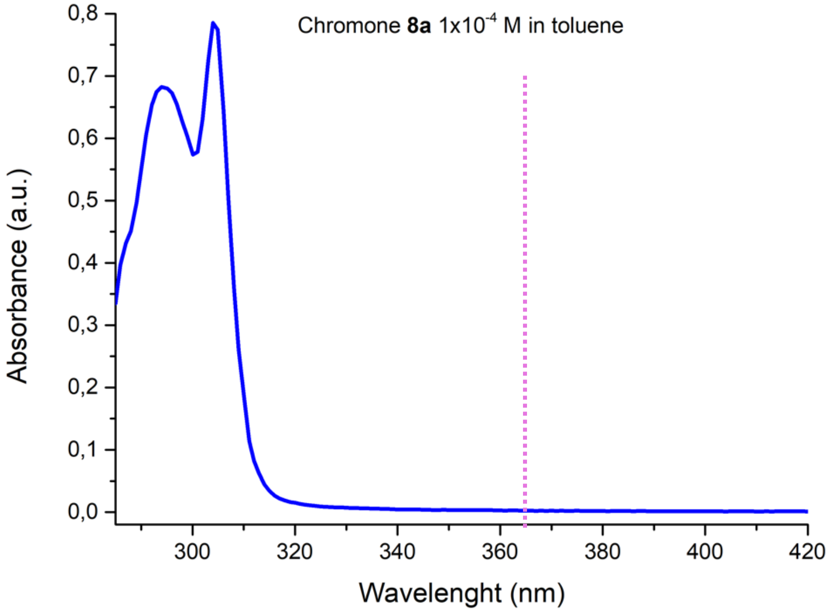

Figure S13. Absorption spectrum of chromone (**3a**). The spectrum was recorded for a degassed solution of toluene [**3a**] = 1.0 × 10^{-4} M into a 10 mm quartz cuvette. The operative wavelength in the present work (365 nm) is shown as a dotted pink line.

H. CHARACTERIZATION DATA

1. CHARACTERIZATION DATA FOR COMPOUNDS 4

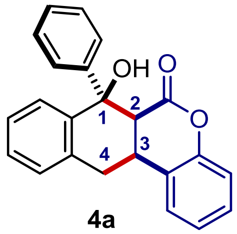

4a

7-Hydroxy-7-phenyl-6a,7,12,12a-tetrahydro-6*H*-naphtho[2,3-*c*]chromen-6-one (4a).

Synthesised following the continuous-flow procedure 1 described in section **E** using 1 equivalent of **1a** (55 µL, 0.3 mmol) and 5 equivalents of coumarin (**2a**, 219.2 mg, 1.5 mmol) in 5 mL of toluene with a residence time of 35 min**.** In order to calculate the isolated yield, 4 mL of the product solution of **4a** were collected and subjected to flash column chromatography on silica gel (9:1 Hexane/EtOAc) yielding pure **4a** (white solid), in >98% yield (82.0 mg, 0.24 mmol).

The obtained data matched with the previously reported in reference 3.

^{1}H-NMR (400 MHz, $CDCl_3$): 7.71 (d, *J* = 7.5 Hz, 1H, Ar), 7.37-7.27 (m, 6H, Ar), 7.19 (d, *J* = 7.7 Hz, 2H, Ar), 7.16–7.09 (m, 4H, Ar), 5.47 (br s, 1H, OH), 3.35 (d, *J* = 4.5 Hz, 1H, CH 2), 3.22 (dt, *J* = 12.8, 6.5, 2H, CH_2 4), 2.90 (dd, *J* = 19.4, 13.6, 1H, CH 3) ppm. **^{13}C-NMR (100 MHz, $CDCl_3$)**: δ 171.0 (COO), 150.5 (Cq Ar), 146.2 (Cq Ar), 139.4 (Cq Ar), 133.3 (Cq Ar), 128.7 (CH Ar), 128.0 (CH Ar), 128.0 (CH Ar x2), 127.7 (CH Ar), 127.7 (CH Ar), 127.7 (Cq Ar), 127.4 (CH Ar), 127.3 (CH Ar x2), 127.1 (CH Ar), 124.8 (CH Ar), 117.0 (CH Ar), 76.2 (Cq 1), 50.61 (CH), 33.4 (CH2), 33.2 (CH) ppm.

7-Hydroxy-10-methylphenyl-6a,7,12,12a-tetrahydro-6*H*-naphtho[2,3-*c*]chromen-6-one (4b).

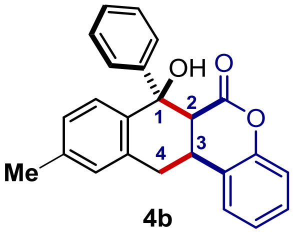

4b

Synthesised following the continuous-flow procedure 1 described in section **E** using 1 equivalent of **1b** (63.1 mg, 0.3 mmol) and 5 equivalents of coumarin (**2a**, 219.2 mg, 1.5 mmol) in 5 mL of toluene with a residence time of 35 min**.** In order to calculate the isolated yield, 4 mL of the product solution of **4b** were collected and subjected to flash column chromatography on silica gel (9:1 Hexane/EtOAc) yielding pure **4b** (white solid), in 83% yield, (70.9 mg, 0.199 mmol).

^{1}H-NMR (400 MHz, $CDCl_3$): 7.47 (d, *J* = 8.2 Hz, 1H, Ar), 7.23-7.14 (m, 4H, Ar), 7.10-6.98 (m, 6H, Ar), 6.84 (s, 1H, Ar), 5.30 (br s, 1H, OH), 3.20 (d, *J* = 4.4 Hz, 1H, C2H), 3.11-3.01 (m, 2H, C4H_2), 2.74 (dd, *J* = 15.4, 7.0, 1H, C3H), 2.27 (s, 3H, CH_3) ppm. **^{13}C-NMR (100 MHz, $CDCl_3$)**: δ 171.2 (COO), 150.7 (Cq Ar), 146.4 (Cq Ar), 137.9 (Cq Ar), 136.6 (Cq Ar), 133.3 (Cq Ar), 128.9 (CH Ar), 128.6 (CH Ar), 128.4 (CH Ar), 128.2 (CH Ar x2), 128.1 (CH Ar), 128.0 (Cq Ar), 127.9 (CH Ar), 127.5 (CH Ar x2), 127.3 (CH Ar), 124.9 (CH Ar), 117.1 (CH Ar), 76.3 (Cq 1),

50.9 (CH 2), 33.5 (CH2 4), 33.5 (CH 3), 21.3 (CH_3) ppm. **HRMS (MALDI)** calculated for $[C_{24}H_{20}O_3+Na]^+$: 379.1305, found: 379.1307.

7-Hydroxy-7-(*p*-tolyl)-6a,7,12,12a-tetrahydro-6*H*-naphtho[2,3-*c*]chromen-6-one (4c).

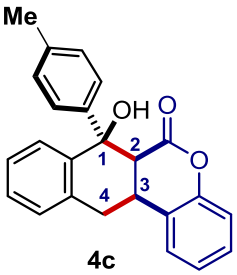

4c

Synthesised following the continuous-flow procedure 1 described in section **E** using 1 equivalent of **1c** (63.1 mg, 0.3 mmol) and 5 equivalents of coumarin (**2a**, 219.2 mg, 1.5 mmol) in 5 mL of toluene with a residence time of 35 min**.** In order to calculate the isolated yield, 4 mL of the product solution of **4c** were collected and subjected to flash column chromatography on silica gel (9:1 Hexane/EtOAc) yielding pure **4c** (white solid), in 53% yield (45.3 mg, 0.127 mmol).

^{1}H-NMR (400 MHz, $CDCl_3$): 7.64 (dd, *J*= 7.32, 1.78, 1H, Ar), 7.33-7.18 (m, 3H, Ar), 7.11-6.94 (m,8H, Ar), 5.37 (br s, 1H, OH), 3.25 (d, *J* = 3.44 Hz, 1H, CH), 3.23-3.14 (m, 2H, CH_2), 2.81 (td, *J* = 14.78, 5.92 Hz, 1H, CH), 2.28 (s, 3H, CH_3) ppm. **^{13}C-NMR (100 MHz, $CDCl_3$)**: δ 171.3 (COO), 150.7 (Cq Ar) 143.5 (Cq Ar), 139.8 (Cq Ar), 137.6 (Cq Ar), 133.4 (Cq Ar), 128.9 (CH Ar x3), 128.2 (CH Ar), 128.1 (CH Ar), 127.9 (Cq Ar), 127.9 (CH Ar), 127.5 (CH Ar), 127.4 (CH Ar x2), 127.3 (CH Ar), 124.9 (CH Ar), 117.1 (CH Ar), 76.2 (Cq 1), 50.8 (CH 2), 33.5 (CH_2 4), 33.4 (CH 3), 21.2 (CH_3) ppm. **HRMS (MALDI)** calculated for $[C_{24}H_{20}O_3+Na]^+$: 379.1305, found: 379.1305.

7-(3-Fluorophenyl)-7-hydroxy-6a,7,12,12a-tetrahydro-6*H*-naphtho[2,3-*c*]chromen-6-one (4d).

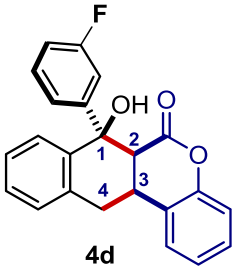

4d

Synthesised following the continuous-flow procedure 1 described in section **E** using 1 equivalent of **1d** (64.3 mg, 0.3 mmol) and 5 equivalents of coumarin (**2a**, 219.2 mg, 1.5 mmol) in 5 mL of toluene with a residence time of 60 min**.** In order to calculate the isolated yield, 4 mL of the product solution of **4d** were collected and subjected to flash column chromatography on silica gel (9:1 Hexane/EtOAc) yielding pure **4d** (white solid), in 44% yield (38 mg, 0.106 mmol).

^{1}H-NMR (400 MHz, $CDCl_3$): 7.59-7.57 (m, 1H, Ar), 7.26-7.14 (m, 4H, Ar), 7.05-7.01 (m, 4H, Ar), 6.90–6.80 (m, 3H, Ar), 5.39 (br s, 1H, OH), 3.20 (d, *J* = 4.9 Hz, 1H, CH 2), 3.14-3.07 (m, 2H, CH_2 4), 2.78 (dd, *J*= 19.9, 13.7 Hz, 1H, CH 3) ppm. **^{13}C-NMR (100 MHz, $CDCl_3$)**: δ 171.0 (COO), 150.6 (Cq Ar), 139.1 (Cq Ar), 133.4 (Cq Ar), 129.7 (Cq Ar), 129.7, 129.1 (CH Ar), 128.6, 128.1, 128.1, 127.8, 127.7, 127.4 (CH Ar), 125.1 (CH Ar),123.4, 123.3, 117.2, 115.1, 114.9, 76.1 (Cq 1), 50.7 (CH 2), 33.5 (CH_2 4), 33.4 (CH 3) ppm. **HRMS (MALDI)** calculated for $[C_{23}H_{17}FO_3+Na]^+$: 383.1054, found: 383.1051.

7-(2-Fluorophenyl)-7-hydroxy-6a,7,12,12a-tetrahydro-6*H*-naphtho[2,3-*c*]chromen-6-one (4e).

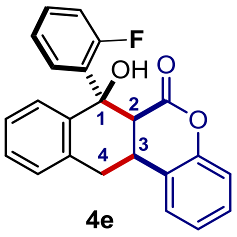

4e

Synthesised following the continuous-flow procedure 1 described in section **E** using 1 equivalent of **1e** (64.3 mg, 0.3 mmol) and 5 equivalents of coumarin (**2a**, 219.2 mg, 1.5 mmol) in 5 mL of toluene with a residence time of 60 min**.** In order to calculate the isolated yield, 4 mL of the product solution of **4e** were collected and subjected to flash column chromatography on silica gel (9:1 Hexane/EtOAc) yielding pure **4e** (white solid), in 40% yield (34.5 mg, 0.096 mmol).

^{1}H-NMR (400 MHz, $CDCl_3$): 7.64-7.62 (m, 1H, Ar), 7.27-7.19 (m, 4H, Ar), 7.06-7.01 (m, 5H, Ar), 6.90 (t, *J*= 8.4 Hz, 1H, Ar), 6.51 (t, *J*= 7.8 Hz, 1H, Ar), 4.88 (br s, 1H, OH), 3.76 (d, *J* = 4.8 Hz, 1H, CH 2), 3.14-3.06 (m, 2H, CH_2 4), 2.83 (dd, *J*= 19.7, 12.8 Hz, 1H, CH 3) ppm. **^{13}C-NMR (100 MHz, $CDCl_3$)**: δ 170.7 (COO), 161.7 (Cq Ar), 159.7 (Cq Ar), 150.9 (Cq Ar), 139.2, 133.4, 131.0, 130.3, 128.9, 128.5, 128.3, 128.2, 127.6, 127.5, 127.1, 125.0, 123.7, 117.2, 117.1, 75.6 (Cq 1), 48.1 (CH 2), 33.6 (CH_2 4), 33.1 (CH 3) ppm **HRMS (MALDI)** calculated for $[C_{23}H_{17}FO_3+Na]^+$: 383.1054, found: 383.1052.

7-Hydroxy-3-methyl-7-phenyl-6a,7,12,12a-tetrahydro-6*H*-naphtho[2,3-*c*]chromenone (4f).

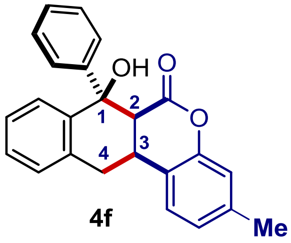

4f

Synthesised following the continuous-flow procedure 1 described in section **E** using 1 equivalent of 2-MBP **1a** (55 µL, 0.3 mmol) and 5 equivalents of 7-methylcoumarin (**2b**, 240.3 mg, 1.5 mmol) in 5 mL of toluene with a residence time of 60 min**.** In order to calculate the isolated yield, 4 mL of the product solution of **4f** was collected and subjected to flash column chromatography on silica gel (9:1 Hexane/EtOAc) yielding pure **4f** (white solid), in >98% yield (85.4 mg, 0.240 mmol).

^{1}H-NMR (400 MHz, $CDCl_3$): 7.65-7.41 (m, 1H, Ar), 7.25-7.13 (m, 5H, Ar), 7.06 (dd, *J* = 7.7, 2.0 Hz, 2H, Ar), 7.02 (dd, *J* = 6.5, 2.2 Hz, 1H, Ar), 6.97 (dd, *J* = 8.3, 1.7 Hz, 1H, Ar), 6.88 (d, *J* = 8.3 Hz, 1H, Ar), 6.77 (d, *J* = 1.8 Hz, 1H, Ar), 5.39 (br s, 1H, OH), 3.19 (d, *J* = 3.2 Hz, 1H, CH 2), 3.12-3.00 (m, 2H, CH_2 4), 2.75 (td, *J* = 15.8, 7.1 Hz, 1H, CH 3), 2.18 (s, 3H, CH_3) ppm. **^{13}C-NMR (100 MHz, $CDCl_3$)**: δ 171.1 (COO), 148.2 (Cq Ar) 146.1 (Cq Ar), 139.3 (Cq Ar), 134.4 (Cq Ar), 133.2 (Cq Ar), 128.9 (CH Ar), 127.9 (CH Ar x4), 127.6 (Cq Ar), 127.5 (CH Ar), 127.4 (CH Ar), 127.2 (CH Ar), 127.2 (CH Ar x3), 116.5 (CH Ar), 76.1 (Cq 1), 50.5 (CH 2), 33.3 (CH_2 4), 33.0 (CH 3), 20.5 (CH_3) ppm. **HRMS (MALDI)** calculated for $[C_{24}H_{20}O_3+Na]^+$: 379.1305, found: 379.1302.

7-Hydroxy-2-methyl-7-phenyl-6a,7,12,12a-tetrahydro-6*H*-naphtho[2,3-*c*]chromen-6-one (4g).

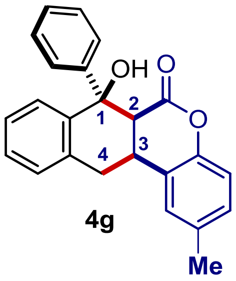

4g

Synthesised following the continuous-flow procedure 1 described in section **E** using 1 equivalent of 2-MBP **1a** (55 µL, 0.3 mmol) and 5 equivalents of 6-methylcoumarin (**2c**, 240.3 mg, 1.5 mmol) in 5 mL of toluene with a residence time of 60 min. In order to calculate the isolated yield, 4 mL of the product solution of **4g** was collected and subjected to flash column chromatography on silica gel (9:1 Hexane/EtOAc) yielding pure **4g** (white solid), in >98% yield (85.2 mg, 0.240 mmol).

^{1}H-NMR (400 MHz, CDCl$_3$): 7.59 (dd, *J*= 7.3, 2.0, 1H, Ar), 7.27-7.13 (m, 5H, Ar), 7.06 (dd, *J* = 7.7, 2.0 Hz, 2H, Ar), 7.02 (dd, *J* = 6.6, 2.05 Hz, 1H, Ar), 6.89-6.77 (m, 3H, Ar), 5.37 (br s, 1H, OH), 3.19 (d, *J* = 4,7 Hz, 1H, CH _2_), 3.11-2.98 (m, 2H, CH$_2$ _4_), 2.74 (td, *J* = 19.7, 13.8 Hz, 1H, CH _3_), 2.25 (s, 3H, CH$_3$) ppm. **^{13}C-NMR (100 MHz, CDCl$_3$)**: δ 171.8 (COO), 150.9 (Cq Ar) 146.8 (Cq Ar), 140.0 (Cq Ar), 139.6 (Cq Ar), 133.9 (Cq Ar), 128.5 (CH Ar x4), 128.2 (CH Ar), 128.2 (CH Ar), 127.8 (CH Ar x3), 127.3 (CH Ar), 125.9 (CH Ar), 125.1 (Cq Ar), 117.9 (CH Ar), 76.7 (Cq _1_), 51.3 (CH _2_), 34.0 (CH$_2$ _4_), 33.4 (CH _3_), 21.6 (CH$_3$) ppm. **HRMS (MALDI)** [$C_{24}H_{20}O_3$+Na]$^+$: 379.1305, found: 379.1307.

7-Hydroxy-3-methoxy-7-phenyl-6a,7,12,12a-tetrahydro-6*H*-naphtho[2,3-*c*]chromen-6-one (4h).

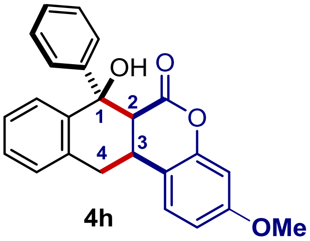

4h

Synthesised following the continuous-flow procedure 1 described in section **E** using 1 equivalent of **1a** (27.5 µL, 0.15 mmol) and 5 equivalents of coumarin (**2d**, 132 mg, 0.75 mmol) in 5 mL of toluene with a residence time of 35 min**.** In order to calculate the isolated yield, 4 mL of the product solution of **4h** were collected and subjected to flash column chromatography on silica gel (9:1 Hexane/EtOAc) yielding pure **4h** (white solid), in 41% yield (19.5 mg, 0.049 mmol).

^{1}H-NMR (400 MHz, CDCl$_3$): 7.59 (d, *J* = 7.5 Hz, 1H, Ar), 7.25-7.18 (m, 5H, Ar), 7.08 (dd, *J* = 7.8, 2.4 Hz, 2H, Ar), 7.03 (dd, *J* = 7.7, 2.4 Hz, 1H, Ar), 6.88 (d, *J* = 7.5, 1H, Ar), 6.57-6.52 (m, 2H, Ar), 5.33 (br s, 1H, OH), 3.71 (s, 3H, OMe), 3.21 (d, *J* = 4.0 Hz, 1H, CH _2_), 3.12-3.02 (m, 2H, CH$_2$ _4_), 2.74 (td, *J* = 19.7, 13.8 Hz, 1H, CH _3_) ppm. **^{13}C-NMR (100 MHz, CDCl$_3$)**: δ 171.2 (COO), 160.1 (Cq Ar) 151.4 (Cq Ar), 146.4 (Cq Ar), 139.6 (Cq Ar), 133.6 (Cq Ar), 128.2 (CH Ar x4), 127.9 (CH Ar), 127.9 (CH Ar), 127.8 (CH Ar), 127.5 (CH Ar x3), 119.9 (Cq Ar), 110.7 (CH Ar), 102.9 (CH Ar), 76.4 (Cq _1_), 55.8 (OMe), 51.1 (CH _2_), 33.9 (CH$_2$ _4_), 32.7 (CH _3_) ppm. **HRMS (MALDI)** [$C_{24}H_{20}O_4$+Na]$^+$: 395.1254, found: 395.1257.

2. CHARACTERIZATION DATA FOR COMPOUNDS 5

11-Hydroxy-11-phenyl-5a,6,11,11a-tetrahydro-12*H*-benzo[*b*]xanthen-12-one (5a).

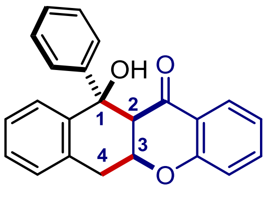

5a

Synthesised following the continuous-flow procedure 1 described in section **E** using 1 equivalent of **1a** (55 µL, 0.3 mmol) and 5 equivalents of chromone (**3a**, 219.2 mg, 1.5 mmol) in 5 mL of toluene with a residence time of 35 min**.** In order to calculate the isolated yield, 4 mL of the product solution of **5a** were collected and subjected to flash column chromatography on silica gel (95:5 Hexane/EtOAc) yielding pure **5a** (white solid), in 72% yield (76.6 mg, 0.168 mmol).

^{1}H-NMR (400 MHz, CDCl$_3$): 7.74 (dd, *J* = 7.5, 1.9 Hz, 1H, Ar), 7.53-7.49 (m, 1H, Ar), 7.40 (ddd, *J* = 15.6, 7.4, 2.0 Hz, 1H, Ar), 7.24–7.15 (m, 5H, Ar), 7.07-7.00 (m, 3H, Ar), 6.91 (t, *J*= 7.4 Hz, 1H, Ar), 6.83 (d, *J*= 8.4 Hz, 1H, Ar), 5.67 (br s, 1H, OH), 4.71 (ddd, *J* = 9.5, 7.1, 5.1 Hz, 1H, CH *3*), 3.57 (d, *J*= 5.8 Hz, 1H, CH *2*), 3.22 (dd, *J*= 17.5, 9.5 Hz, 1H, CH$_2$ *4a*), 3.22 (dd, *J*= 17.5, 7.0 Hz, 1H, CH$_2$ *4b*) ppm. **^{13}C-NMR (100 MHz, CDCl$_3$)**: δ 196.0 (C=O), 159.7 (Cq Ar), 146.4 (Cq Ar), 140.5 (Cq Ar), 137.3 (CH Ar), 132.4 (Cq Ar), 128.8 (CH Ar), 128.7 (CH Ar x2), 128.6 (CH Ar x2), 128.2 (CH Ar), 127.9 (CH Ar), 127.5 (CH Ar x2), 127.3 (CH Ar), 121.8 (CH Ar), 121.3 (Cq Ar), 118.8 (CH Ar), 78.3 (Cq *1*), 74.2 (CH *2*), 55.4 (CH *3*), 30.8 (CH$_2$ *4*) ppm. **HRMS (MALDI)** calculated for $[C_{23}H_{18}O_3+Na]^+$: 365.1154, found: 365.1158.

11-Hydroxy-8-methyl-11-phenyl-5a,6,11,11a-tetrahydro-12*H*-benzo[*b*]xanthen-12-one (5b).

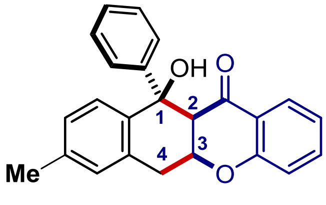

5b

Synthesised following the continuous-flow procedure 1 described in section **E** using 1 equivalent of **1b** (63.1 mg, 0.3 mmol) and 5 equivalents of chromone (**3a**, 219.2 mg, 1.5 mmol) in 5 mL of toluene with a residence time of 35 min**.** In order to calculate the isolated yield, 4 mL of the product solution of **5b** were collected and subjected to flash column chromatography on silica gel (9:1 Hexane/EtOAc) yielding pure **5b** (white solid), in 72% yield (61.6 mg, 0.173 mmol).

^{1}H-NMR (400 MHz, CDCl$_3$): 7.73 (d, *J* = 7.5 Hz, 1H, Ar), 7.43-7.36 (m, 2H, Ar), 7.26-7.13 (m, 3H, Ar), 7.06 (d, *J*=7.5 Hz, 2H, Ar), 7.00 (d, *J*= 7.8 Hz, 1H, Ar), 6.90 (t, *J*= 7.4 Hz, 1H, Ar), 6.83-6.81 (m, 2H, Ar), 5.53 (br s, 1H, OH), 4.77 (ddd, *J* = 9.5, 7.1, 5.1 Hz, 1H, CH *3*), 3.53 (d, *J*= 5.8 Hz, 1H, CH *2*), 3.17 (dd, *J*= 17.5, 9.5 Hz, 1H, CH$_2$ *4a*), 3.09 (dd, *J*= 17.5, 7.0 Hz, 1H, CH$_2$ *4b*), 2.24 (s, 3H, CH$_3$) ppm. **^{13}C-NMR (100 MHz, CDCl$_3$)**: δ 195.5 (C=O), 159.3 (Cq Ar), 146.2 (Cq Ar), 138.0 (Cq Ar), 137.3 (Cq Ar), 136.9 (CH Ar), 131.8 (Cq Ar), 128.8 (CH Ar), 128.6 (CH Ar), 128.2 (CH Ar), 128.1 (CH Ar x2), 127.7 (CH Ar), 127.2 (CH Ar x2), 127.0 (CH Ar), 121.4

(CH Ar), 121.0 (Cq Ar), 118.3 (CH Ar), 77.7 (Cq _1_), 73.2 (CH _2_), 55.1 (CH _3_), 30.4 (CH_2 _4_), 21.2 (CH_3) ppm. **HRMS (MALDI)** calculated for $[C_{24}H_{20}O_3+Na]^+$: 365.1310, found: 365.1315.

11-Hydroxy-11-(*p*-tolyl)phenyl-5a,6,11,11a-tetrahydro-12*H*-benzo[*b*]xanthen-12-one (5c).

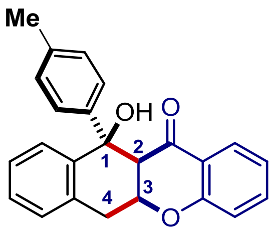

5c

Synthesised following the continuous-flow procedure 1 described in section **E** using 1 equivalent of **1c** (63.1 mg, 0.3 mmol) and 5 equivalents of chromone (**3a**, 219.2 mg, 1.5 mmol) in 5 mL of toluene with a residence time of 35 min**.** In order to calculate the isolated yield, 4 mL of the product solution of **5c** were collected and subjected to flash column chromatography on silica gel (9:1 Hexane/EtOAc) yielding pure **5c** (white solid), in 61% yield (52.2 mg, 0.146 mmol).

^{1}H-NMR (400 MHz, $CDCl_3$): 7.73 (dd, *J* = 7.2, 2.1 Hz, 1H, Ar), 7.53-7.49 (m, 1H, Ar), 7.40 (ddd, *J* = 15.6, 7.4, 2.0 Hz, 1H, Ar), 7.20–7.14 (m, 2H, Ar), 7.03-6.98 (m, 3H, Ar), 6.94-6.88 (m, *3*H, Ar), 6.83 (d, *J*= 8.3 Hz, 1H, Ar), 5.62 (br s, 1H, OH), 4.80 (ddd, *J* = 9.5, 7.1, 5.1 Hz, 1H, CH _3_), 3.55 (d, *J*= 5.8 Hz, 1H, CH _2_), 3.21 (dd, *J*= 17.5, 9.5 Hz, 1H, CH_2 _4a_), 3.13 (dd, *J*= 17.5, 7.0 Hz, 1H, CH_2 _4b_), 2.24 (s, 3H, CH_3) ppm. **^{13}C-NMR (100 MHz, $CDCl_3$)**: δ 195.6 (C=O), 159.3 (Cq Ar), 143.2 (Cq Ar), 140.4 (Cq Ar), 137.4 (Cq Ar), 136.9 (CH Ar), 132.0 (Cq Ar), 128.9 (CH Ar x2), 128.3 (CH Ar), 128.3 (CH Ar), 128.2 (CH Ar), 127.5 (CH Ar), 127.0 (CH Ar x2), 126.9 (CH Ar), 121.4 (CH Ar), 120.9 (Cq Ar), 118.4 (CH Ar), 77.7 (Cq _1_), 73.8 (CH _2_), 55.1 (CH _3_), 30.4 (CH_2 _4_), 21.1 (CH_3) ppm. **HRMS (MALDI)** calculated for $[C_{24}H_{20}O_3+Na]^+$: 365.1315, found: 365.1319.

11-Hydroxy-11-(4-methoxy)phenyl-5a,6,11,11a-tetrahydro-12*H*-benzo[*b*]xanthen-12-one (5d)

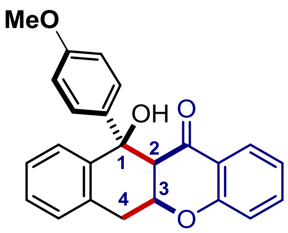

5d

Synthesised following the continuous-flow procedure 1 described in section **E** using 1 equivalent of **1f** (67.9 mg, 0.3 mmol) and 5 equivalents of chromone (**3a**, 219.2 mg, 1.5 mmol) in 5 mL of toluene with a residence time of 35 min**.** In order to calculate the isolated yield, 4 mL of the product solution of **5d** were collected and subjected to flash column chromatography on silica gel (95:5 Hexane/EtOAc) yielding pure **5d** (white solid), in 41% yield (37.1 mg, 0.099 mmol).

^{1}H-NMR (400 MHz, $CDCl_3$): 7.73 (dd, *J* = 7.5, 1.9 Hz, 1H, Ar), 7.55-7.53 (m, 1H, Ar), 7.42 (ddd, *J* = 15.6, 7.4, 2.0 Hz, 1H, Ar), 7.22–7.15 (m, 2H, Ar), 7.04-7.00 (m, 1H, Ar), 7.97-6.88 (m, 3H, Ar), 6.84 (d, *J*= 7.4 Hz, 1H, Ar), 6.73 (d, *J*= 8.4 Hz, 1H, Ar), 5.67 (br s, 1H, OH), 4.80 (ddd, *J* = 9.5, 7.1, 5.1 Hz, 1H, CH _3_), 3.71 (s, 3H, OMe), 3.57 (d, *J*= 5.8 Hz, 1H, CH _2_), 3.20 (dd, *J*= 17.5, 9.5 Hz, 1H, CH_2 _4a_), 3.12 (dd, *J*= 17.5, 7.0 Hz, 1H, CH_2 _4b_) ppm. **^{13}C-NMR (100**

MHz, $CDCl_3$): δ 195.7 (C=O), 159.3 (Cq Ar), 159.1 (Cq Ar), 140.3 (Cq Ar), 138.4 (Cq Ar), 137.0 (CH Ar), 132.0 (Cq Ar), 128.4 (CH Ar x3), 128.3 (CH Ar), 128.2 (CH Ar x3), 121.4 (CH Ar), 120.9 (Cq Ar), 118.4 (CH Ar), 113.5 (CH Ar x2), 77.7 (Cq *1*), 73.8 (CH *2*), 55.4 (OMe), 55.42(CH *3*), 30.3 (CH_2 *4*) ppm. **HRMS (MALDI)** calculated for $[C_{24}H_{20}O_4+Na]^+$: 395.1259, found: 395.1262.

11-Hydroxy-11-(4-trifluoromethyl)phenyl-5a,6,11,11a-tetrahydrobenzo[*b*]xanthen-12-one (5e).

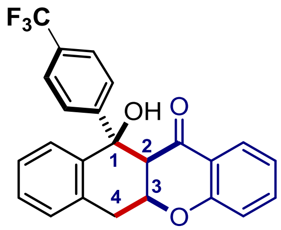

5e

Synthesised following the continuous-flow procedure 1 described in section **E** using 1 equivalent of **1g** (79.3 mg, 0.3 mmol) and 5 equivalents of chromone (**3a**, 219.2 mg, 1.5 mmol) in 5 mL of toluene with a residence time of 35 min**.** In order to calculate the isolated yield, 4 mL of the product solution of **5e** were collected and subjected to flash column chromatography on silica gel (95:5 Hexane/EtOAc) yielding pure **5e** (white solid), in 72% yield (70.9 mg, 0.173 mmol).

^{1}H-NMR (400 MHz, $CDCl_3$): 7.74 (dd, *J* = 7.5, 1.9 Hz, 1H, Ar), 7.50-7.36 (m, 4H, Ar), 7.24–7.14 (m, 4H, Ar), 7.09-7.04 (m, 1H, Ar), 6.92 (t, *J*= 7.5 Hz, 1H, Ar), 6.83 (d, *J*= 8.4 Hz, 1H, Ar), 5.45 (br s, 1H, OH), 4.77 (ddd, *J* = 9.5, 7.1, 5.1 Hz, 1H, CH *3*), 3.46 (d, *J*= 6.0 Hz, 1H, CH *2*), 3.26 (dd, *J*= 17.5, 9.5 Hz, 1H, CH_2 *4a*), 3.17 (dd, *J*= 17.5, 7.0 Hz, 1H, CH_2 *4b*) ppm. **^{13}C-NMR (100 MHz, $CDCl_3$)**: δ 194.8 (C=O), 159.4 (Cq Ar), 150.1 (Cq Ar), 139.6 (Cq Ar), 137.0 (CH Ar), 131.2 (Cq Ar), 128.7 (CH Ar), 128.7 (CH Ar), 128.4 (CH Ar), 127.8 (CH Ar), 127.6 (CH Ar x4), 127.0 (CH Ar), 125.3 (CF_3), 121.8 (CH Ar), 121.0 (Cq Ar), 118.4 (CH Ar), 77.3 (Cq *1*), 73.7 (CH *2*), 54.8 (CH *3*), 30.7 (CH_2 *4*) ppm. **HRMS (MALDI)** calculated for $[C_{24}H_{17}F_3O_3+Na]^+$: 433.1023, found: 433.1025.

11-(3-Fluorophenyl)-11-hydroxyphenyl-5a,6,11,11a-tetrahydrobenzo[*b*]xanthen-12-one (5f).

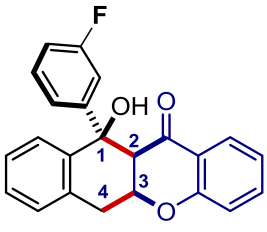

5f

Synthesised following the continuous-flow procedure 1 described in section **E** using 1 equivalent of **1d** (64.3 mg, 0.3 mmol) and 5 equivalents of chromone (**3a**, 219.2 mg, 1.5 mmol) in 5 mL of toluene with a residence time of 35 min**.** In order to calculate the isolated yield, 4 mL of the product solution of **5f** were collected and subjected to flash column chromatography on silica gel (9:1 Hexane/EtOAc) yielding pure **5f** (white solid), in 59% yield (51.0 mg, 0.142 mmol).

^{1}H-NMR (400 MHz, $CDCl_3$): 7.74 (dd, *J* = 7.5, 1.9 Hz, 1H, Ar), 7.50-7.47 (m, 1H, Ar), 7.44 (ddd, *J* = 15.3, 7.5, 2.0 Hz, 1H, Ar), 7.24–7.15 (m, 3H, Ar), 7.07-7.03 (m, 1H, Ar), 6.97-6.78 (m, 5H, Ar), 6.83 (d, *J*= 8.4 Hz, 1H, Ar), 5.68 (br s, 1H, OH), 4.80 (ddd, *J* = 9.4, 7.2, 5.1 Hz, 1H, CH _3_), 3.54 (d, *J*= 5.8 Hz, 1H, CH _2_), 3.25 (dd, *J*= 17.5, 9.5 Hz, 1H, CH_2 _4a_), 3.18 (dd, *J*= 17.5, 7.0 Hz, 1H, CH_2 _4b_) ppm. **^{13}C-NMR (100 MHz, $CDCl_3$)**: δ 195.3 (C=O), 163.7 (Cq Ar), 161.7 (Cq Ar), 159.2 (Cq Ar), 148.6, 148.6, 139.5, 137.1, 131.9, 129.7, 128.2, 127.7, 127.0, 122.9, 121.6, 120.7, 118.4, 114.8, 114.4, 77.5 (Cq _1_), 73.6 (CH _2_), 54.7 (CH _3_), 30.3 (CH_2 _4_) ppm. **HRMS (MALDI)** calculated for $[C_{23}H_{17}FO_3+Na]^+$: 383.1059, found: 383.1063.

I. Product Manipulations

1.1. General procedure for the formation of 6a

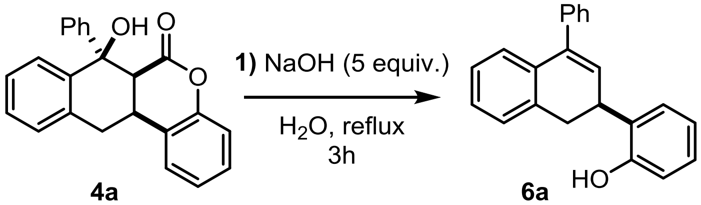

Chromenone **4a** (200 mg, 1 equiv, 0.60 mmol) was dissolved in H_2O (3 mL). To the resulting heterogenous solution, NaOH (120 mg, 5 equiv, 3 mmol) was added portionwise under vigorous stirring at room temperature. The solution was then heated to reflux. After 3 h, full conversion was observed by TLC analysis. The reaction mixture was cooled to room temperature and extracted with 3 × 10 mL of DCM. The combined organic phases were dried over $MgSO_4$ and the solvent was removed by rotary evaporation. Concentration under high vacuum yielded 2-(4-phenyldihydronaphthalenyl)phenol (**6a**) as white solid in 95% yield (170 mg, 0.576 mmol).

^{1}H-NMR (400 MHz, $CDCl_3$): 7.46-7.35 (m, 5H, Ar), 7.30 (dd, *J*= 7.5, 1.6 Hz, Ar), 7.25-7.09 (m, 5H, Ar), 6.91 (dt, *J*= 7.5, 1.1 Hz, 1H, Ar), 6.83 (dd, *J*=7.9, 1.1 Hz), 6.18 (d, *J*= 3.5 Hz, 1H, Csp^2), 4.98 (br s, 1H, OH), 4.22 (ddd, *J* = 8.5, 3.5, 3.1 Hz, 1H, CH), 3.16-3.14 (m, 2H, CH_2) ppm. **^{13}C-NMR (100 MHz, $CDCl_3$)**: δ 153.5 (Cq Ar), 141.2 (Cq Ar), 140.5 (Cq Ar), 136.1 (Cq Ar), 134.6 (Cq Ar), 130.9 (CH Ar), 130.4 (Cq Ar), 129.2 (CH Ar), 129.0 (CH Ar x2), 128.5 (CH Ar x2), 128.1 (CH Ar), 127.9 (CH Ar), 127.7 (CH Ar), 127.6 (CH Ar), 126.7 (CH Ar), 125.9 (CH Ar), 121.2 (CH Ar), 115.9 (CH sp^2), 35.6 (CH), 35.3 (CH_2) ppm. **HRMS (MALDI)** calculated for $[C_{22}H_{18}O+H]^+$: 321.1250, found: 321.1254.

1.2. GENERAL PROCEDURE FOR THE FORMATION OF 7A

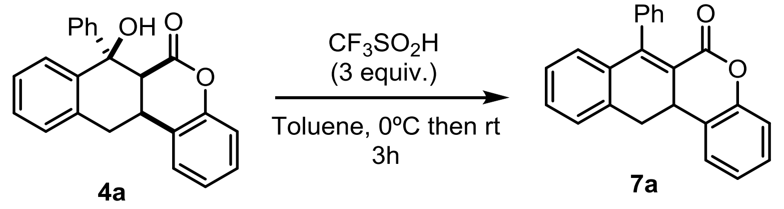

Chromenone **4a** (200 mg, 1 equiv, 0.60 mmol) was dissolved in dry toluene (6 mL) under an inert atmosphere. To the resulting solution, triflic acid (160 µL, 3 equiv, 1.8 mmol) was added dropwise under vigorous stirring at 0 °C. The solution was stirred for 1 h at 0 °C and allowed to gradually warm to room temperature. After 3 h, full conversion was observed by TLC analysis and the reaction was quenched with $NaHCO_3$. The mixture was extracted with 3 × 10 mL of DCM, the combined organic phases were dried over $MgSO_4$ and the solvent was removed by rotary evaporation. Concentration under high vacuum yielded 7-phenyldihydronaphtho[2,3-*c*]chromenone (**7a**) as white solid in 95% yield (170 mg, 0.572 mmol).

^{1}H-NMR (400 MHz, $CDCl_3$): 7.49-7.41 (m, 4H, Ar), 7.39-7.27 (m, 4H, Ar), 7.23-7.14 (m, 3H, Ar), 7.07 (dd, *J*= 8.2, 1.2 Hz, 1H, Ar), 6.89 (d, *J*= 7.8 Hz, 1H, Ar), 4.30 (dd, *J* = 15.9, 6.4 Hz, 1H, CH_2 a), 3.52 (dd, *J* = 15.9, 6.2 Hz, 1H, CH_2 b), 3.21 (t, *J* = 15.4 Hz, 1H, CH) ppm. **^{13}C-NMR (100 MHz, $CDCl_3$)**: δ 160.2 (COO), 153.2 (Cq Ar), 150.6 (Cq Ar), 139.1 (Cq Ar), 135.7 (CH Ar), 135.5 (Cq Ar), 130.4 (CH Ar), 129.5 (CH Ar), 129.2 (Cq), 128.7 (CH Ar), 128.4 (Cq Ar), 128.3 (CH Ar), 127.7 (CH Ar), 127.5 (CH Ar), 127.4 (CH Ar), 126.6 (CH Ar), 125.5 (Cq), 124.5 (CH Ar), 123.2 (CH Ar), 119.4 (CH Ar), 117.2 (CH Ar), 34.6 (CH), 34.2 (CH_2) ppm. **HRMS (MALDI)** calculated for $[C_{23}H_{16}O_3+Na]^+$: 347.1043, found: 347.1048.

1.3. GENERAL PROCEDURE FOR THE PREPARATION OF 8A

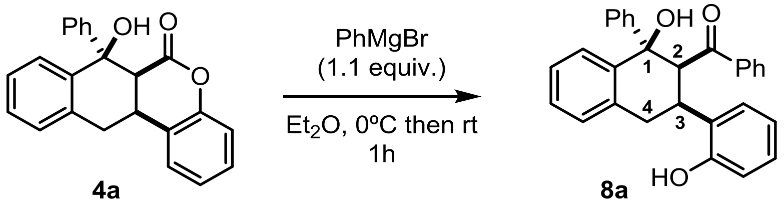

Tetrahydronaphthochromenone **4a** (200 mg, 1 equiv, 0.60 mmol) was dissolved in dry Et_2O (8 mL) under an inert atmosphere. To the resulting solution, PhMgBr (1.0 M in THF; 768 µL, 1.1 equiv, 0.768 mmol) was added dropwise under vigorous stirring at 0 °C. The solution was then stirred for 1 h at room temperature. After 1 h, full conversion was observed by TLC analysis and 8 mL of a saturated solution of NH_4Cl were added dropwise. The mixture was extracted with 3 × 10 mL of Et_2O. The combined organic phases were dried over $MgSO_4$ and the solvent was removed by rotary evaporation. After flash column chromatography (9:1 Hexane/EtOAc) **8a** (transparent oil) was obtained in 71% yield (209.2 mg, 0.496 mmol).

^{1}H-NMR (400 MHz, $CDCl_3$): 7.41-7.35 (m, 3H, Ar), 7.27-7.19 (m, 9H, Ar), 7.05 (t, *J*= 7.8, 2H, Ar), 6.79 (t, *J*= 7.0 Hz, 1H, Ar), 6.60 (d, *J*= 7.0 Hz, 1H, Ar), 6.47 (d, *J* = 7.4 Hz, 1H, Ar), 6.39 (t, *J* = 7.3 Hz, 1H, Ar), 4.62 (d, *J* = 3.9 Hz, 1H, CH 2), 3.79 (dt, *J* = 12.8, 3.9 Hz, 1H, CH 3), 3.39 (dd, *J* = 16.4, 12.1 Hz, 1H, CH_2 *4a*), 2.88 (dd, *J* = 16.4, 4.9 Hz, 1H, CH_2 *4b)* ppm. **^{13}C-NMR (100 MHz, $CDCl_3$)**: δ 204.0 (C=O), 153.2 (Cq Ar), 148.1 (Cq Ar), 140.9 (Cq Ar), 139.0 (Cq Ar), 136.9 (Cq Ar), 132.8 (CH Ar), 130.4 (Cq Ar), 128.5 (CH Ar), 128.1 (CH Ar x4), 128.0 (CH Ar x2), 127.8 (CH Ar), 127.7 (CH Ar x2), 127.7 (CH Ar x2), 127.6 (CH Ar), 127.5 (CH Ar), 127.2 (CH Ar), 124.8 (Cq Ar), 120.8 (CH Ar), 115.3 (CH Ar), 78.5 (Cq), 57.1 (CH 2), 34.1 (CH 3), 30.3 (CH_2 4) ppm. **HRMS (MALDI)** calculated for $[C_{29}H_{24}O_3+Na]^+$: 443.1618, found: 443.1617.

1.4. GENERAL PROCEDURE FOR THE PREPARATION OF 9A

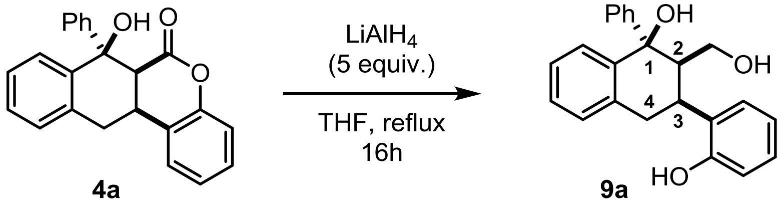

Tetrahydronaphthochromenone **4a** (200 mg, 1 equiv, 0.60 mmol) was dissolved in dry THF (6 mL) under an inert atmosphere. To the resulting solution, $LiAlH_4$ (1.0 M in THF; 3 mL, 5 equiv, 3.0 mmol) was added dropwise under vigorous stirring at 0 °C. The solution was then heated to reflux. After 16 h, full conversion was observed by TLC analysis. The reaction mixture was cooled to room temperature and EtOAc was added dropwise. The mixture was extracted with 3 × 10 mL of EtOAc. The combined organic phases were dried over $MgSO_4$ and the solvent was removed by rotary evaporation. Concentration under high vacuum yielded **9a** as white solid in >98% yield (201.2 mg, 0.6 mmol).

^{1}H-NMR (400 MHz, $CDCl_3$): 7.38-7.24 (m, 9H, Ar), 7.14-7.07 (m, 2H, Ar), 6.84 (t, *J*= 7.6 Hz, 1H, Ar), 6.78 (d, *J*= 8.0 Hz, 1H, Ar), 3.82 (dd, *J*= 11.3, 6.8 Hz, 1H, C*H₂*OH a), 3.76 (dd, *J*= 11.4, 6.8 Hz, 1H, C*H₂*OH b), 3.69 (td, *J*= 6.7, 3.3 Hz, 1H, CH 2), 3.34 (dd, *J*= 17.0, 7.1 Hz, 1H, CH_2 4), 3.27 (dd, *J*= 17.0, 6.3 Hz, 1H, CH_2 4), 2.85 (td, *J*= 6.7, 3.3 Hz, 1H, CH, 3) ppm. *Due to solubility issues the 13C-NMR analysis was performed in MeOD-d4*. **^{13}C-NMR (125 MHz, MeOD-d_4)**: δ 155.2 (Cq Ar), 149.1 (Cq Ar), 140.4 (Cq Ar), 136.5 (Cq Ar), 128.8 (CH Ar), 128.7 (CH Ar), 127.9 (CH Ar), 127.7 (CH Ar x2), 127.0 (CH Ar), 126.9 (CH Ar x2), 126.5 (CH Ar), 126.1 (CH Ar), 118.6 (CH Ar), 114.6 (CH Ar), 80.8 (Cq), 60.8 (CH), 49.5 (CH) 32.1 (CH_2), 29.2 (CH_2) ppm. **HRMS (MALDI)** calculated for $[C_{23}H_{22}O_3+Na]^+$: 369.1461, found: 369.1463.

J. X-RAY STRUCTURE DETERMINATION AND REFINEMENT FOR COMPOUNDS 4A AND 5A

Colourless, block-shaped single crystals of compounds **4a** and **5a** were easily obtained by slow evaporation of Et_2O/hexane solutions of the respective synthetic diasteroisomeric mixtures (see Scheme S1). X-ray quality crystals with approximate dimensions 0.40 × 0.30 × 0.20 mm^3 (**4a**) and 0.40 × 0.55 × 0.55 mm^4 (**5a**) were chosen under the microscope for the measurement. Diffraction data were collected at room temperature (296 K) with an Oxford Diffraction Gemini E diffractometer, equipped with a 2 K × 2 K EOS CCD area detector and sealed-tube Enhance (Mo) and (Cu) X-ray sources. MoKa (λ = 0.71073 Å) radiation was used for **4a**, while **5a** was investigated using CuKa (λ = 1.54184 Å). Data collection, reduction and finalisation were carried out through the CrysAlisPro software. The structures were solved by direct methods and subsequently completed by Fourier recycling using the SHELXTL-2013 software package[4] and refined by the full-matrix least-squares refinements based on F^2 with all observed reflections. All non-hydrogen atoms were refined anisotropically; hydrogen atoms were included at geometrically calculated positions and refined using a riding model.

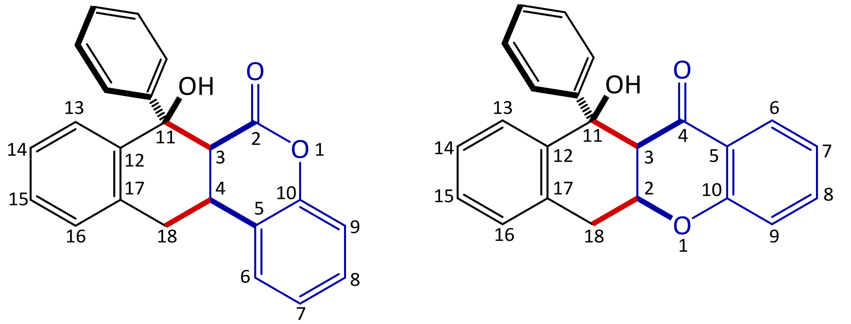

4a **5a**

Scheme S1. Molecular structure of compounds **4a** and **5a** with the atom numbering used in this section (only selected atoms). According to this numbering, the synthetic procedures led to diasteroisomers **3*RS*,4*RS*,11*SR*-4a** and **2*RS*,3*RS*,11*SR*-5a**.

Compound **4a** is racemic, as expected, and it crystallizes in the centrosymmetric orthorhombic space group *Pbca*, with eight molecules in the unit cell. Figure S14 shows

the content of the asymmetric unit as the 3*R*,4*R*,11*S* enantiomer (arbitrary choice). In the case of compound **5a**, instead, the crystallization process also afforded chiral resolution, as the analyzed crystal of **5a** turned out to comprise the pure 2*S*,3*S*,11*R* enantiomer (see Figure S15). This crystallised in the orthorhombic chiral space group $P2_12_12_1$, with four molecules in the unit cell. The final geometrical calculations and graphical manipulations were performed by using the XP utility within SHELX.[4] A summary of the crystallographic data and structure refinement for **4a** and **5a** is given in Table S5. Crystallographic data have been deposited at the Cambridge Crystallographic Data Centre (CCDC reference numbers 1837120 (**4a**) and 1851516 (**5a**)). These data can be obtained free of charge from the Cambridge Crystallographic Data Centre via www.ccdc.cam.ac.uk/structures/.

Table S4. Summary of crystal data and structure refinement for **4a** and **5a**.

	4a	5a
Empirical formula	$C_{23}H_{18}O_3$	$C_{23}H_{18}O_3$
Formula weight	342.37	342.37
Temperature	293(2) K	298(2) K
Wavelength	0.71073 Å	1.54184 Å
Crystal system	Orthorhombic	Orthorhombic
Space group	*Pbca*	$P2_12_12_1$
Unit cell dimensions	*a* = 14.6629(5) Å	*a* = 10.00942(16) Å
	b = 7.5142(2) Å	*b* = 11.49917(17) Å
	c = 30.7557(9) Å	*c* = 14.7105(2) Å
Volume	3388.69(18) $Å^3$	1693.18(5) $Å^3$
Z	8	4
Density (calculated)	1.342 g/cm^3	1.343 g/cm^3
Absorption coefficient	0.088 mm^{-1}	0.707 mm^{-1}
F(000)	1440	720
Reflections collected	21025	13333
Independent reflections	3774 [*R*(int) = 0.0420]	3362 [R(int) = 0.0276]
Data / restraints / parameters	3774 / 0 / 235	3362 / 0 / 236
Goodness-of-fit on F2	1.064	1.066
Final R indices [I>2sigma(I)][a,b]	R_1 = 0.0475, wR_2 = 0.0948	R_1 = 0.0359, wR_2 = 0.0948
R indices (all data) [a,b]	R_1 = 0.0818, wR_2 = 0.1103	R_1 = 0.0369, wR_2 = 0.0956
Absolute structure parameter	n.a.	0.03(8)
Extinction coefficient	n.a.	0.0133(11)
Largest diff. peak and hole	0.198 and -0.163 $e\cdot Å^{-3}$	0.223 and -0.200 $e\cdot Å^{-3}$

[a] $R_1 = \sum||F_o| - |F_c||/\sum|F_o|$. [b] $wR_2 = \{\sum w(F_o^2 - F_c^2)^2/\sum[w(F_o^2)^2\}^{1/2}$ and $w = 1/[\sigma^2(F_o)^2 + (mP)^2 + nP]$ with $P = (F_o^2 + 2F_c^2)/3$, m = 0.0398 (**4a**) and 0.0624 (**5a**) and n = 0.5786 (**4a**) and 0.1241 (**5a**).

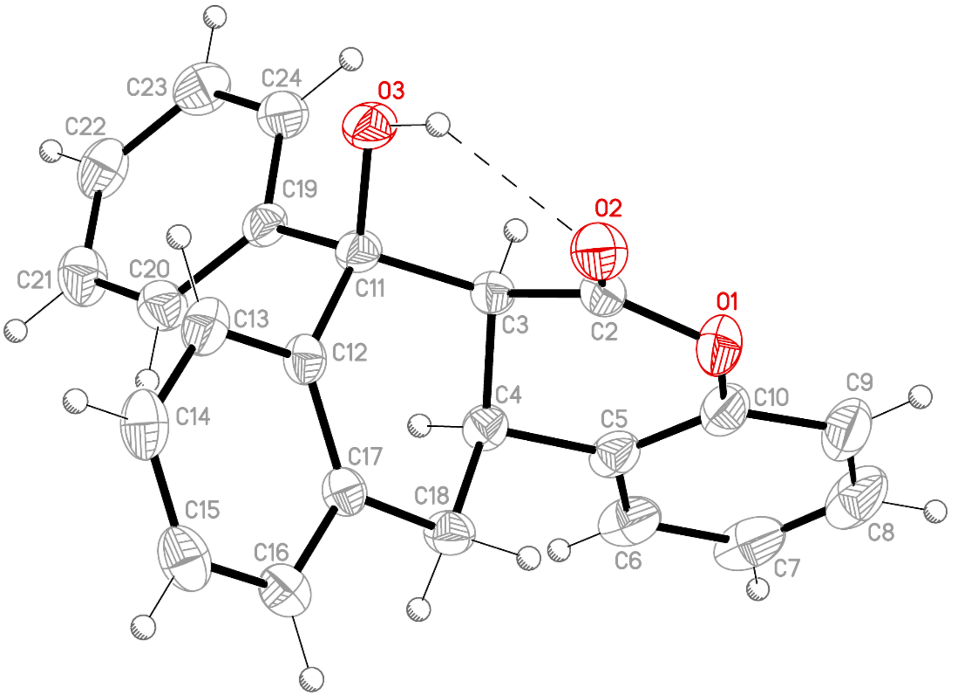

Figure S14. Molecular structure of **4a** (3*R*,4*R*,11*S* enantiomer) with the atom numbering. Thermal ellipsoids are drawn at the 30% probability level. The intramolecular H bond is depicted as a dashed line [O3···O2, 2.671(2) Å, 144.5°].

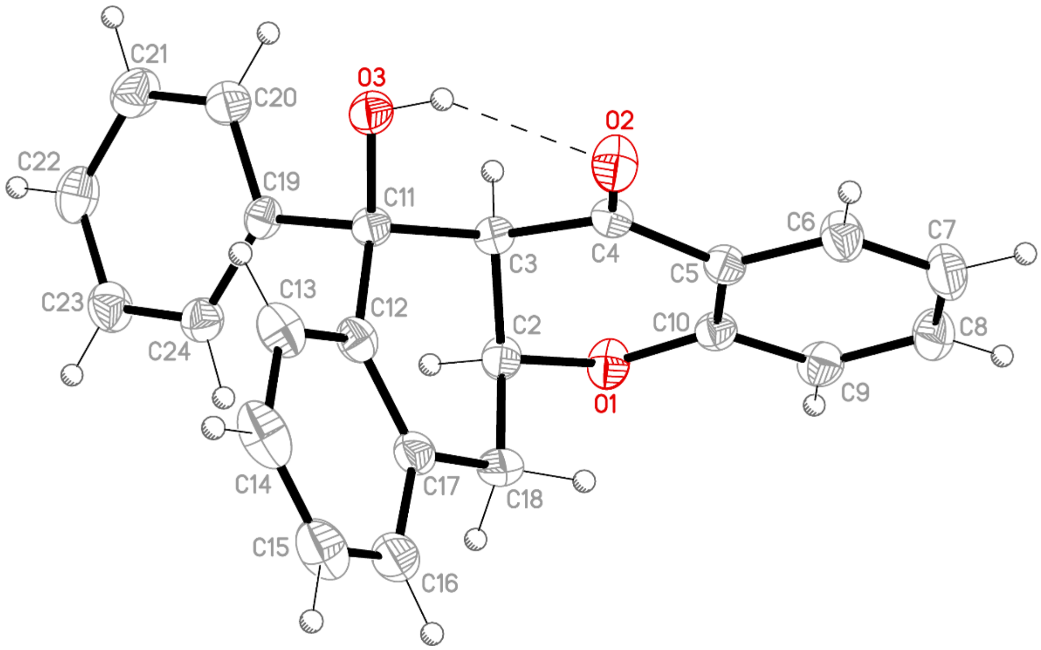

Figure S15. Molecular structure of **5a** with the atom numbering. Thermal ellipsoids are drawn at the 30% probability level. The intramolecular H bond is depicted as a dashed line [O3···O2, 2.766(2) Å, 139.0°].

K. REFERERNCES

1. Dell'Amico, L.; Fernández-Álvarez, V.M.; Maseras, F.; Melchiorre. P.; *Angew. Chem. Int. Ed.,* **2017,** *56, 12,* 3304-3308.
2. Ferraroni, M.; Carta, F.; Scozzafava, A.; Supuran, C.T.; *J. Med. Chem.*, **2016,** *59,* 462-473.
3. Mateos, J.; Cherubini-Celli, A.; Carofiglio, T.; Bonchio, M.; Marino, N.; Companyó X.; Dell'Amico, L. *Chem. Commun*., **2018**, *54*, 6820–6823.
4. (a) Sheldrick, G. M.; *Acta Cryst.* **2008**, *A64*, 112-122; (b) Sheldrick, G. M.; *Acta Cryst.* **2015**, *C71*, 3-8.

L. NMR SPECTRA

4b ^{1}H NMR spectrum

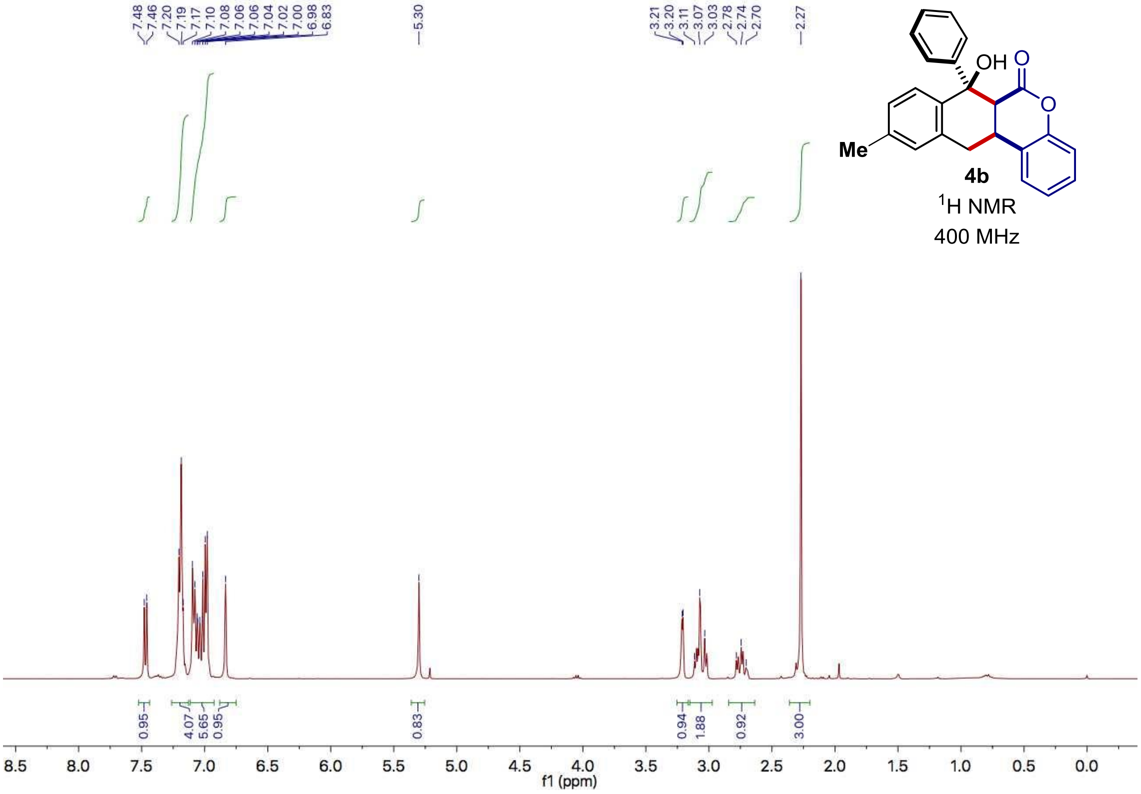

4b ^{13}C NMR spectrum

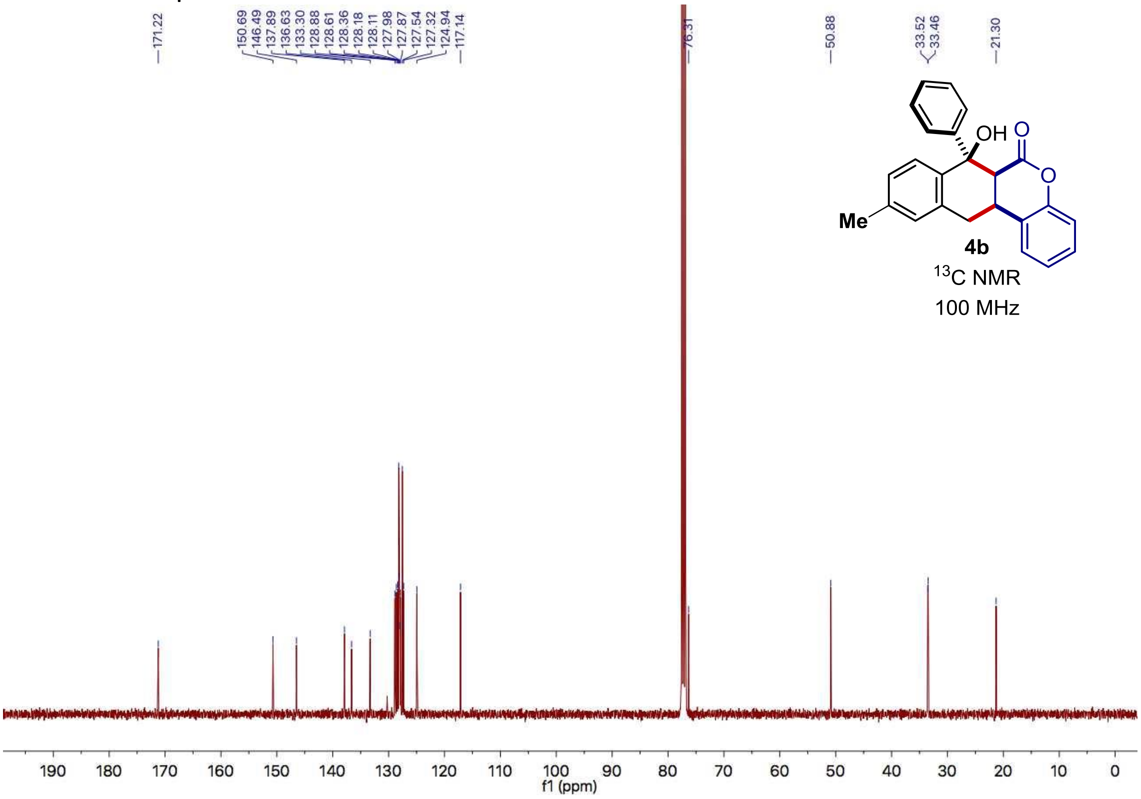

4c ^{1}H NMR spectrum

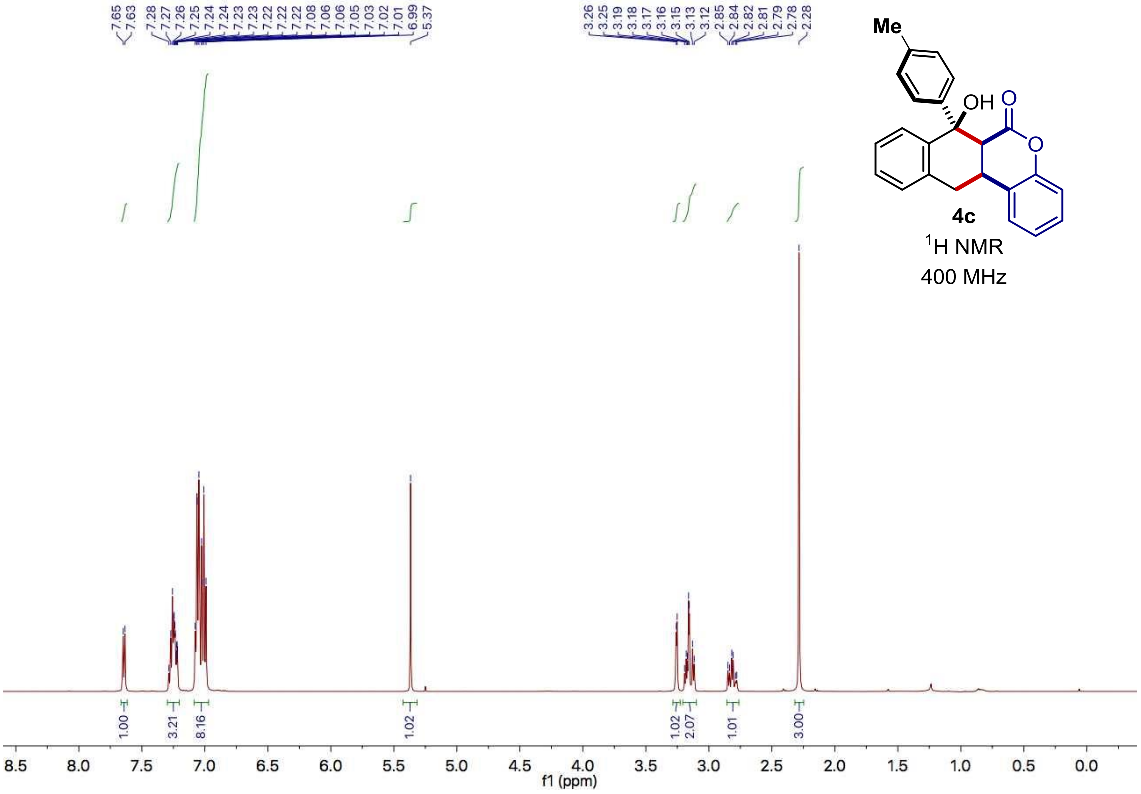

4c ^{13}C NMR spectrum

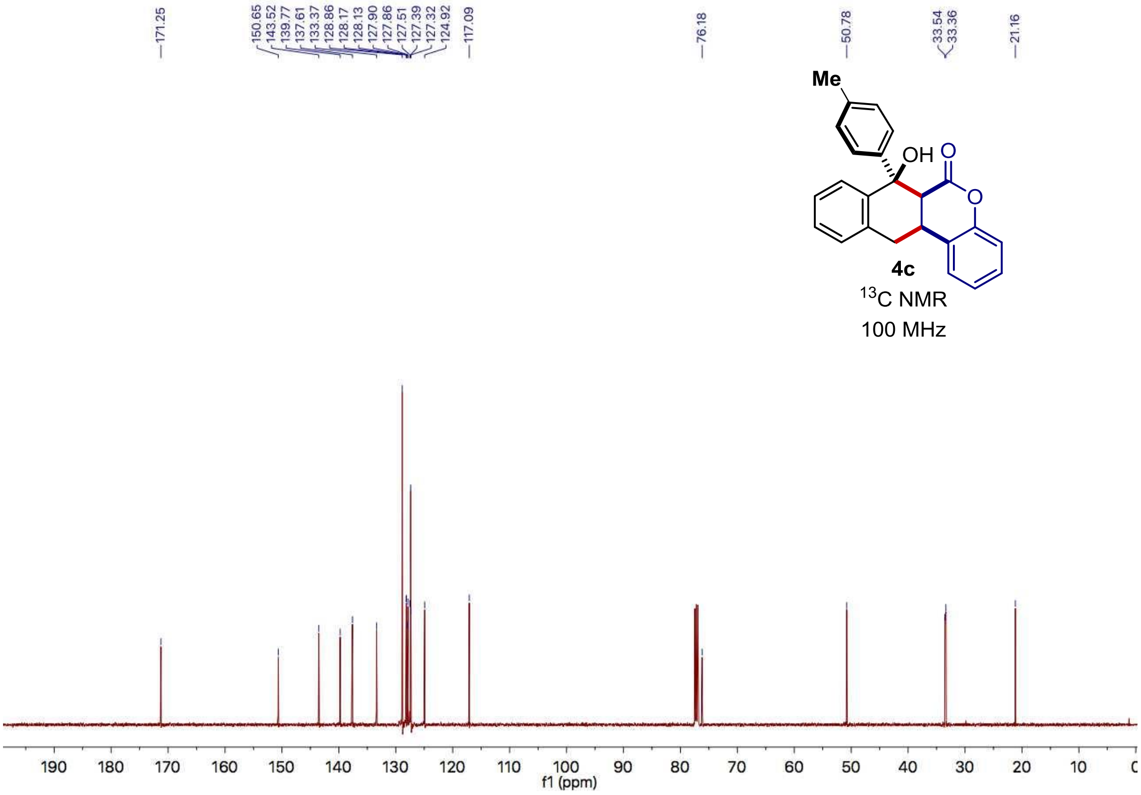

4d ^{1}H NMR spectrum

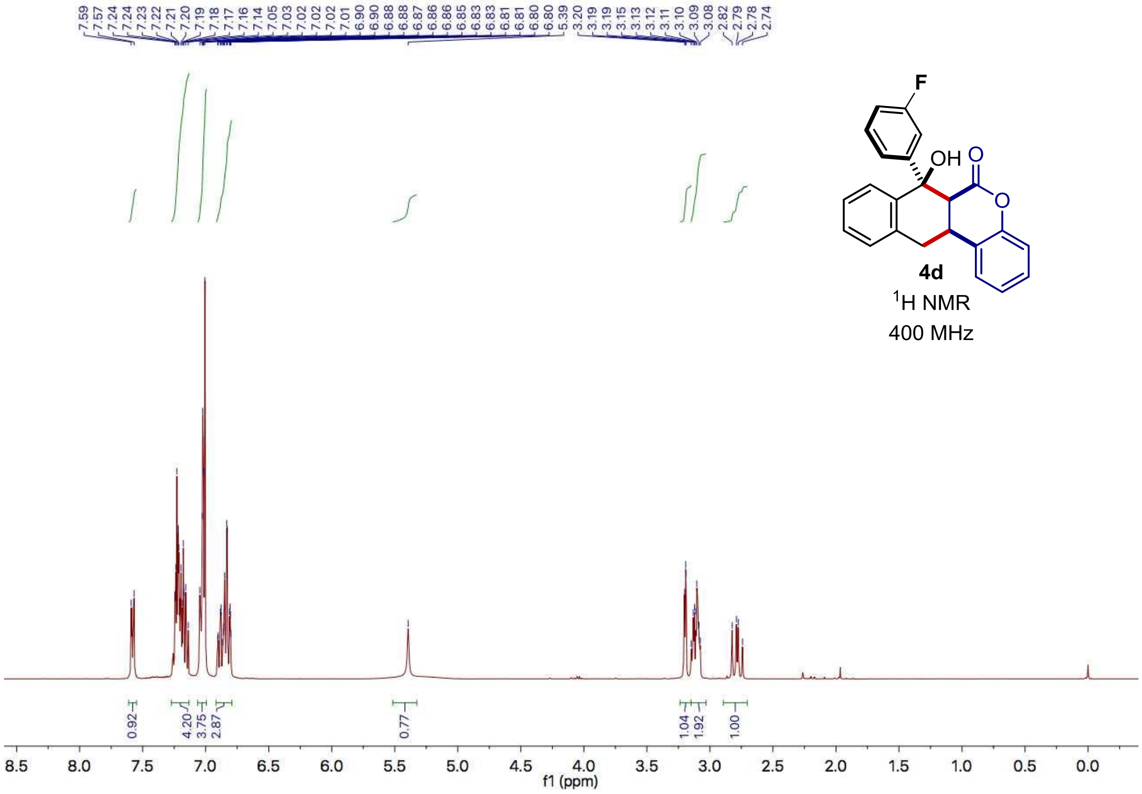

4d ^{13}C NMR spectrum

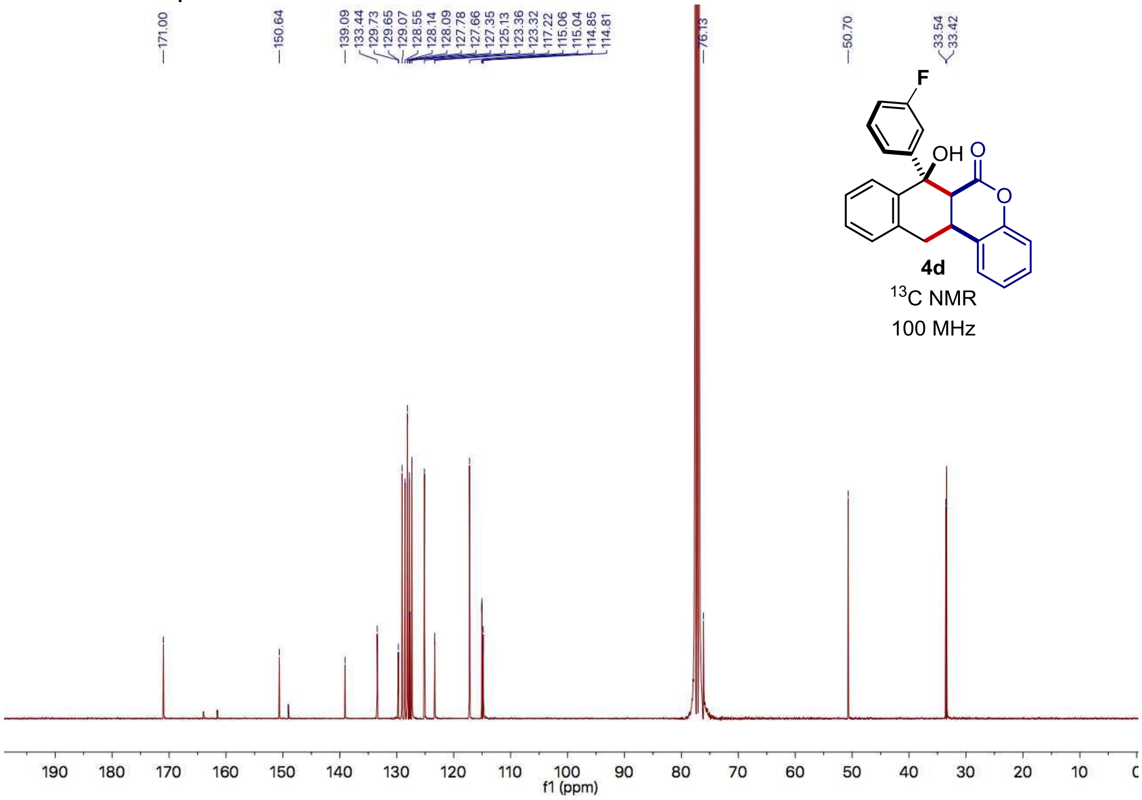

4e ^{1}H NMR spectrum

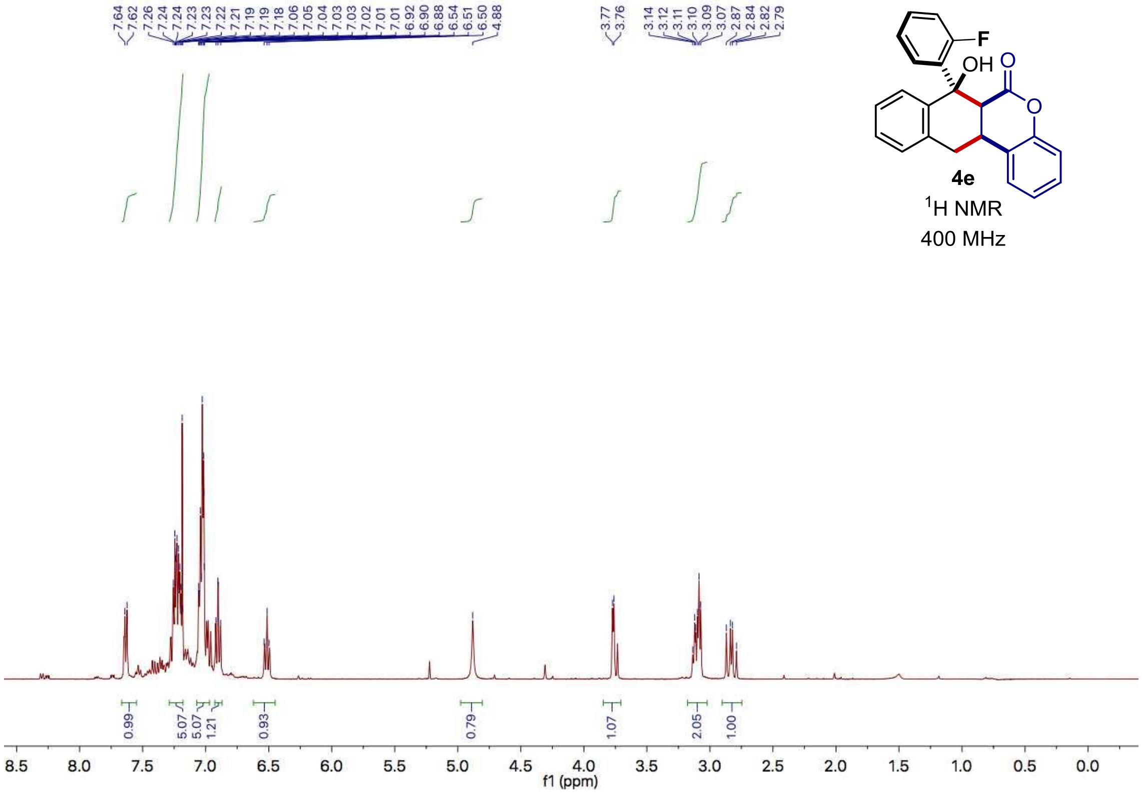

4e ^{13}C NMR spectrum

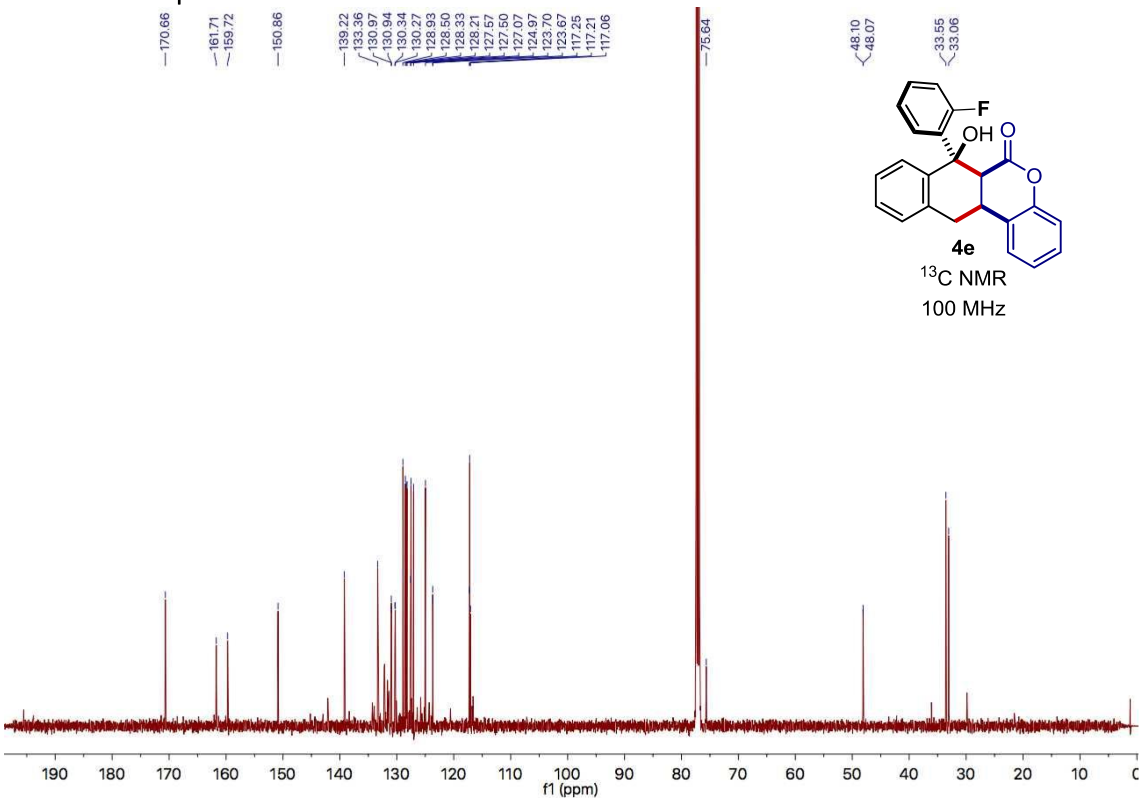

4f ^{1}H NMR spectrum

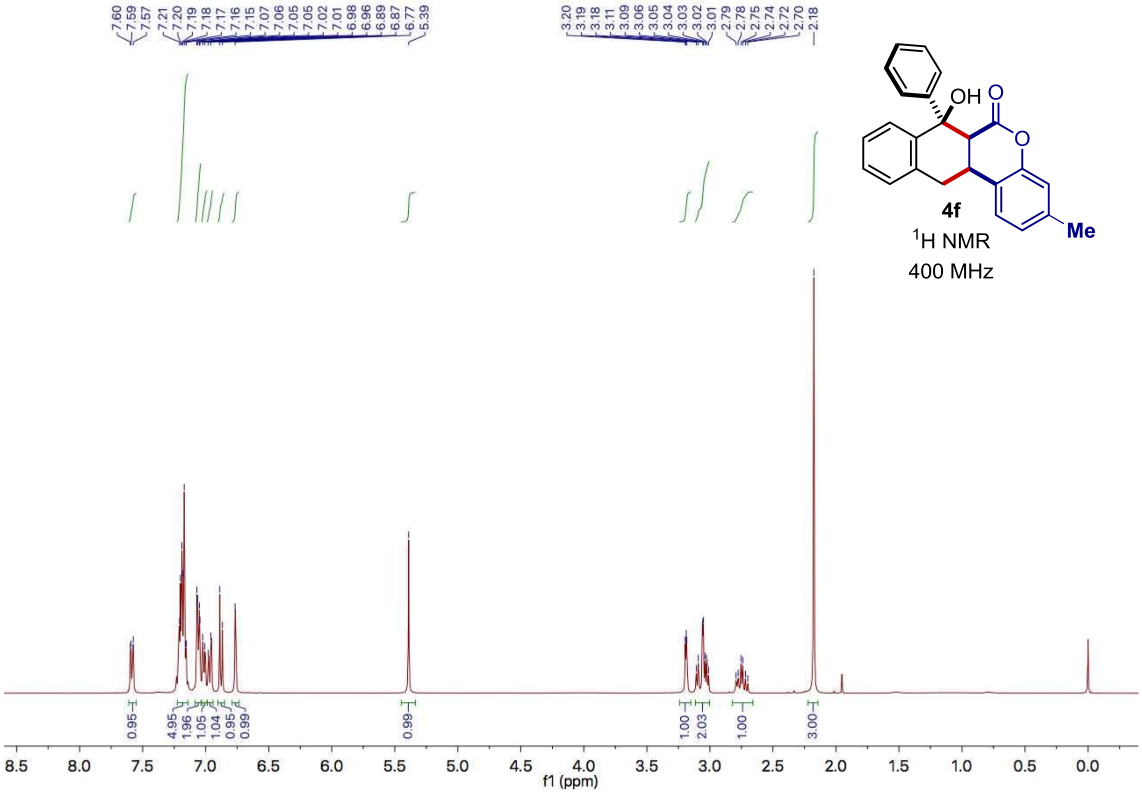

4f ^{13}C NMR spectrum

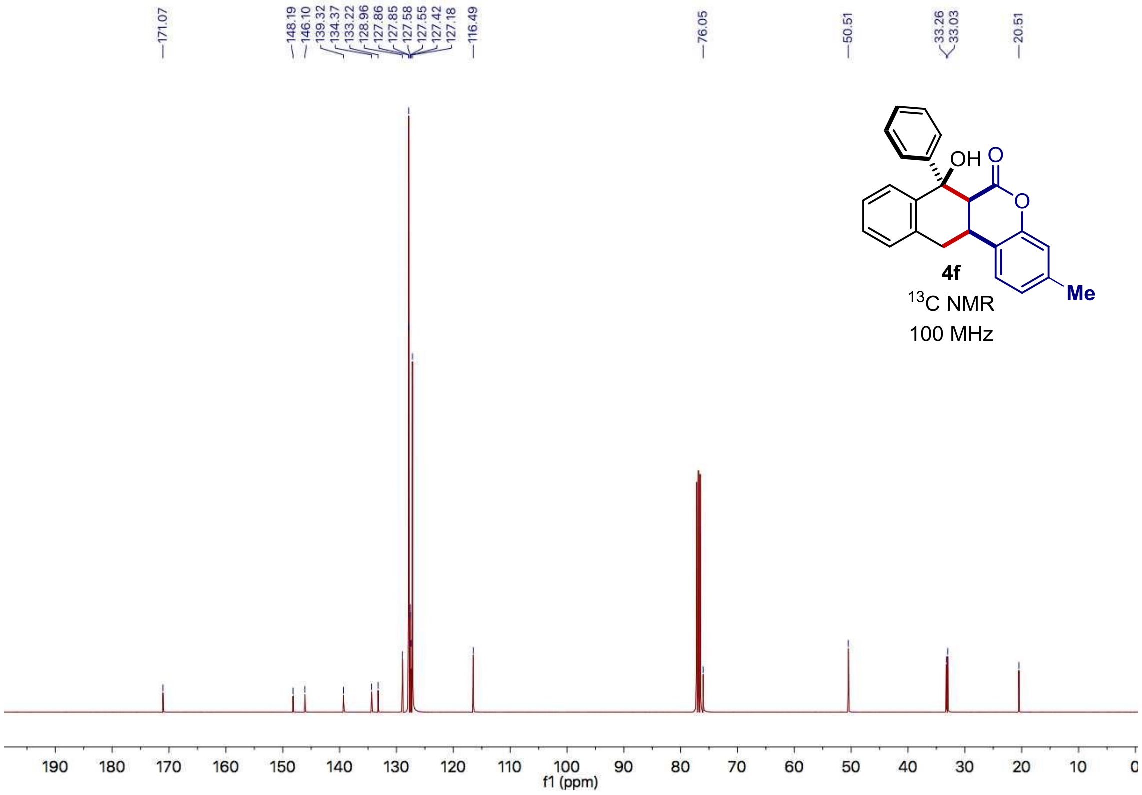

4g ^{1}H NMR spectrum

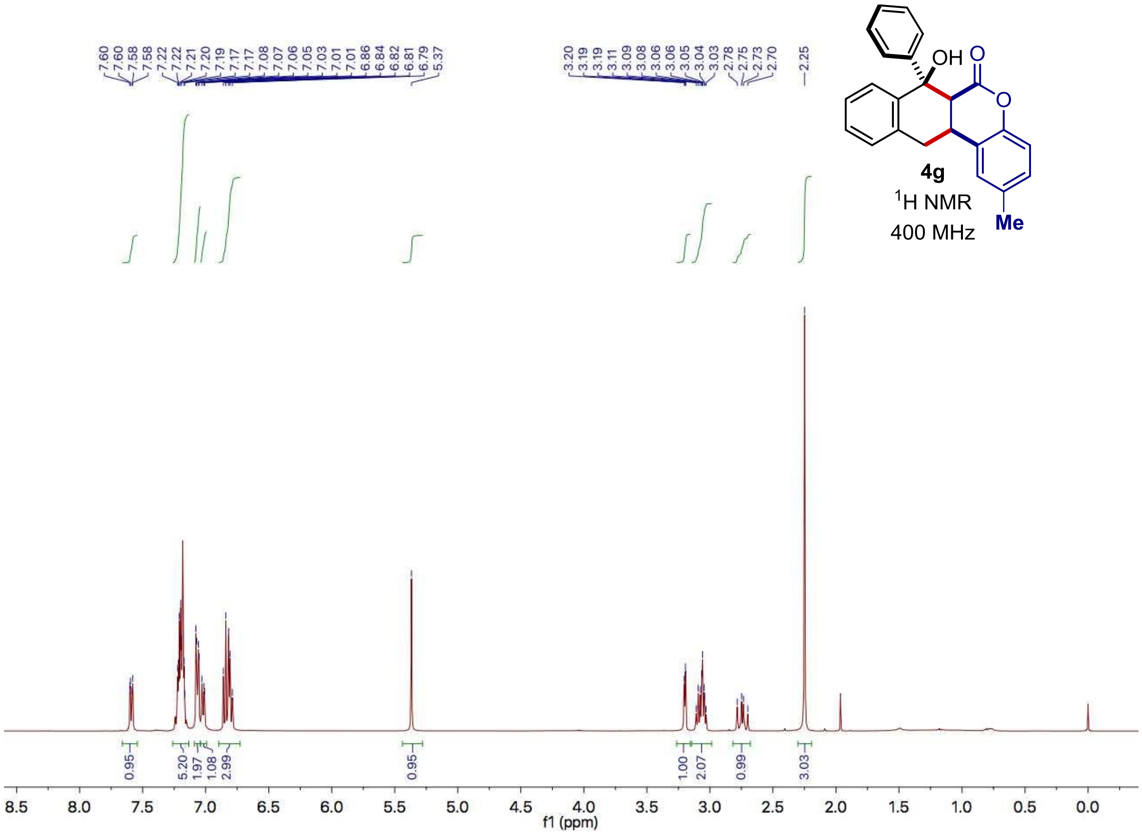

4g ^{13}C NMR spectrum

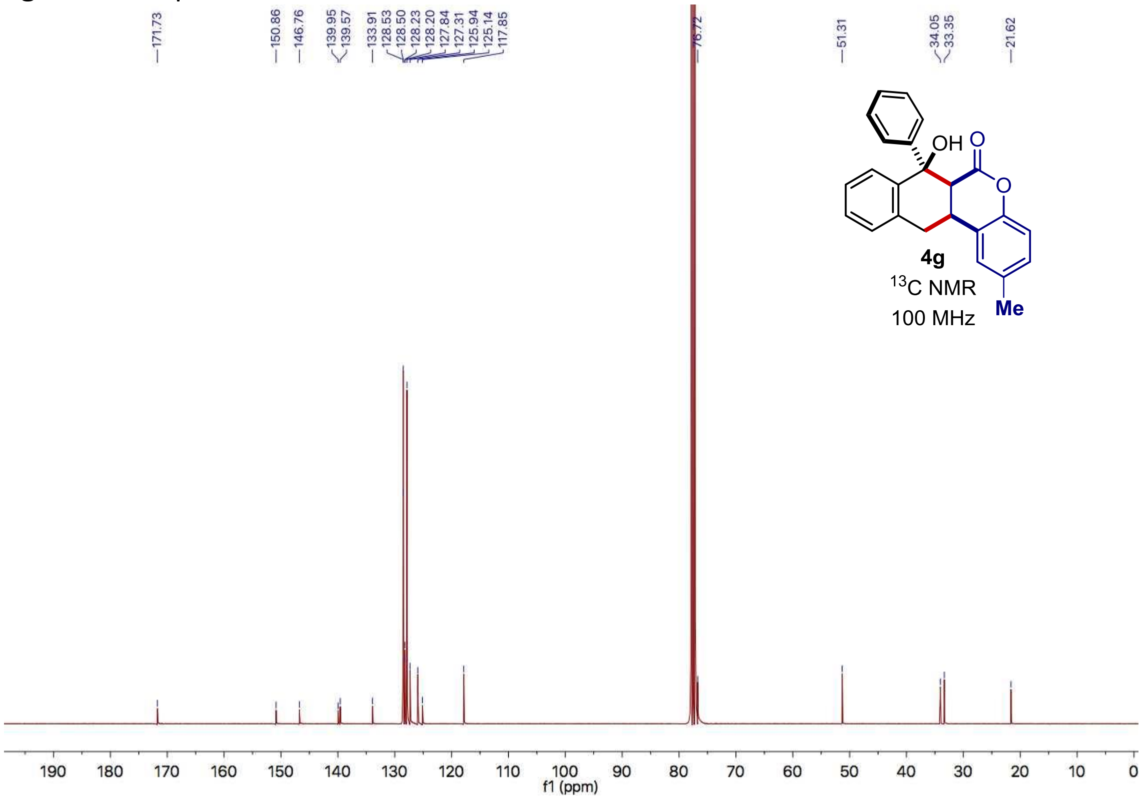

4h ^{1}H NMR spectrum

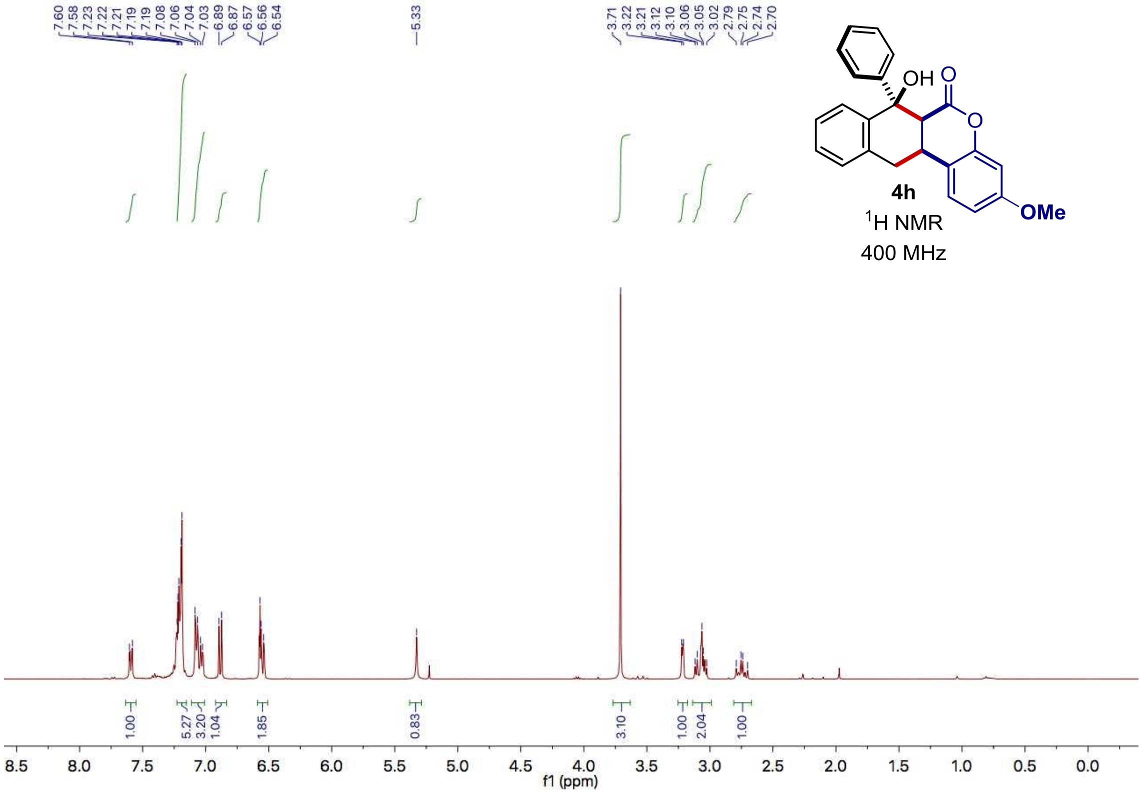

4h ^{13}C NMR spectrum

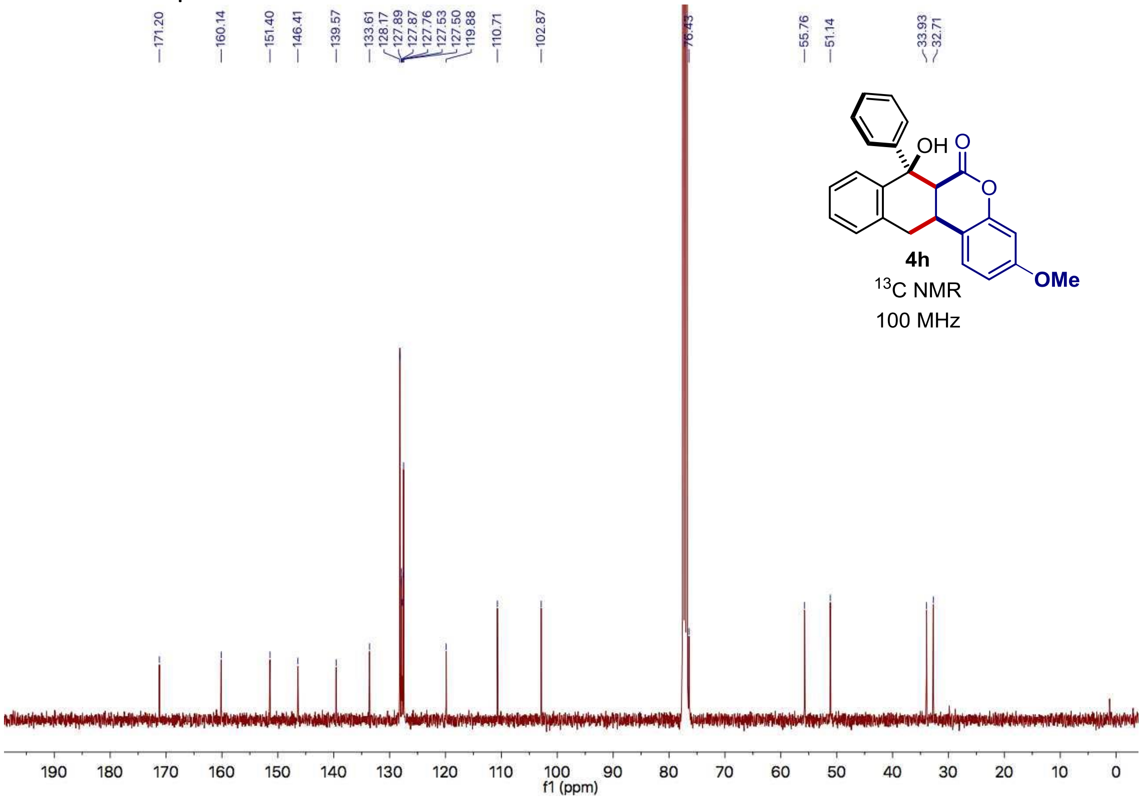

5a ^{1}H NMR spectrum

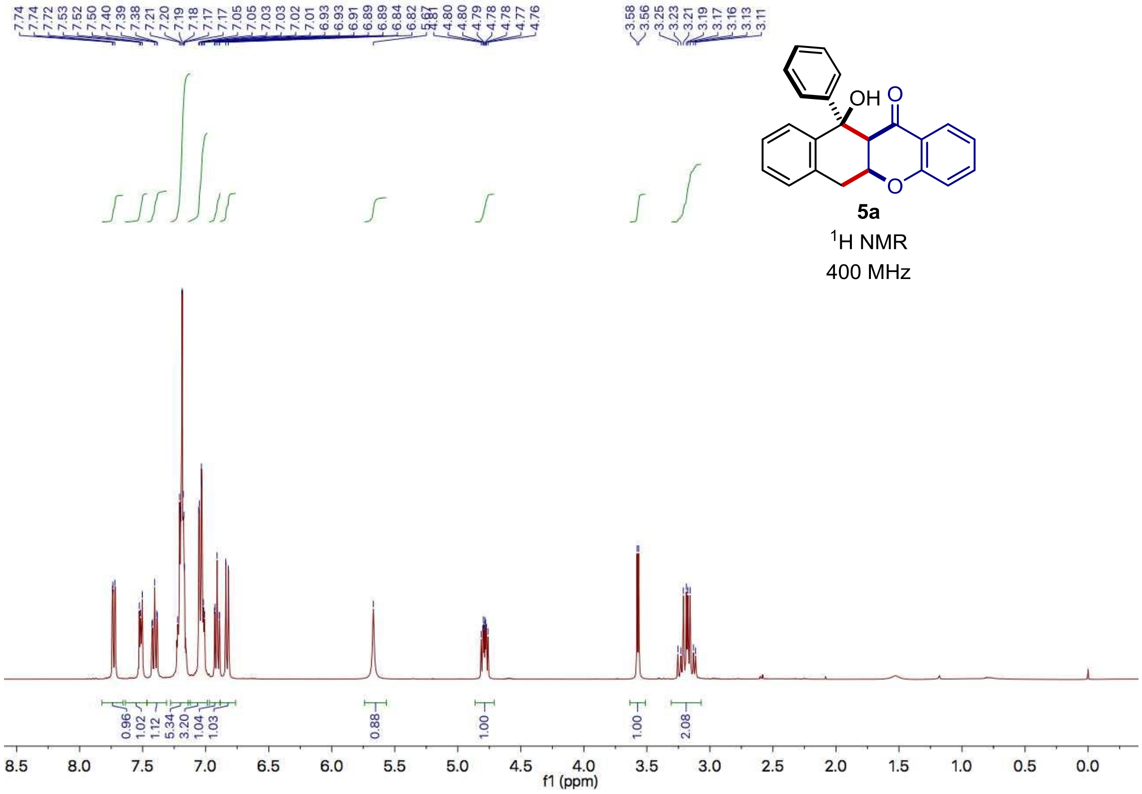

5a ^{13}C NMR spectrum

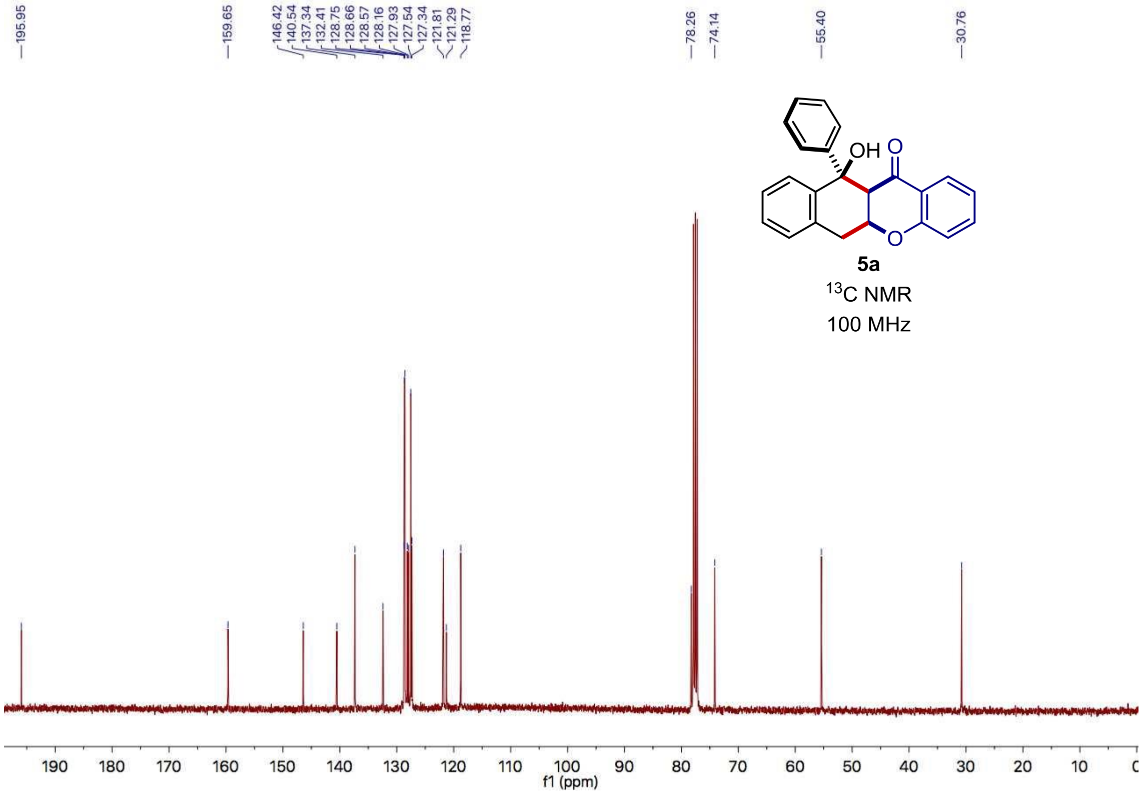

5a ^{1}H-^{1}H COSY NMR spectrum

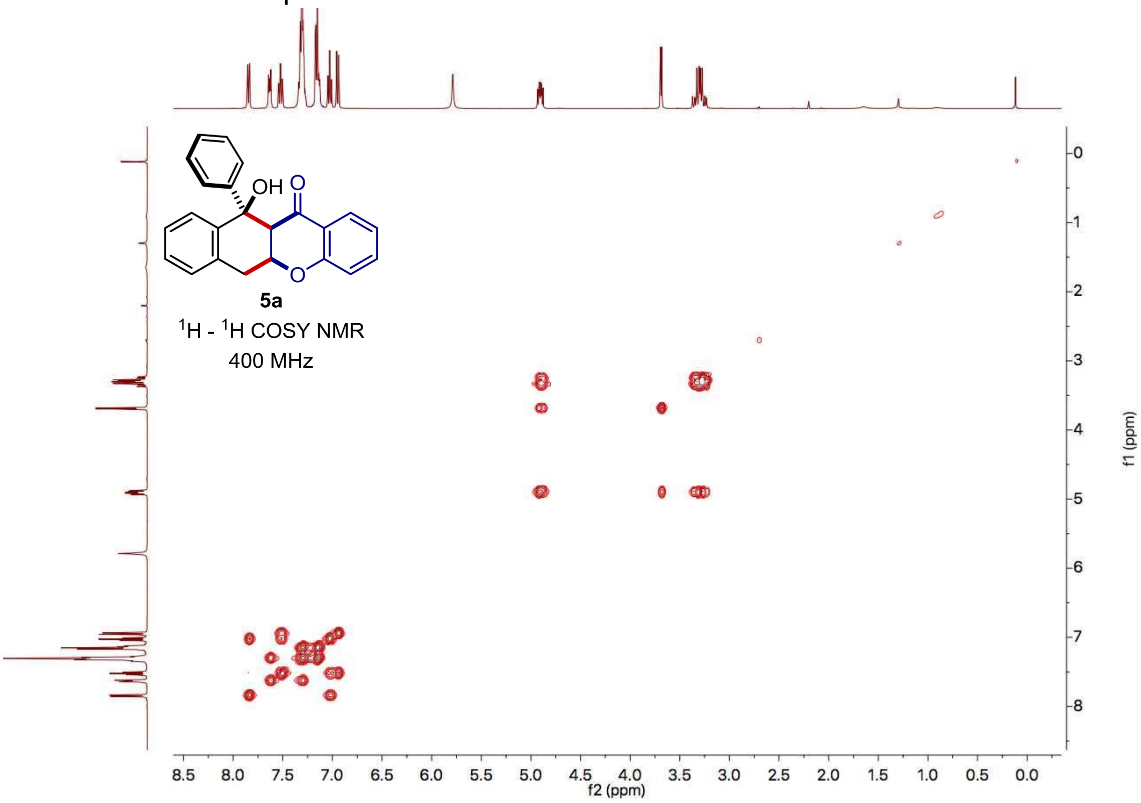

5a ^{1}H-^{1}H NOESY NMR spectrum

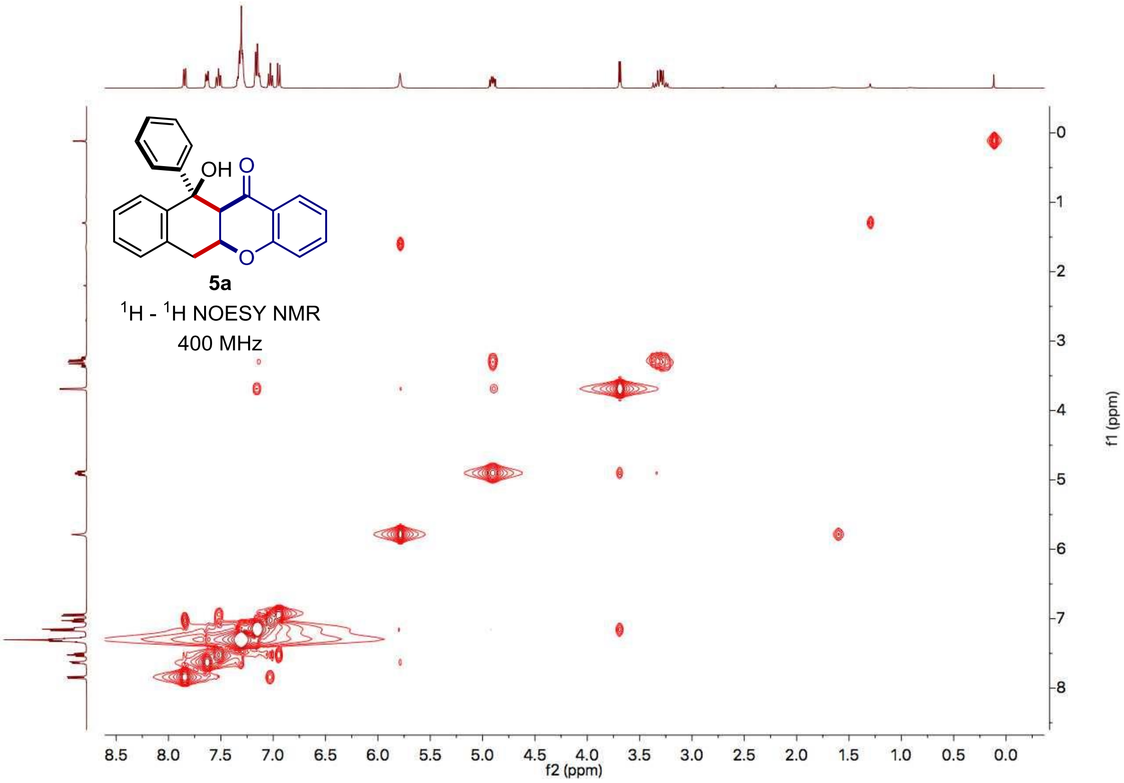

5b ^{1}H NMR spectrum

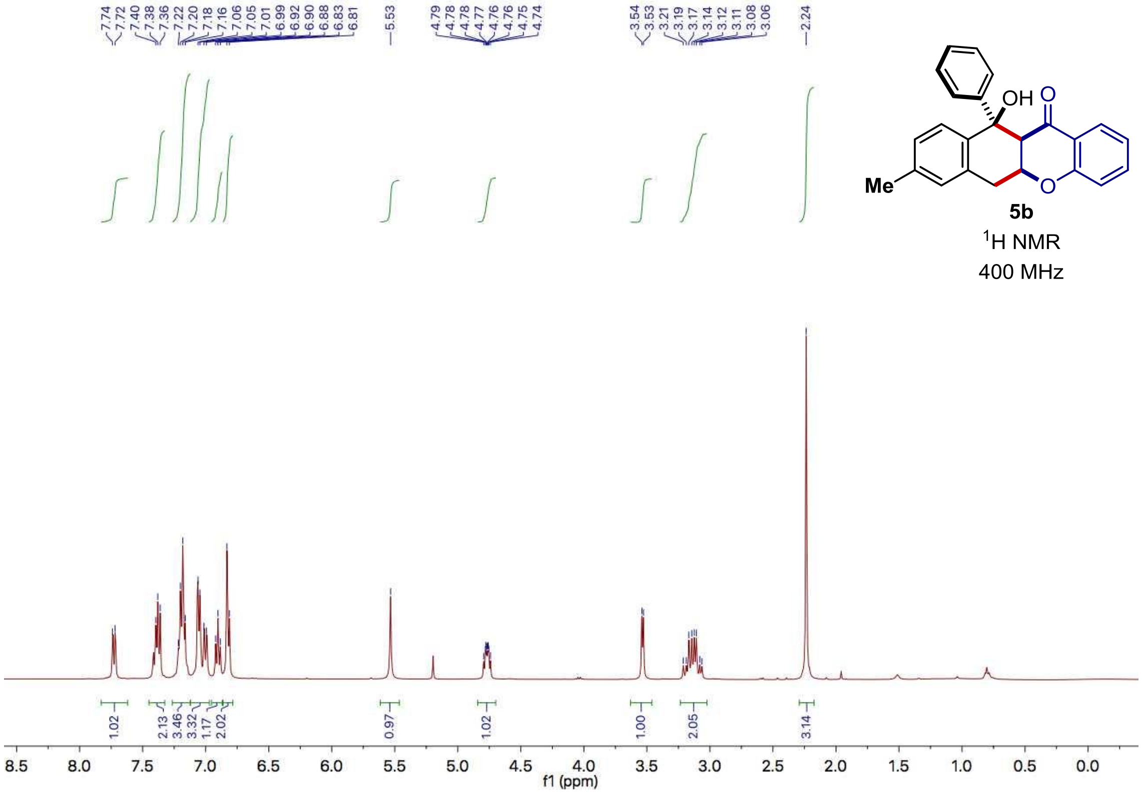

5b ^{13}C NMR spectrum

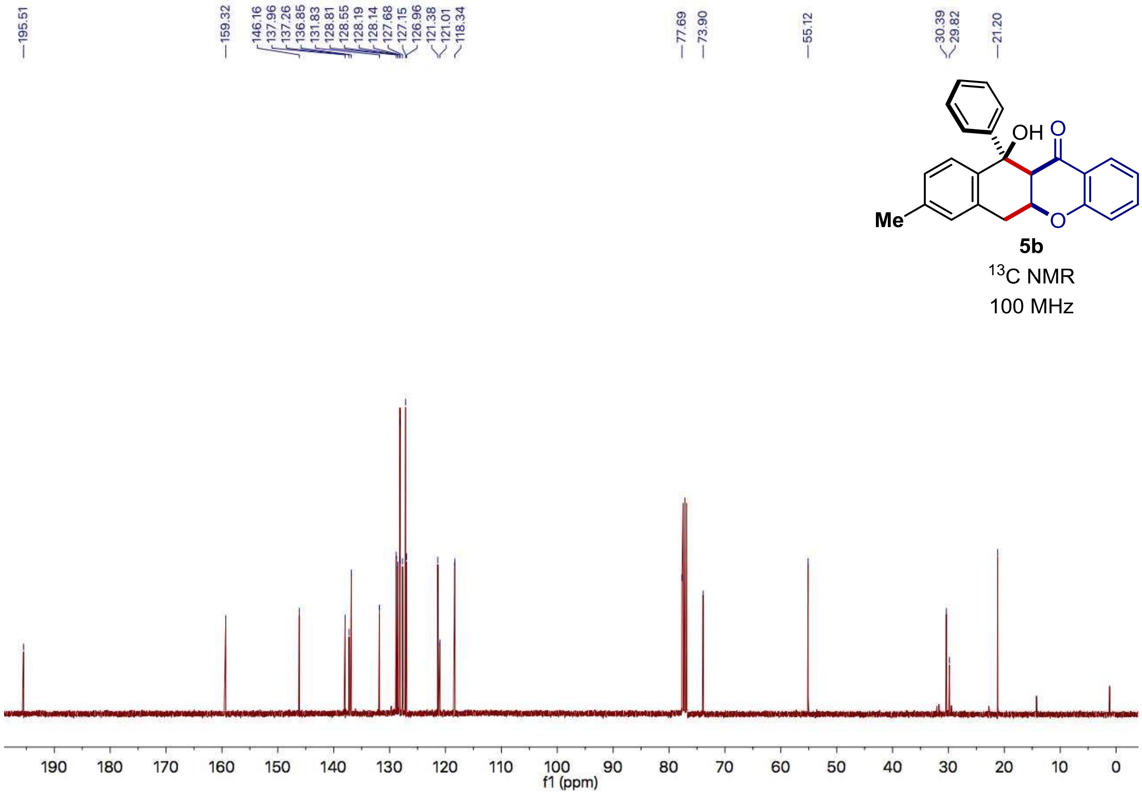

5c ^{1}H NMR spectrum

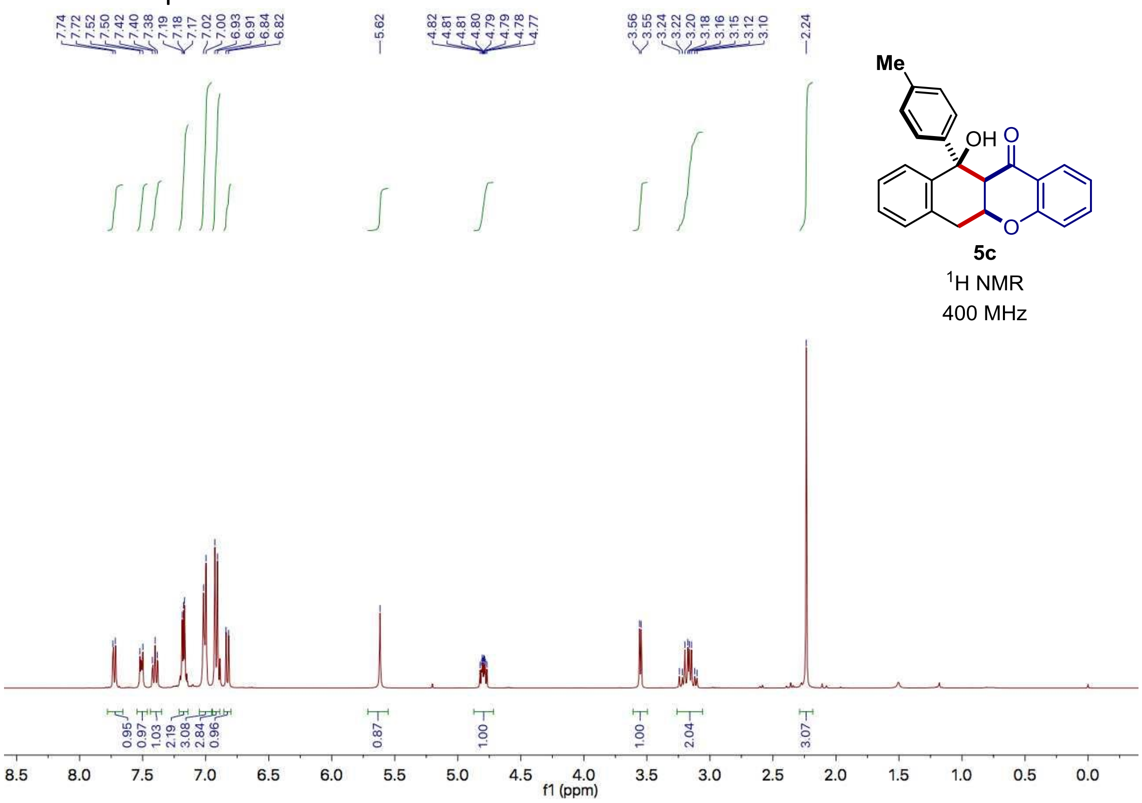

5c ^{13}C NMR spectrum

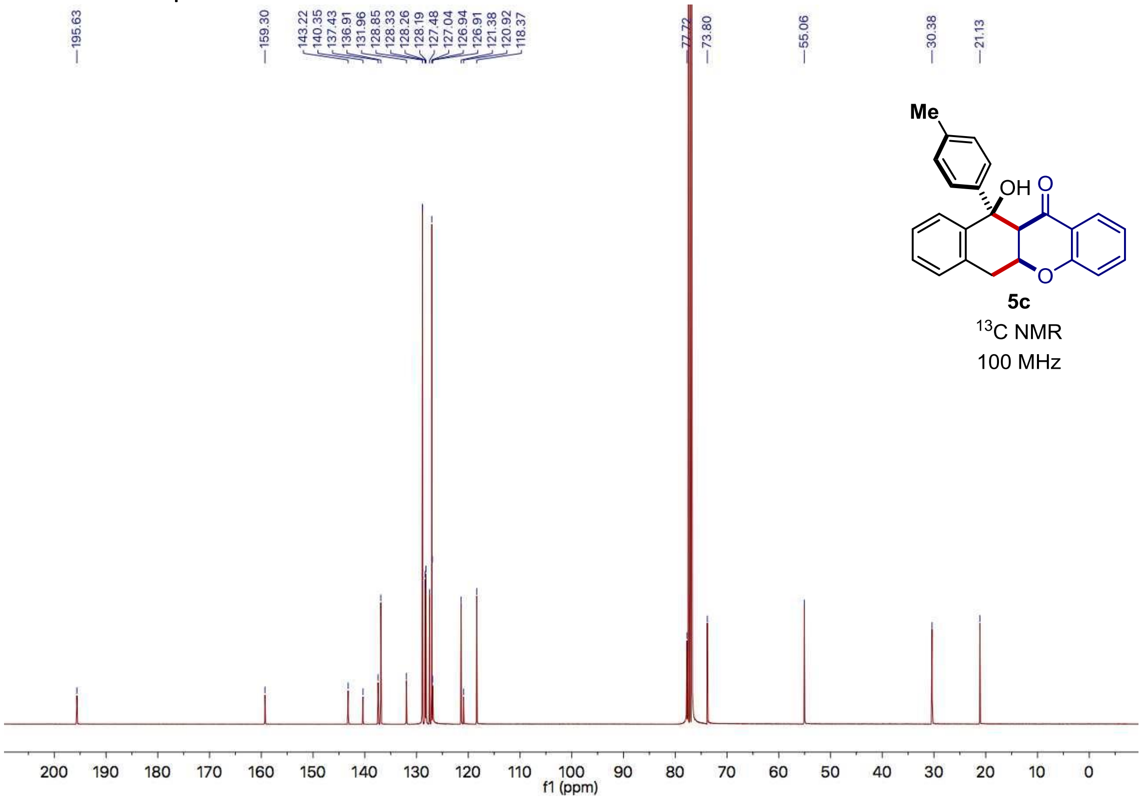

5d ^{1}H NMR spectrum

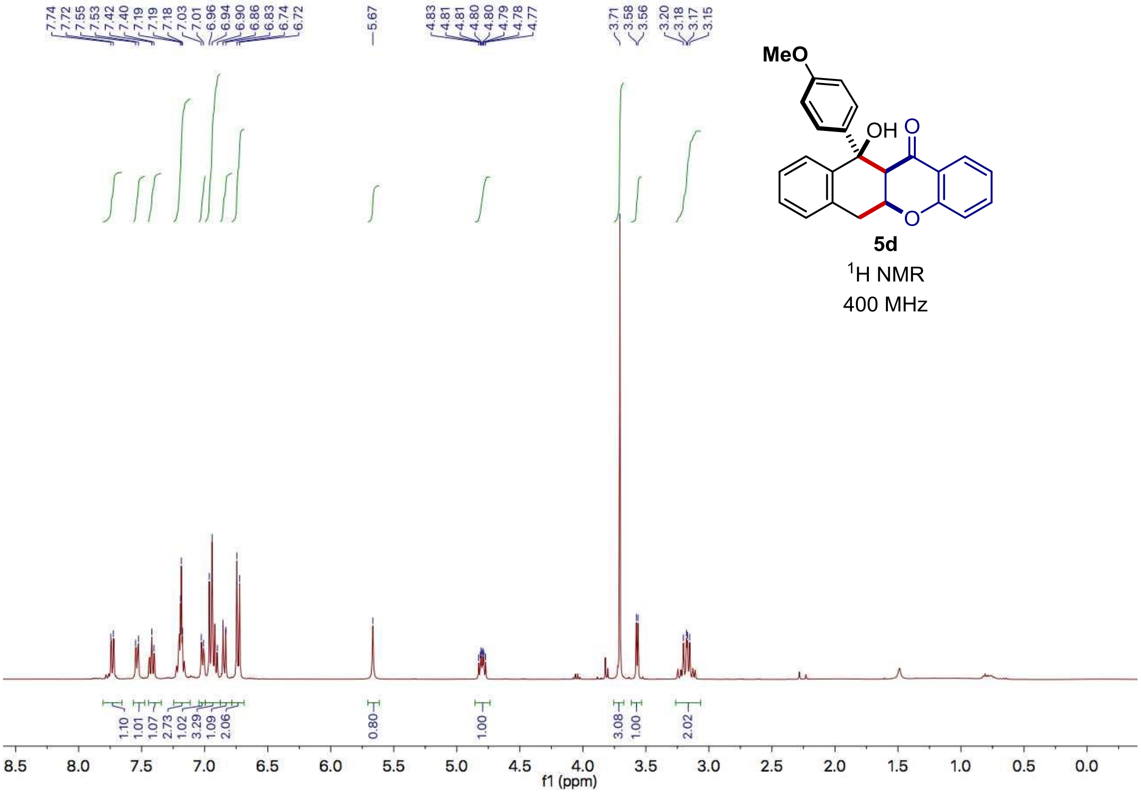

5d ^{13}C NMR spectrum

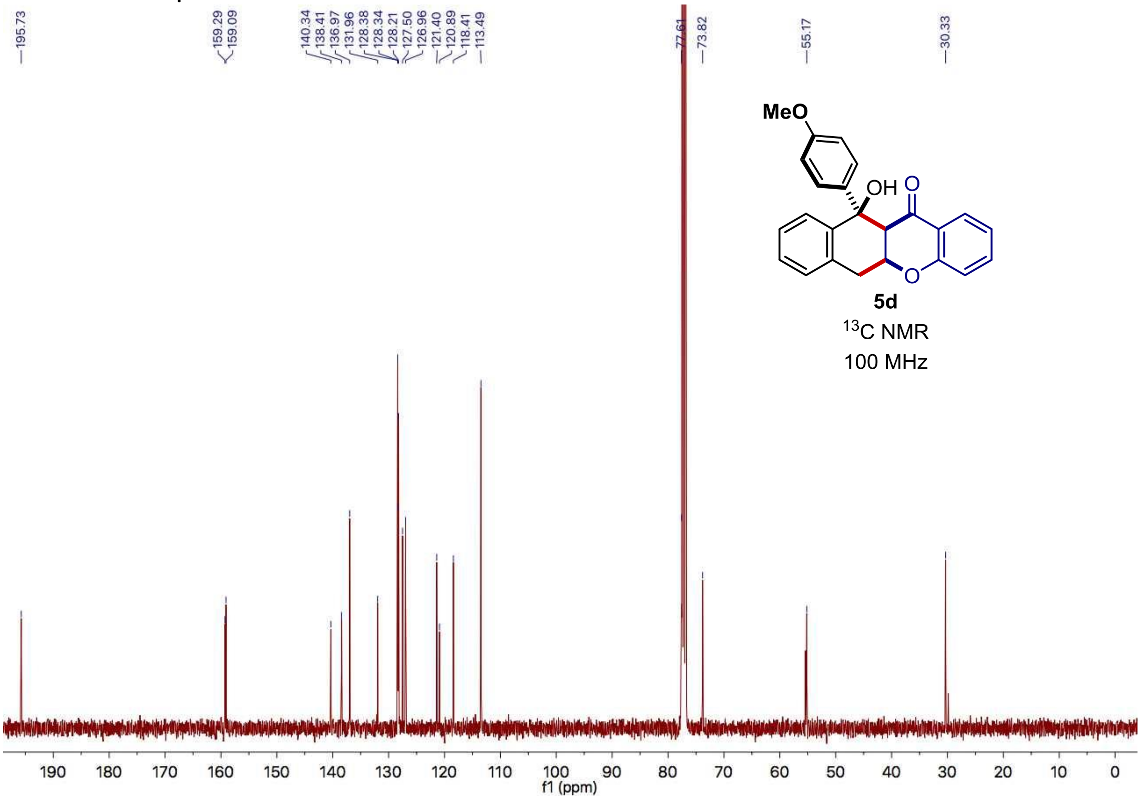

5e ¹H NMR spectrum

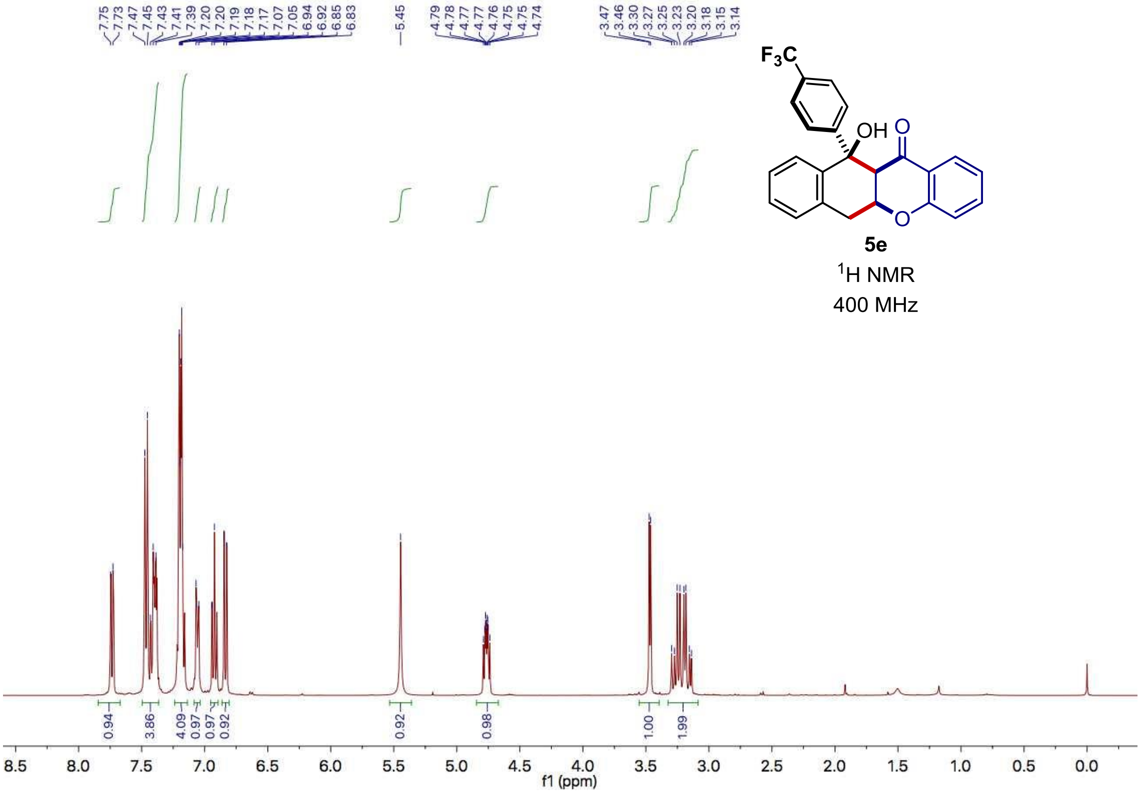

5e ¹³C NMR spectrum

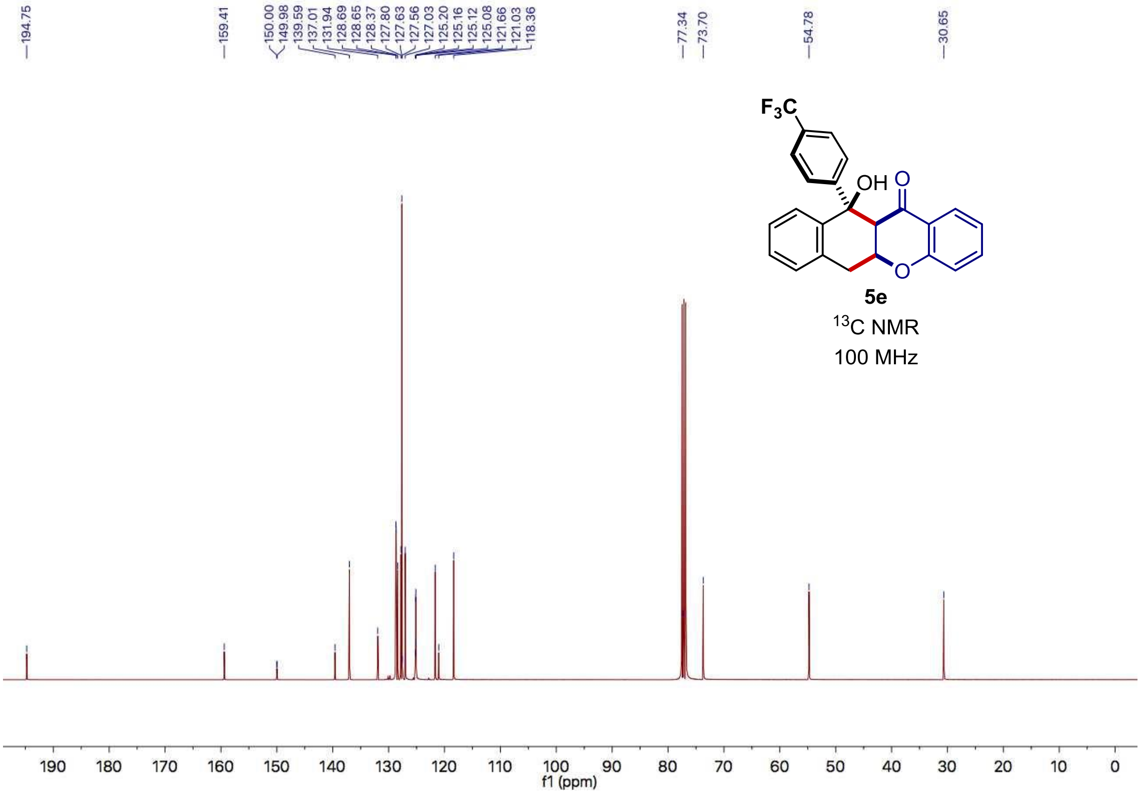

5f ^{1}H NMR spectrum

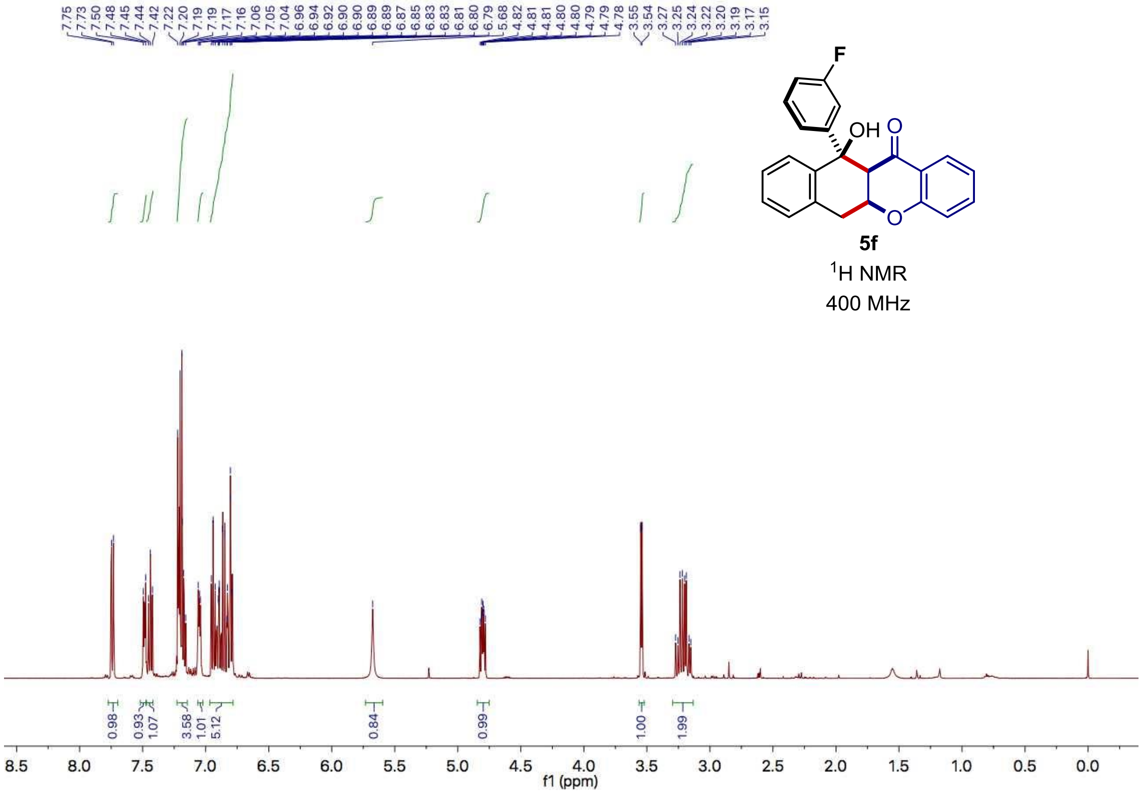

5f ^{13}C NMR spectrum

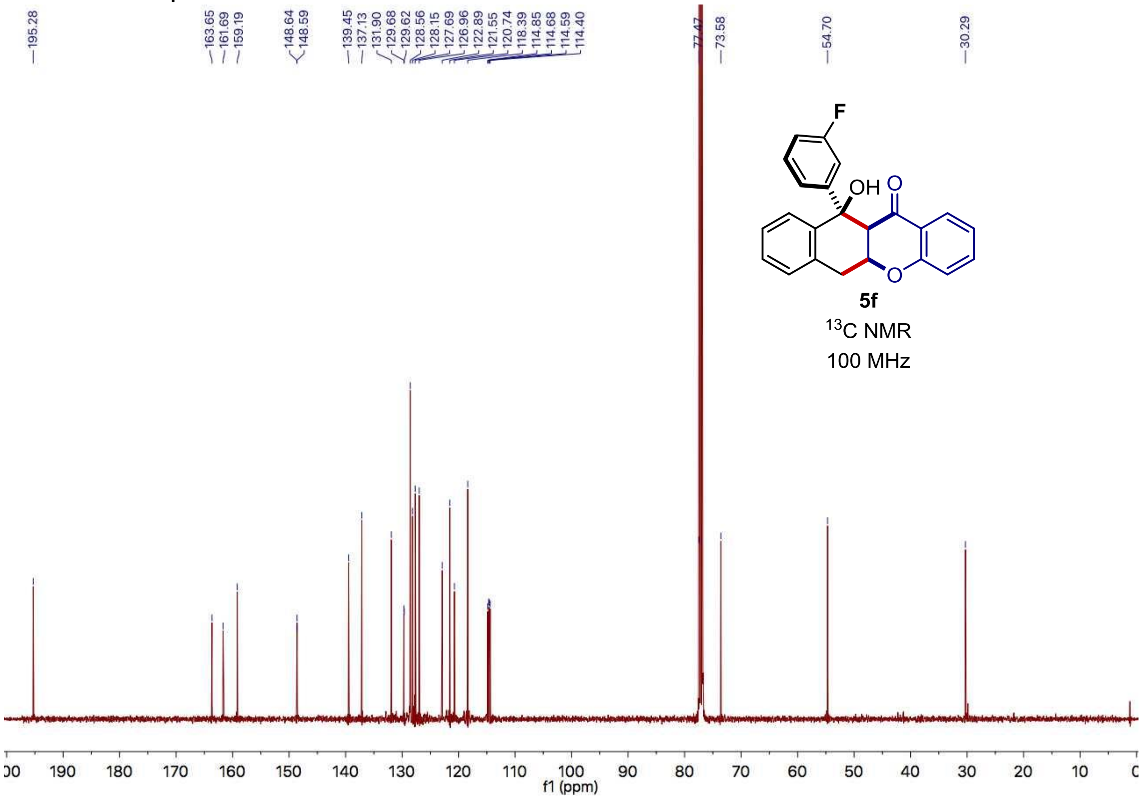

6a ^{1}H NMR spectrum

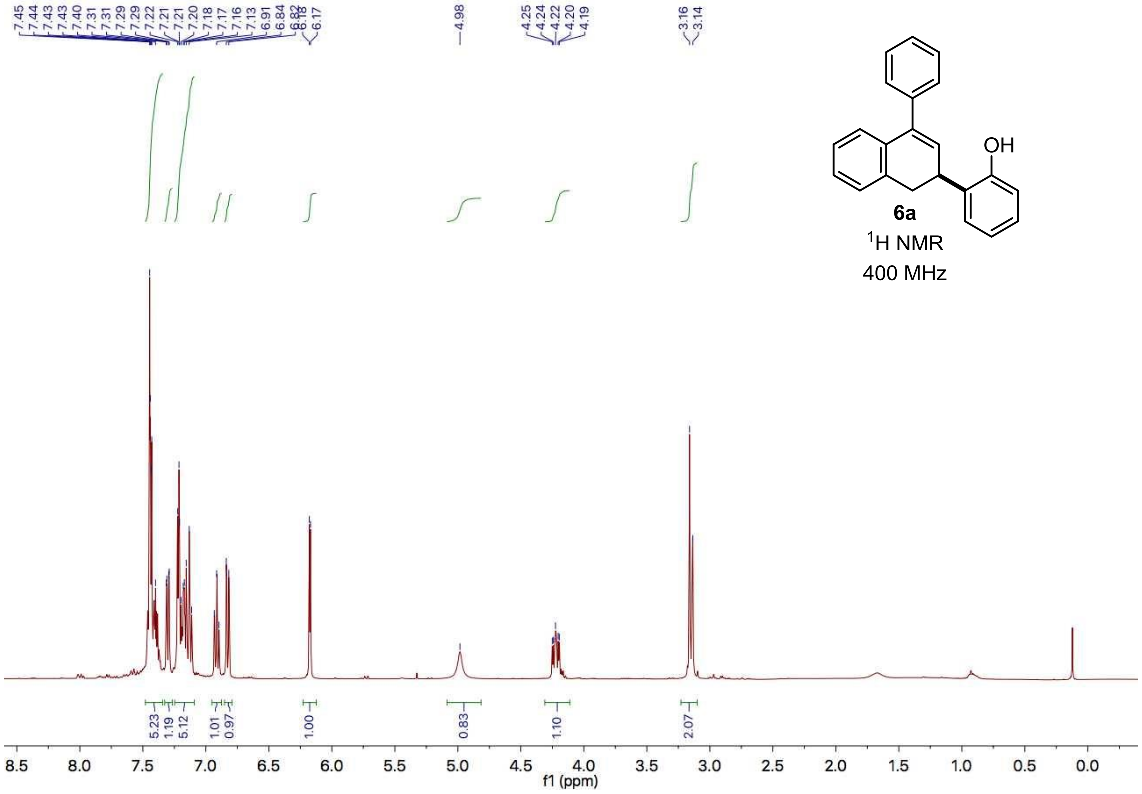

6a ^{13}C NMR spectrum

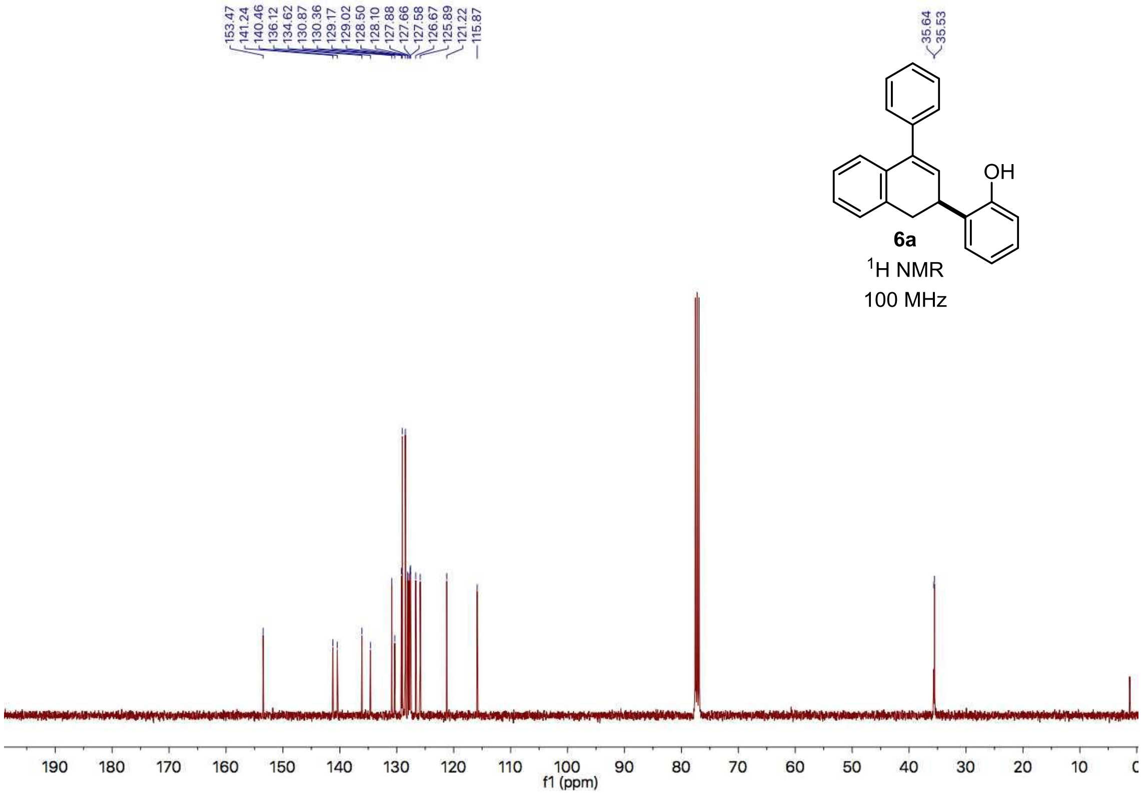

7a ¹H NMR spectrum

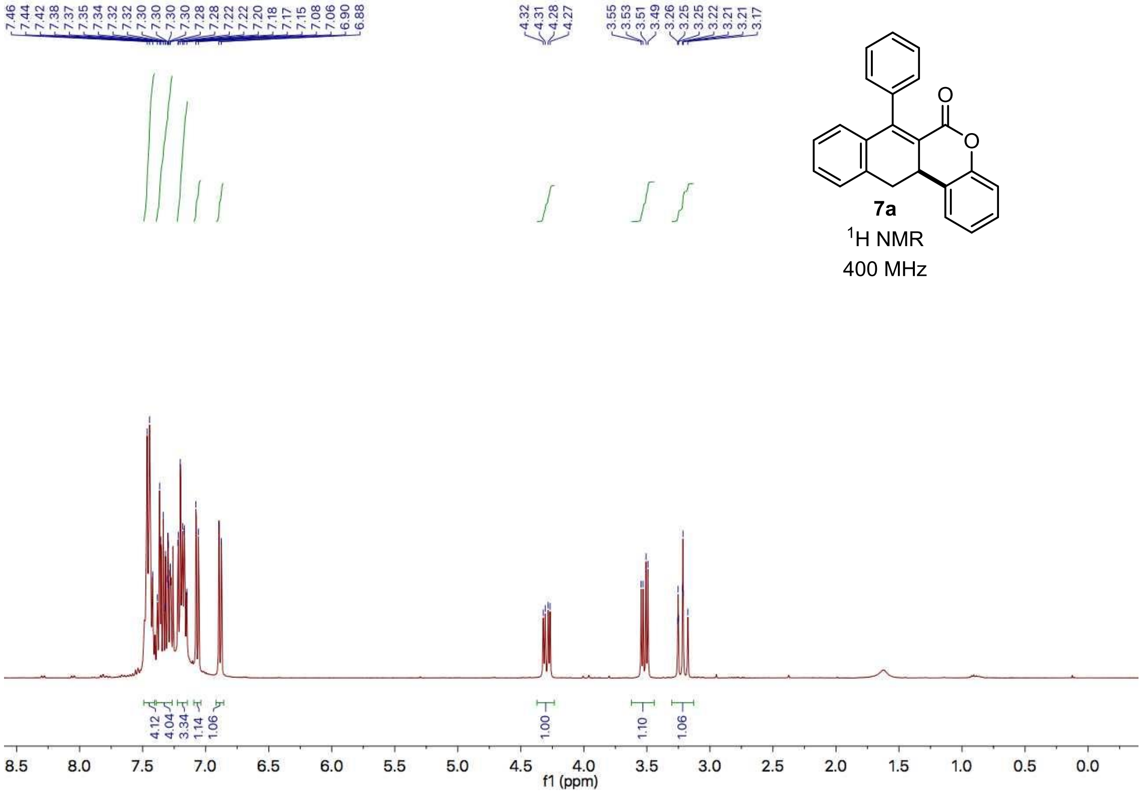

7a ¹³C NMR spectrum

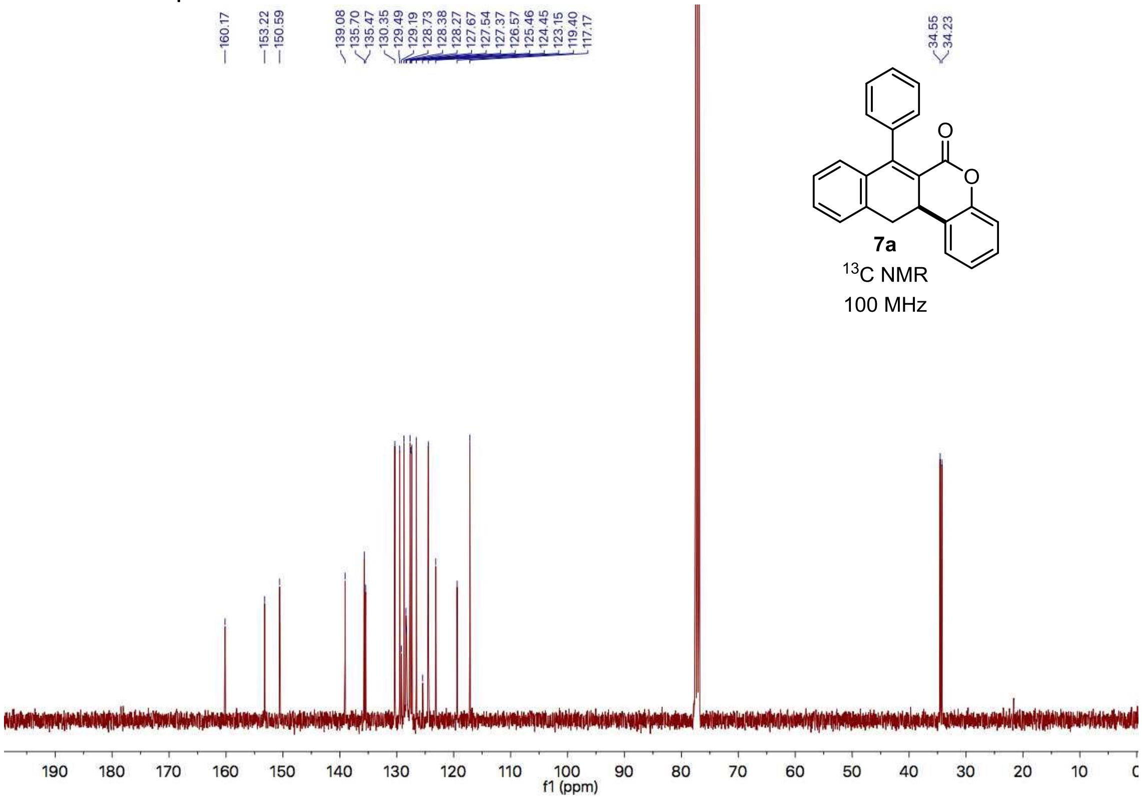

8a ^{1}H NMR spectrum

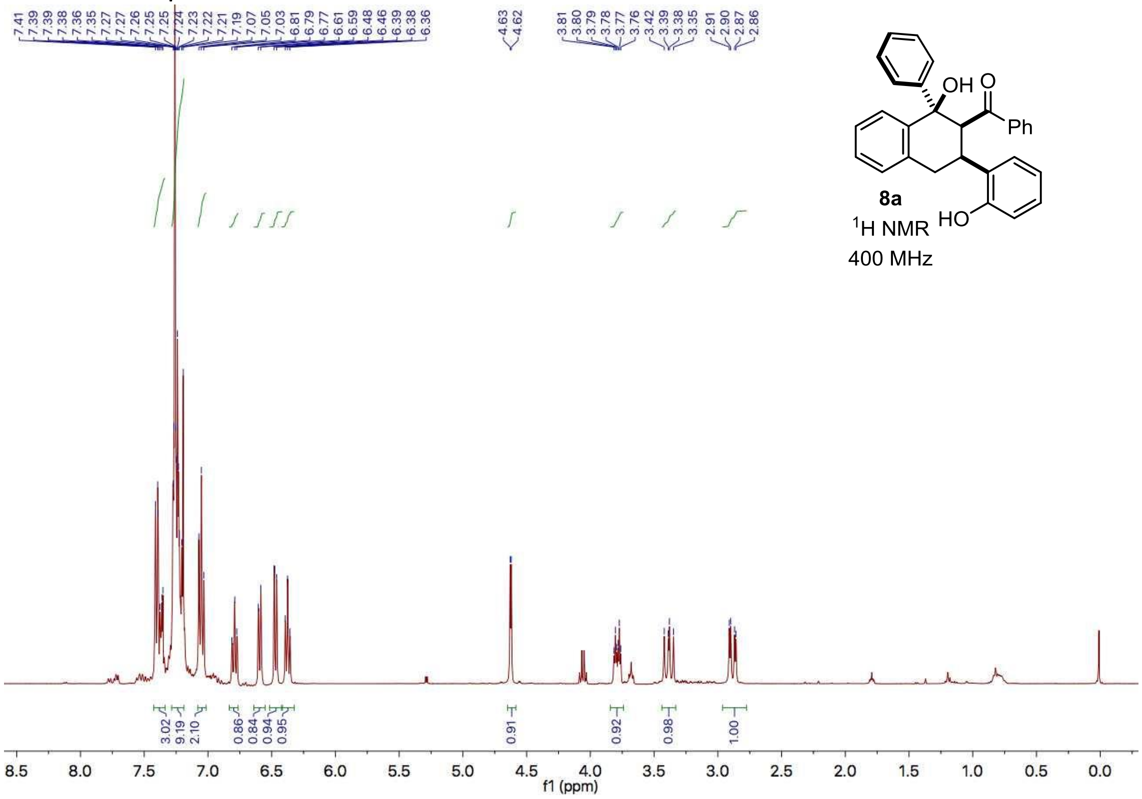

8a ^{13}C NMR spectrum

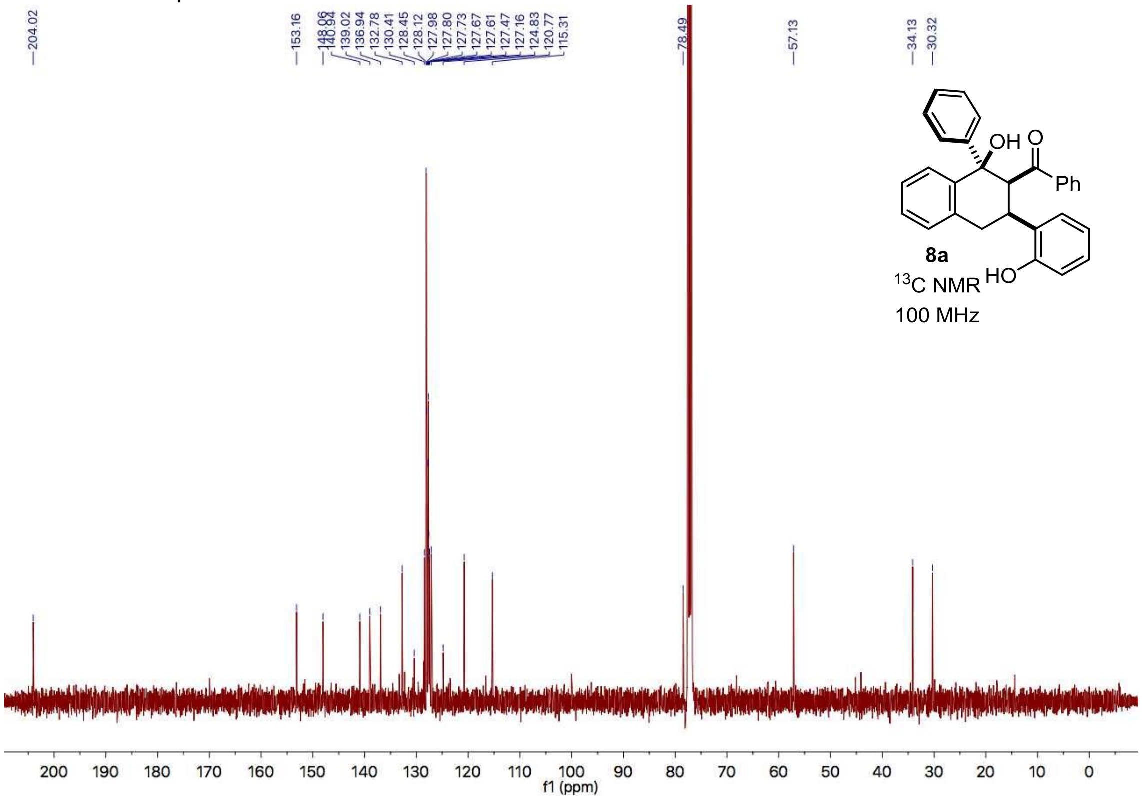

9a ^{1}H NMR spectrum

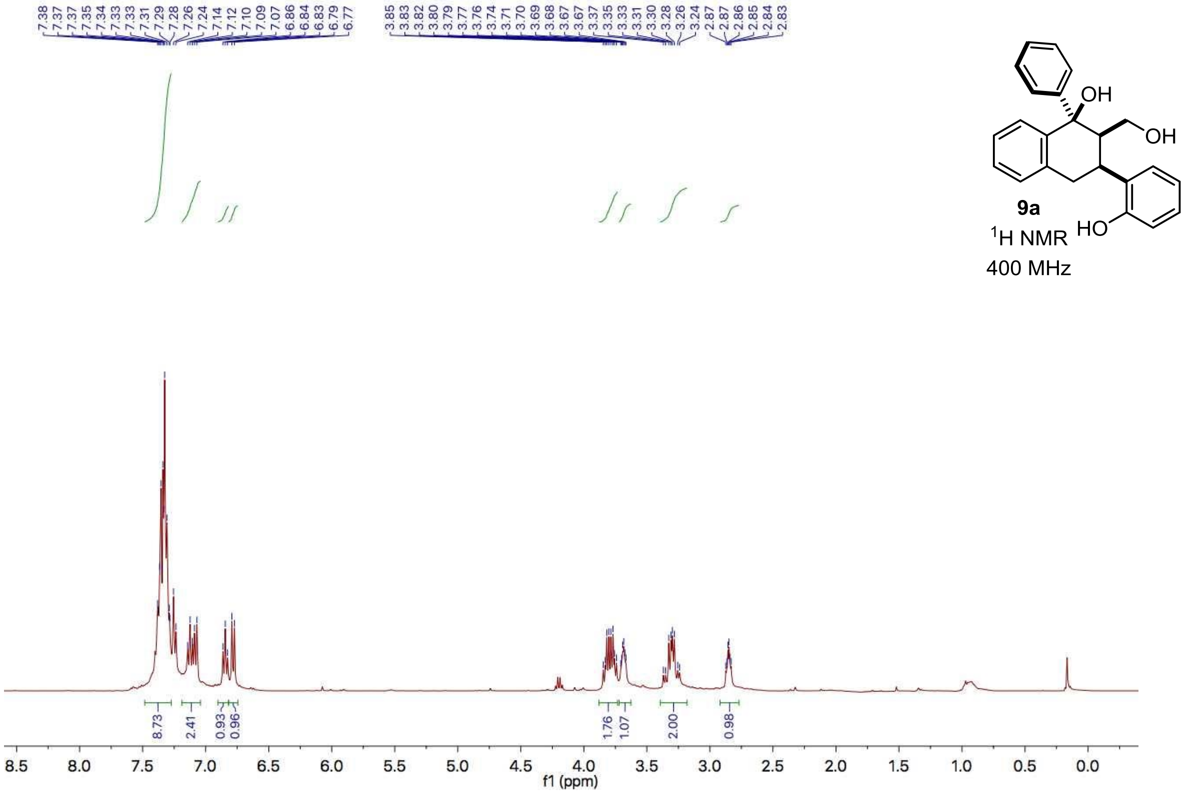

9a
^{1}H NMR
400 MHz

9a ^{13}C NMR spectrum

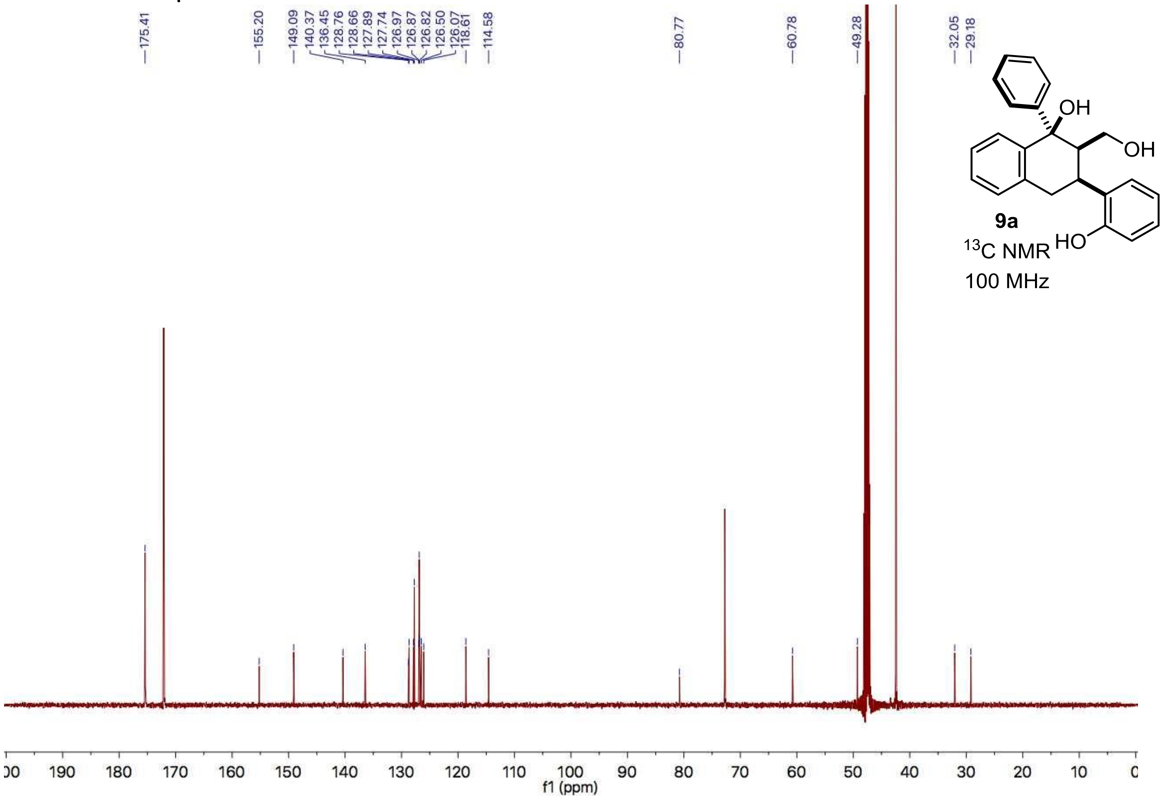

9a
^{13}C NMR
100 MHz
